# Meloxicam Alleviates Oxidative Stress Through Nrf2/HO-1 Activation in Bovine Endometrial Epithelial Cells

**DOI:** 10.3390/vetsci12060579

**Published:** 2025-06-12

**Authors:** Luying Cui, Jiangyao Duan, Peng Mao, Jingyi Zhong, Sasa He, Junsheng Dong, Kangjun Liu, Long Guo, Jianji Li, Heng Wang

**Affiliations:** 1 College of Veterinary Medicine, Yangzhou University, Jiangsu Co-Innovation Center for Prevention and Control of Important Animal Infectious Disease and Zoonoses, Yangzhou 225009, China; mx120221010@stu.yzu.edu.cn (J.D.); dx120220201@stu.yzu.edu.cn (P.M.); mx120221020@stu.yzu.edu.cn (J.Z.); mz120231724@stu.yzu.edu.cn (S.H.); junsheng@yzu.edu.cn (J.D.); kangjunliu@yzu.edu.cn (K.L.); yzdxgl@yzu.edu.cn (L.G.); jjli@yzu.edu.cn (J.L.); 2 International Research Laboratory of Prevention and Control of Important Animal Infectious Diseases and Zoonotic Diseases of Jiangsu Higher Education Institutions, Yangzhou University, Yangzhou 225009, China; 3 Joint International Research Laboratory of Agriculture and Agriproduct Safety of the Ministry of Education, Yangzhou 225009, China

**Keywords:** meloxicam, antioxidation, Nrf2/HO-1 activation, bovine endometrial epithelial cells

## Abstract

Meloxicam has been identified as an adjuvant therapeutic component in the management of bovine uterine diseases, exhibiting anti-inflammatory and antioxidant effects. However, the mechanisms underlying its antioxidant actions in the context of bovine uterine diseases remain incompletely understood. The objective of this research was to determine whether meloxicam exerts its antioxidant effects through the Nrf2/HO-1 signaling pathway. By employing N-acetylcysteine (NAC), a scavenger of reactive oxygen species (ROS), along with inhibitors directed against heme oxygenase-1 (HO-1) or nuclear factor erythroid 2-related factor 2 (Nrf2), we investigated the dynamic changes in oxidative stress markers (ROS and malondialdehyde) and antioxidant indices (comprising catalase, superoxide dismutase, and glutathione), as well as the expression profiles of Nrf2 and inflammation-associated genes and proteins in bovine endometrial epithelial cells (BEECs) subjected to lipopolysaccharide (LPS) stimulation. As a result, meloxicam alleviated the LPS-induced elevation of oxidative stress marker levels and the reduction in antioxidant enzyme activities and antioxidant substance contents in BEECs. Compared to NAC, meloxicam demonstrated superior efficacy in activating the Nrf2 pathway, with the promotion of NRF2 expression (~1.6-fold) and nuclear translocation. The pretreatment of cells with HO-1 or Nrf2 inhibitors markedly attenuated the antioxidant activity of meloxicam. In summary, meloxicam primarily alleviates LPS-induced oxidative stress through the activation of the Nrf2/HO-1 pathway in BEECs.

## Simple Summary

Oxidative stress in cattle can lead to uterine diseases. Meloxicam possesses anti-inflammatory and antioxidant properties and plays a crucial role in the adjuvant treatment of uterine diseases in dairy cows. This research explored how meloxicam influences oxidative stress responses and their underlying mechanisms in the epithelial cells of the bovine endometrium. We found that meloxicam protected cells from oxidative stress by activating the Nrf2/HO-1antioxidant pathway. This research contributes to elucidating the mechanism underlying meloxicam’s therapeutic action in the bovine endometrium.

## 1. Introduction

Postpartum uterine diseases represent a primary factor for the reduced reproductive performance in dairy cows. Following parturition in cattle, the uterine cavity is contaminated with bacteria, and the persistent colonization of pathogenic microorganisms can induce inflammatory conditions [1]. These inflammatory states contribute to endometrial histological alterations, delayed uterine involution, and compromised embryo viability [2,3]. Furthermore, the negative energy balance during the postpartum period exacerbates inflammatory-mediated tissue damage in the bovine endometrium [4]. Even after clinical recovery, reproductive challenges, such as reduced conception rates, may persist and ultimately lead to substantial economic losses. Consequently, strategies focusing on inflammation relief and minimizing tissue damage are of paramount significance to maintain the reproductive performance of dairy cows.

Meloxicam is an enolic non-steroidal anti-inflammatory drug (NSAID) with anti-inflammatory, analgesic, and antipyretic effects that demonstrates high therapeutic potential for diseases such as osteoarthritis, puerperal fever, mastitis, and endometritis in various species [5]. In clinical practice, meloxicam has been proven to show therapeutic effects in the management of mastitis and provide analgesia during the puerperal period in dairy cows [6,7]. In cases of cesarean section or dystocia, meloxicam helps alleviate parturient stress, reduce postpartum culling rates, and improve the overall health of the animal [8]. In the treatment of metritis and endometritis, meloxicam can be used, where appropriate, as an adjunct to antibiotics (such as oxytetracycline or ceftiofur) to relieve the systemic inflammatory responses and shorten the treatment duration [9]. In addition, the application of meloxicam reduces the incidence of ketosis and alleviates negative energy balance in peripartum cows [10]. Meloxicam’s selective inhibition of cyclooxygenase-2 (COX-2), which catalyzes arachidonic acid’s conversion to prostaglandins, is well established [11]. This effect has been documented in bovine mammary epithelial cells exposed to *Escherichia coli* lipopolysaccharide (LPS) and *Staphylococcus aureus* lipoteichoic acid and in bovine endometrial epithelial cells (BEECs) challenged with LPS [12,13]. Whether meloxicam exerts therapeutic effect through other mechanisms is less well investigated.

Studies exploring the role of oxidative stress in endometrial physiology have strongly indicated a connection between inflammation and oxidative stress [14]. In the early stages of inflammation, inflammatory cells release a large amount of free radicals, such as reactive oxygen species (ROS) [15]. The overproduction and limited elimination of free radicals lead to oxidative stress. As ROS mediates oxidative damage to biomolecules, the accumulation of ROS results in the production of lipid peroxidation products, such as malondialdehyde (MDA). Meanwhile, oxidases and antioxidants, such as catalase (CAT), glutathione (GSH), and superoxide dismutase (SOD), are vital components of the body’s antioxidant defense system [16]. Meloxicam has been found to exert antioxidative effects. It has been shown that meloxicam alleviated oxidative stress in liver tissue by suppressing the decrease in CAT, SOD, and GSH levels in rats with hepatic fibrosis [17]. In an LPS-induced oxidative stress model of bovine endometrial epithelial cells, meloxicam reduced ROS and MDA concentrations and enhanced *CAT* and *SOD1* mRNA expression [13].

Nuclear factor erythroid-2-related factor 2/heme oxygenase 1 (Nrf2/HO-1) signaling is essential for antioxidant defenses [13]. Upon activation, the Nrf2 protein enters the nucleus and promotes the synthesis of Nrf2-dependent genes encoding antioxidant enzymes, including NAD(P)H quinone dehydrogenase 1 (NQO1) and heme oxygenase 1 (HO-1) [18]. It has been shown that meloxicam increased the Nrf2 protein and intracellular GSH levels in human neuroblastoma SH-SY5Y cells treated with paraquat [19]. Meloxicam was found to improve depression-like neuropathology by stimulating the Nrf2/HO-1 pathway in rats with chronic restraint stress [13]. Previous research in our laboratory found that meloxicam alone alleviated oxidative damage through Nrf2 activation in bovine endometrial epithelial cells challenged with LPS [20]. However, whether meloxicam exerts antioxidant effects through the Nrf2/HO-1 signaling pathway remains to be verified.

This study aims to clarify the role of the Nrf2/HO-1 pathway in meloxicam antioxidation, thereby elucidating the specific mechanism of action and providing new insights into the application of meloxicam in the management of bovine uterine diseases. The primary BEECs were stimulated with LPS to establish an in vitro oxidative stress model. With the application of specific pathway inhibitors, the effect of meloxicam on cellular oxidative status was assessed by observing the changes in the oxidative stress indicators (ROS and MDA), the related antioxidant enzymes and substances (SOD, CAT, and GSH), and Nrf2/HO-1 signaling.

## 2. Materials and Methods

### 2.1. Drugs and Reagents

LPS from *Escherichia coli* O111 was purchased from Beyotime (S1732, Shanghai, China). Meloxicam was purchased from AbMole (M3297, Shanghai, China). Acetyl-L-cysteine (NAC) was produced by Sigma (A7250-5G, Sigma, St. Louis, MO, USA). ML385 was obtained from Med Chem Express (HY-100523, Shanghai, China). Zinc protoporphyrin (ZnPP) was purchased from Med Chem Express (HY-101193, Shanghai, China).

### 2.2. Cell Culture

The primary endometrial epithelial cells were isolated and cultured using the previously described method [21]. Briefly, uteri from non-pregnant dairy cows without reproductive diseases were collected at a slaughterhouse and sent to the laboratory as soon as possible. The cervical opening was tied tightly with cotton thread, and the uterus was fully immersed in refrigerated iodophor solution before being transported back to the laboratory. In a laminar flow hood, the uterine horns were cut open to expose the endometrium, which was then cut into small segments of about 3 cm. These segments were rinsed with pre-cooled phosphate-buffered saline (PBS) containing penicillin and streptomycin until the liquid was clear and then soaked in a 4 °C refrigerator for 30 min. Afterwards, they were placed in DMEM/F12 serum-free medium containing 1 mg/mL protease stock solution (P5147, Sigma, St. Louis, MO, USA) for the digestion at 4 °C for 16 h. The tissues were then removed from the soaking solution, and the endometrial epithelium was gently scraped off in a sterilized Petri dish. The scraped endometrium was repeatedly washed and centrifuged at 1000× *g* with PBS until the liquid was clear. The collected cells were cultured in DMEM/F12 medium (D8900, Sigma, St. Louis, MO, USA), which was maintained at 37 °C in a 5% CO_2_ environment and supplemented with 100 units/mL penicillin, 100 μg/mL streptomycin, and 15% fetal bovine serum (S711-001S, LONSERA, Suzhou, China). Under a microscope (Olympus, Tokyo, Japan), the medium was replaced every 24~48 h after the cells adhered to the surface; the cells were subcultured according to their growth status. Cells that had been passaged to the 3rd to 5th generation were used for subsequent experiments.

### 2.3. Treatment Design

To answer the question of whether meloxicam alleviates BEEC oxidative damage through ROS scavenging, we applied the ROS scavenger NAC. The cells were allocated into five treatment groups: the blank control, the LPS group, the LPS and meloxicam co-treatment group (LPS + MEL), the LPS and NAC co-treatment group (LPS + NAC), and the LPS, meloxicam, and NAC co-treatment group (LPS + MEL + NAC). Based on previous results from our laboratory, LPS of 10 μg/mL is able to induce BEEC oxidative stress and inflammation [21,22]. Due to the fact that the maximum plasma concentration of meloxicam reaches 1.68 μg/mL (4.78 μM) in dairy cows after its oral administration [23] and that 5 μM meloxicam showed no influence on BEEC viability [21], we chose to use 5 μM meloxicam in the current experiment. The NAC level in human plasma generally falls within the range of 300 to 900 mg/L (1.8 and 5.5 mM) [24]. In cell culture experiments, the most frequently employed NAC concentrations fall within the 1~10 mM range. Notably, in studies involving bovine oocytes, it was observed that supplementing the culture medium with NAC at concentrations exceeding 10 mM led to a decrease in pH, which proved challenging to restore and stabilize at physiologically relevant levels [25]. Consequently, we chose to use 5 mM NAC for this experiment. The cells were collected 12 h after treatment.

Next, to investigate whether meloxicam alleviates BEEC oxidative damage through activating the Nrf2/HO-1 pathway, we pretreated the cells with the Nrf2 inhibitor ML385 or the HO-1 inhibitor ZnPP for 12 h. The experimental design is presented below: the blank control, the LPS treatment, the combined treatment of LPS and meloxicam (LPS-MEL), the combined treatment of LPS and inhibitor (LPS-ML385, or LPS-ZnPP), and the combined treatment of LPS, meloxicam, and inhibitor (LPS-MEL-ML385 or LPS-MEL-ZnPP).

### 2.4. Cell Viability

The impact of NAC, ML385, and ZnPP on BEEC viability was evaluated via a WST-8-based assay (R401, Vazyme, Nanjing China). The BEECs were inoculated at a density of 1 × 10^3^ cells per well in a 96-well plate. When 80% density was reached, the cells were incubated with various concentrations (0~10 μM) of NAC, ML385, and ZnPP for 24 h. Subsequently, the supernatant was removed. Next, 100 μL of complete medium and 10 μL of the assay reagent were added. After that, the BEECs were incubated at 37 °C for 2 h. Finally, a fully automatic microplate reader (Bio Tek, Winooski, VT, USA) was used to detect the absorbance at 450 nm.

### 2.5. Flow Cytometry Analysis of ROS

The intracellular ROS level was quantified by a ROS Assay Kit (S0033S, Beyotime, Shanghai, China). The BEECs were inoculated at a density of 1 × 10^6^ cells per well in a 6-well plate. Following treatment, each well was digested with 200 μL trypsin, and then 400 μL serum-containing medium was added. The digested cells were transferred into 1.5 mL Eppendorf tubes and centrifuged at 1000× *g* for 5 min. Meanwhile, the assay reagent was diluted 1:1000 with serum-free medium. The supernatant was discarded from the Eppendorf tubes, and 1 mL of the diluted reagent was added to each tube to resuspend the cell pellet. The samples were then incubated for 30 min, with gentle inversion and mixing every 3 to 5 min. Following centrifugation, the cell pellet was washed repeatedly to thoroughly eliminate unincorporated assay reagent. The ROS content was analyzed using a FACScan flow cytometer (CytoFLEX, Becton Dickinson), and the data were processed with FlowJo™ v10.8 Software (BD Life Sciences, Franklin Lakes, NJ, USA).

### 2.6. Determination of the MDA, SOD, CAT, and GSH Levels

The BEECs were inoculated at a density of 1 × 10^6^ per well in a 6-well plate. After experimental treatment, the cells were transferred into 1.5 mL Eppendorf tubes and sonicated on ice, followed by centrifugation at 1000× *g* for 10 min to obtain the cell-free supernatant. The protein concentration in the supernatant was quantified by the bicinchoninic acid method (P0010, Beyotime, Shanghai, China). The assay kits for detecting MDA (S0131S), total SOD (S0101S, WST-8 method), CAT (S0051), and GSH (S0053) were all purchased from the Beyotime Biotechnology Co., Ltd. (Shanghai, China). Measurement of MDA, SOD, CAT, and GSH levels was conducted following the protocols of the respective assay kits.

### 2.7. Real-Time Quantitative PCR

The BEECs were inoculated at a density of 1 × 10^6^ cells per well into a 6-well plate. After treatment, the cells were rinsed three times with cold PBS. Total RNA was extracted using TRNzol Universal (R401-01, Vazyme, Nanjing, China) following the manufacturer’s instructions. The concentration and purity of the extracted RNA were determined with a spectrophotometer (Nanodrop 2000, Thermo Fisher Scientific, Waltham, MA, USA). The OD260/OD280 ratio for each sample ranged from 1.8 to 2.1. Approximately 1 μg of total RNA was converted into cDNA using a cDNA synthesis kit (AU341-02-V2, TransGen Biotech, Beijing, China). The reaction mixture was prepared according to the manufacturer’s instructions and contained 2 μL cDNA template, 10 μL reagent (Q311-02/03, Vazyme, Nanjing, China), 1 μL each of forward and reverse gene-specific primers at pM concentration, and double-distilled water. The relative abundance of these gene transcripts was analyzed using the 2^−ΔΔCt^ method, and each target gene was normalized using the internal reference Actin-beta (*ACTB*).

The target genes and the corresponding primer sequences are shown in Table 1.

### 2.8. Western Blot Analysis

Cells with densities of 5 × 10^6^ and 5 × 10^7^ were seeded into 60 mm and 100 mm dishes, respectively. After treatment, the total proteins of the cells were collected and extracted using a cell lysis buffer (C1053, Applygen, Beijing, China) containing a protease inhibitor cocktail (P1265, Applygen, Beijing, China) and a phosphatase inhibitor cocktail (P1260, Applygen, Beijing, China). The nuclear protein was obtained from the treated cells following the protocol of the relevant extraction kit (P0028, Beyotime, Shanghai, China). Then, the bicinchoninic acid method was employed to measure the protein concentration. The target protein (10 μg) was separated by sodium dodecyl sulfate-polyacrylamide gel electrophoresis and transferred onto a polyvinylidene fluoride membrane (Millipore, Bedford, MA, USA). The membrane was then blocked in 5% non-fat milk for 2 h and washed three times with Tris-buffered saline containing 0.05% Tween-20 for 8 min each time. Subsequently, the blocked membrane was treated with a solution containing the primary antibody at 4 °C for 12 h. The primary antibodies were specific for NRF2 (1/1000 dilution, #AF0639, Affinity, Liyang, China), COX-2 (1/1000 dilution, #12375-1-AP, Proteintech, Wuhan, China), HO-1 (1/1000 dilution, #10701-1-AP, Proteintech, Wuhan, China), NQO1 (1/10,000 dilution, #DF6437, Affinity, Liyang, China), P65 (1/1000 dilution, #AF5006, Affinity, Liyang, China), β-actin (1/10,000 dilution, #20534-1-AP, Proteintech, Wuhan, China), and Lamin B1 (1/10,000 dilution, #12987-1-AP, Proteintech, Wuhan, China). After washing, the membrane was incubated with secondary antibodies diluted in 5% fat-free milk at 20 °C for 1.5 h. Then, the bands were visualized using a chemiluminescence reagent (Abbkine, Wuhan, China) with the application of a chemiluminescence imaging system (Clinx Science Devices, Shanghai, China). The intensity of each band was quantified via ImageJ software (Version 1.38e, National Institutes of Health, Bethesda, ML, USA), and the levels of the target protein were normalized to β-actin.

### 2.9. Immunofluorescence Staining

Cells with a density of 5 × 10^5^ cells per well were seeded into a 24-well plate. The BEECs grown on coverslips in 24-well plates were washed three times with PBS. After aspirating the PBS, each well was supplemented with 500 μL of 4% paraformaldehyde (BL539A, Biosharp, Hefei, China) for cell fixation on coverslips at 4 °C. The coverslips were then permeabilized with 0.5% Triton X-100 (ST797, Beyotime, Shanghai, China) at 20 °C for 20 min. Then, each well was supplemented with 500 μL bovine serum albumin (5%), and the coverslips were blocked at 37 °C for 30 min. The bovine serum albumin blocking solution was aspirated, and 500 μL of the diluted primary antibody was added to each well. The plates were positioned in a humidified chamber and incubated overnight at 4 °C. After aspirating the primary antibody, the coverslips were rinsed. Excess liquid was blotted away, and 500 μL of the diluted fluorescent secondary antibody was added to each well. The plates were kept in the dark at 37 °C for 1 h. After the rinsing with PBS, 500 μL DAPI (P0131, Beyotime, Shanghai, China) was added to the coverslip and the coverslip incubated for 5 min in the absence of light. Mounted on glass slides, the cell coverslips were examined under a laser confocal microscope (Leica TCS SP8, Leica Microsystems, Wetzlar, Germany) to visualize the protein distribution. Nuclear fluorescence intensity was then quantified using ImageJ software (1.38e, National Institutes of Health, Bethesda, ML, USA).

### 2.10. Statistical Analysis

All experiments were independently replicated a minimum of three times. Data were analyzed using GraphPad Prism 9.0 (GraphPad Software, San Diego, CA, USA). Normality of the data was assessed via the Shapiro-Wilk test, with all datasets satisfying a normal distribution (all *p* > 0.05). Subsequently, one-way ANOVA with post hoc Dunnett’s or Tukey’s tests was performed to identify statistically significant differences. Results were reported as mean ± standard error of the mean (SEM), with statistical significance defined as *p* < 0.05 and highly significant differences denoted by *p* < 0.01.

## 3. Results

### 3.1. Meloxicam Inhibited ROS Accumulation and Increased the Levels of Antioxidant Indicators

**Figure 1:**
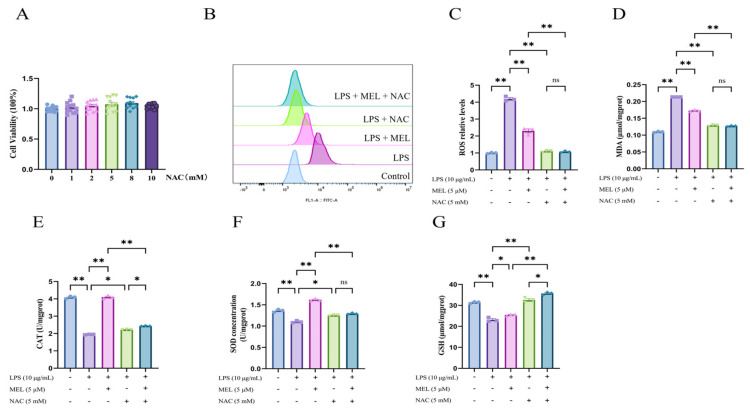
Meloxicam alleviated the oxidative stress of bovine endometrial epithelial cells stimulated with lipopolysaccharide. (**A**) Cell viability at 24 h was assessed in response to various concentrations of NAC. (**B**–**G**) Cells underwent co-treatment with lipopolysaccharide (LPS), meloxicam (MEL), and/or N-acetyl-L-cysteine (NAC) for 12 h to monitor alterations in ROS (**B**,**C**), MDA (**D**), CAT (**E**), SOD (**F**), and GSH (**G**). ROS, reactive oxygen species. MDA, malondialdehyde. GSH, glutathione. SOD, superoxide dismutase. CAT, catalase. The data are expressed as means ± SEM (*n* = 3 or *n* = 6), with * indicating *p* < 0.05, ** indicating *p* < 0.01, and ns indicating *p* > 0.05.

**Figure 2:**
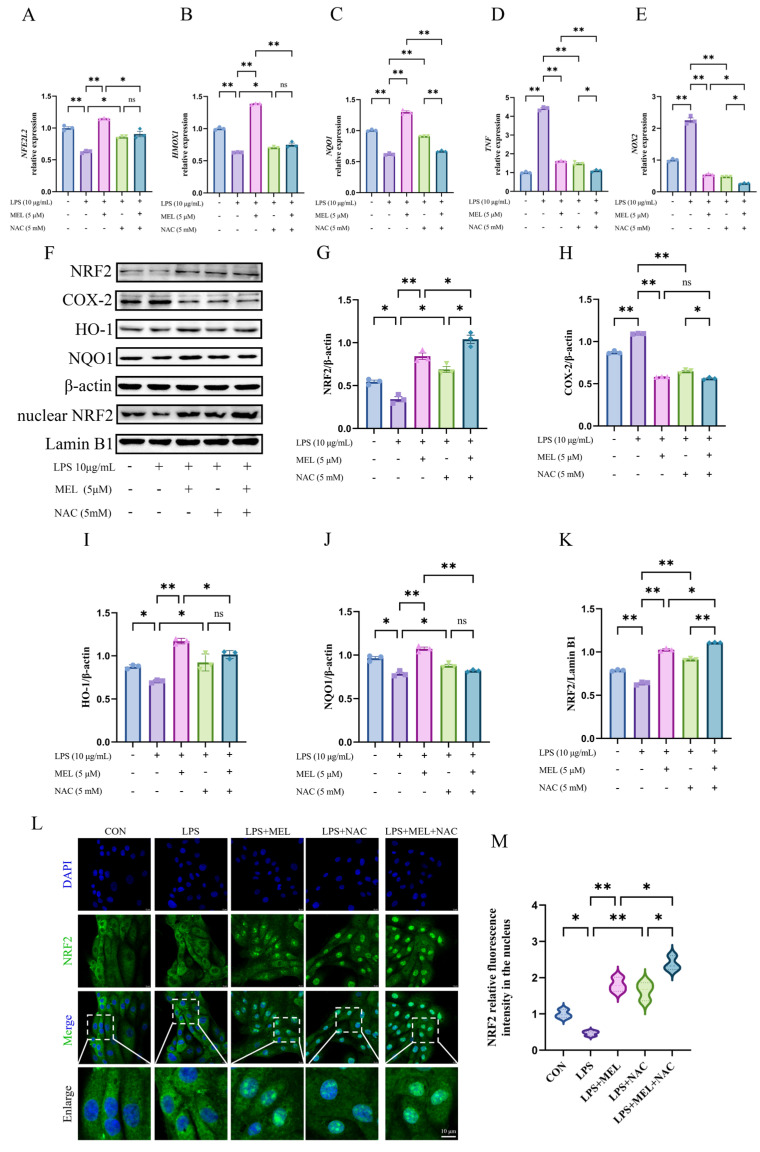
Meloxicam triggered Nrf2 signaling in the bovine endometrial epithelial cells challenged with lipopolysaccharide. The cells were subjected to the combined treatment with lipopolysaccharide (LPS), meloxicam (MEL), and/or N-acetyl-L-cysteine (NAC) for 12 h. Quantitative PCR was employed to assess alterations in the relative mRNA levels of *NFE2L2* (**A**), *HMOX1* (**B**), *NQO1* (**C**), *TNF* (**D**), and *NOX2* (**E**). Western blot (**F**) was utilized to determine the relative protein levels of total NRF2 (**G**), COX-2 (**H**), HO-1 (**I**), NQO1 (**J**), and nuclear NRF2 (**K**), where β-actin was used as an internal control. Immunofluorescence, with a scale bar of 10 μM, was used to visualize (**L**) and quantify (**M**) the amount of NRF2 protein in the nucleus. NRF2/*NFE2L2*, nuclear factor--erythroid 2 related factor 2. HO-1/*HMOX1*, heme oxygenase 1. NQO1, NAD(P)H quinone oxidoreductase 1. COX-2, cyclooxygenase-2. *NOX2*, nitric oxide synthase 2. *TNF*, tumor necrosis factor. The data are expressed as means ± SEM (*n* = 3), with * indicating *p* < 0.05, ** indicating *p* < 0.01, and ns indicating *p* > 0.05. Samples originated from the same experiment and blots were conducted in parallel. The original images of Western Blot and immunofluorescence can be found in File S1.

As shown in Figure 1A, 1, 2, 5, 8, and 10 mM NAC exerted no influence (*p* > 0.05) on BEEC viability. Compared with the control, LPS stimulated (*p* < 0.01) ROS and MDA production (Figure 1B–D). Under the condition of LPS treatment, both meloxicam and NAC were able to decrease (*p* < 0.01) the levels of ROS and MDA in BEECs. The levels of ROS and MDA were further reduced (*p* < 0.01) in the LPS + MEL + NAC group compared with those in the LPS + MEL group, whereas no difference (*p* > 0.05) was found in ROS and MDA between the LPS + NAC and the LPS + MEL + NAC groups.

As depicted in Figure 1E–G, LPS inhibited (*p* < 0.01) the amounts of CAT, SOD, and GSH, whereas such changes in CAT and SOD were fully reversed (~2.0-fold and ~1.6-fold, all *p* < 0.01) and the GSH concentration was partially reversed (~1.1-fold, *p* < 0.05) by the addition of meloxicam. Compared with the LPS group, there was a mild increase (~1.2-fold, *p* < 0.05) in the contents of CAT and SOD and a ~1.5-fold increase (*p* < 0.01) in the GSH concentration in the cells of the LPS + NAC group. When compared with the LPS + MEL group, in the LPS + MEL + NAC group, the levels of CAT and SOD decreased (*p* < 0.01), while the GSH level increased (*p* < 0.01). In comparison to the LPS + NAC group, the LPS + MEL + NAC group demonstrated a modest elevation (*p* < 0.05) in the levels of GSH and CAT. However, the SOD level in the LPS + MEL + NAC group did not show a statistically significant change.

### 3.2. Meloxicam Alleviated BEEC Oxidative Stress by Activating the Nrf2 Pathway

As shown in Figure 2A–E, LPS led to a decrease (~0.6-fold) in the relative mRNA levels of *NFE2L2*, *HMOX1*, and *NQO1* (all *p* < 0.01), along with a marked increase (*p* < 0.01) in *TNF* (~4.4-fold) and *NOX2* (~2.2-fold) expression. Although both meloxicam and NAC were able to reverse these changes, meloxicam seemed to cause a more pronounced increase (~2.0-fold, all *p* < 0.01) in antioxidant gene (*NFE2L2*, *HMOX1*, and *NQO1*) expression than that of NAC (~1.2-fold, *p* < 0.05). The attenuations (*p* < 0.05) of proinflammatory gene (*TNF* and *NOX2*) expression caused by meloxicam and NAC were of a similar extent. The LPS + MEL + NAC group exhibited a further reduction (*p* < 0.05) in all measured gene expressions compared to the LPS + MEL group but only exhibited reduced (*p* < 0.05) expression of *NQO1*, *TNF*, and *NOX2*, with no change (*p* > 0.05) in *NFE2L2* and *HMOX1* compared to the LPS + NAC group. The original images can be found in File S1.

The changes in the levels of key proteins of the Nrf2 pathway (total NRF2, HO-1, NQO1, and nuclear NRF2) and COX-2 are shown in Figure 2F–K. Exposure to LPS resulted in a reduction (*p* < 0.05) in the levels of key proteins of the NRF2 pathway and an upregulation (*p* < 0.05) of COX-2. Under LPS culture conditions, meloxicam enhanced (*p* < 0.01) the protein expression of NRF2, HO-1, and NQO1, with their levels being similar to those in the blank control group, whereas NAC treatment slightly increased the protein expressions of NRF2, HO-1, and NQO1 (all *p* < 0.05). Both meloxicam and NAC suppressed (*p* < 0.01) LPS-induced COX-2 protein expression. When meloxicam was added to the LPS and NAC co-treatment, the protein levels of both total NRF2 (~1.6-fold) and nuclear NRF2 (~1.2-fold) further increased (*p* < 0.05), while the COX-2 level slightly decreased (*p* < 0.05) and the levels of HO-1 and NQO1 remained unchanged (*p* > 0.05). In comparison with the LPS + MEL group, the combined treatment of LPS, MEL, and NAC exhibited higher (*p* < 0.05) levels of total NRF2 (~1.3-fold) and nuclear NRF2 (~1.1-fold) and lower (*p* < 0.05) levels of HO-1 (~0.9-fold) and NQO1 (~0.8-fold), whereas the level of COX-2 did not vary (*p* > 0.05).

Figure 2L,M depicts the changes in nuclear NRF2 distribution, which was generally in line with the Western blot results for the NRF2 protein. Under LPS culture conditions, both meloxicam and NAC promoted (*p* < 0.01) NRF2 nuclear translocation, and their combined application resulted in a more pronounced nuclear NRF2 accumulation compared to either treatment alone (both *p* < 0.01).

### 3.3. HO-1 Inhibitor Blunted the Antioxidant Effect of Meloxicam

**Figure 3:**
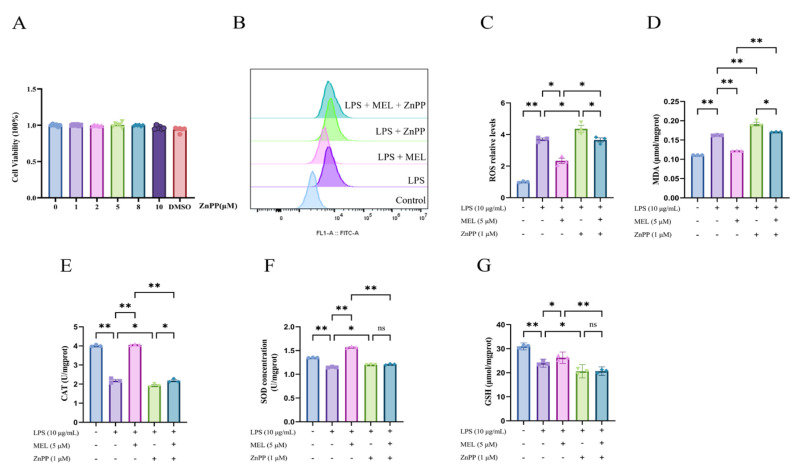
ZnPP reversed the antioxidative effect of meloxicam in bovine endometrial epithelial cells stimulated with lipopolysaccharide. (**A**) Cell viability at 24 h was assessed in response to various concentrations of ZnPP. (**B**–**G**) Cells underwent a 12 h pretreatment with ZnPP, after which they were co-treated with lipopolysaccharide (LPS) and meloxicam (MEL) for another 12 h to determine the changes in ROS (**B**,**C**), MDA (**D**), CAT (**E**), SOD (**F**), and GSH (**G**) levels. CAT, catalase. GSH, glutathione. MDA, malondialdehyde. ROS, reactive oxygen species. SOD, superoxide dismutase. ZnPP, zinc protoporphyrin. The data are expressed as means ± SEM (*n* = 3 or *n* = 6), with * indicating *p* < 0.05, ** indicating *p* < 0.01, and ns indicating *p* > 0.05.

As shown in Figure 3A, treating BEECs with 1, 2, 5, 8, and 10 μM ZnPP had no effect on cell viability (*p* > 0.05). Pretreatment with ZnPP abolished the suppressive effect of meloxicam on LPS-induced ROS and MDA production (LPS + MEL vs. LPS + MEL + ZnPP) and resulted in higher contents of ROS and MDA relative to the LPS group (LPS vs. LPS + ZnPP) (Figure 3B–D).

The elevation in the levels of CAT, SOD, and GSH induced by meloxicam was nullified (*p* < 0.01) by ZnPP pretreatment in LPS-stimulated BEECs (Figure 3E–G). In comparison to the LPS group, the cells in the LPS + ZnPP group showed a reduction (*p* < 0.05) in CAT and GSH contents. Conversely, the SOD content in BEECs increased (*p* < 0.05). When compared to the LPS + ZnPP group, the inclusion of meloxicam led to an elevation (*p* < 0.05) in the CAT level. However, the concentrations of GSH and SOD remained unchanged (*p* > 0.05).

### 3.4. HO-1 Inhibitor Weakened Meloxicam-Induced Nrf2 Activation

**Figure 4:**
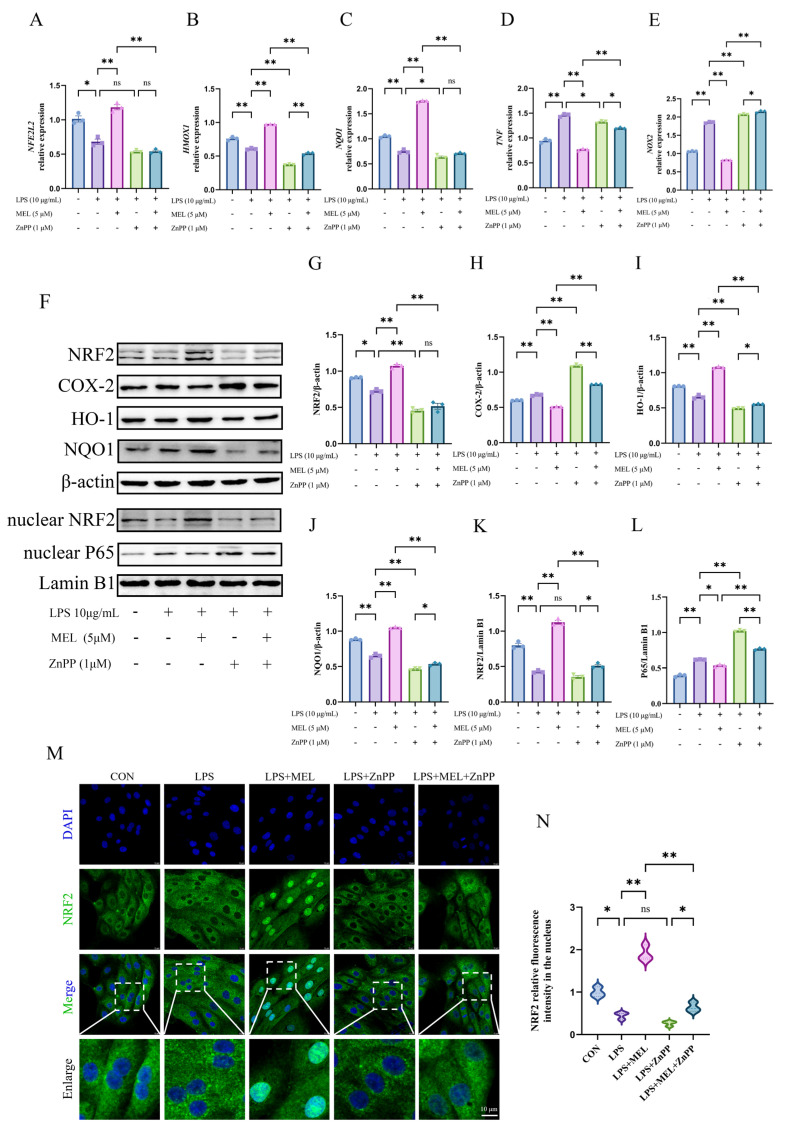
ZnPP weakened the meloxicam-induced Nrf2 activation in bovine endometrial epithelial cells stimulated with lipopolysaccharide. The cells were first subjected to a 12 h pretreatment with ZnPP. Subsequently, the cells underwent a combined treatment with lipopolysaccharide (LPS) and meloxicam (MEL) for 12 h. Quantitative PCR was then utilized to detect alterations in the relative mRNA levels of *NFE2L2* (**A**), *HMOX1* (**B**), *NQO1* (**C**), *TNF* (**D**), and *NOX2* (**E**). Western blot (**F**) analysis, with β-actin serving as the internal control, was employed to determine the relative protein levels of total NRF2 (**G**), COX-2 (**H**), HO-1 (**I**), NQO1 (**J**), nuclear NRF2 (**K**), and nuclear P65 (**L**). Immunofluorescence, with a scale bar of 10 μM, was used to visualize (**M**) and quantify (**N**) the nuclear NRF2 protein level. NRF2/*NFE2L2*, nuclear factor-erythroid 2 related factor 2. HO-1/*HMOX1*, heme oxygenase 1. NQO1, NAD(P)H quinone oxidoreductase 1. ZnPP, zinc protoporphyrin. NOX2, nitric oxide synthase 2. *TNF*, tumor necrosis factor. COX-2, cyclooxygenase-2. The data are expressed as means ± SEM (*n* = 3), with * indicating *p* < 0.05, ** indicating *p* < 0.01, and ns indicating *p* > 0.05. Samples originated from the same experiment and blots were conducted in parallel. The original images of Western Blot and immunofluorescence can be found in File S1.

As shown in Figure 4A–E, ZnPP pretreatment counteracted the meloxicam-induced rise in the mRNA expression of *NFE2L2*, *HMOX1*, and *NQO1*. It also reversed the suppression of proinflammatory genes (*TNF* and *NOX2*) in BEECs under oxidative stress. In contrast to the LPS group, in the LPS + ZnPP group, the expression of *HMOX1*, *NQO1*, and *TNF* decreased (*p* < 0.05), while *NOX2* expression increased (*p* < 0.01) and there was no significant change in *NFE2L2* expression. When meloxicam was incorporated into the LPS and ZnPP co-treatment, the *HMOX1* expression increased (*p* < 0.01), whereas the expression of *NFE2L2* and *NQO1* remained unchanged (*p* > 0.05).

Consistent with the expression patterns of antioxidant and proinflammatory genes, the effects that meloxicam promoted (*p* < 0.01), including the expression of key proteins of the Nrf2 pathway and suppression (*p* < 0.05) of COX-2 and nuclear P65 levels, were all abolished (*p* < 0.01) by ZnPP pretreatment (Figure 4F–L). ZnPP generally reduced the protein expression of NRF2, HO-1, and NQO1 in both the LPS vs. LPS + ZnPP and the LPS + MEL vs. LPS + MEL + ZnPP comparisons, with the levels approximate or even below those observed in the LPS group. Under the LPS and ZnPP co-treatment conditions, meloxicam mildly promoted (*p* < 0.05) HO-1 and NQO1 protein expression while inhibiting (*p* < 0.01) COX-2 and nuclear P65 levels. The original images can be found in File S1.

The immunofluorescence results confirmed the reduced (*p* < 0.01) nuclear NRF2 signal intensity induced by ZnPP under the co-treatment conditions with LPS and meloxicam (Figure 4M,N). In addition, meloxicam caused a slight increase (*p* < 0.05) in nuclear NRF2 abundance in the presence of ZnPP (LPS + ZnPP vs. LPS + MEL + ZnPP). No change (*p* > 0.05) was found in the nuclear signal intensity between the LPS and the LPS + ZnPP groups.

### 3.5. Nrf2 Inhibitor Abolished Meloxicam Antioxidation

**Figure 5:**
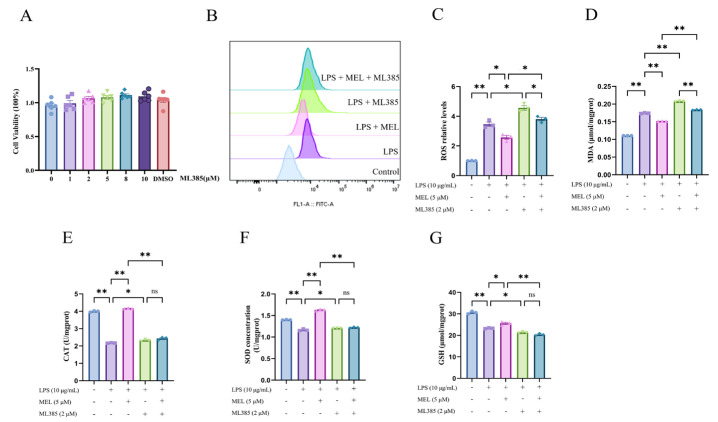
ML385 abolished the antioxidative effect of meloxicam in bovine endometrial epithelial cells stimulated with lipopolysaccharide. (**A**) Cell viability at 24 h was assessed in response to various concentrations of ML385. (**B**–**G**) Cells were first subjected to a 12 h pretreatment with ML385. Subsequently, the cells underwent a combined treatment with LPS and MEL for 12 h to determine the changes in ROS (**B**,**C**), MDA (**D**), CAT (**E**), SOD (**F**), and GSH (**G**). CAT, catalase. GSH, glutathione. LPS, lipopolysaccharide. MDA, malondialdehyde. MEL, meloxicam. ROS, reactive oxygen species. SOD, superoxide dismutase. The data are expressed as means ± SEM (*n* = 3 or *n* = 6), with * indicating *p* < 0.05, ** indicating *p* < 0.01, and ns indicating *p* > 0.05.

As illustrated in Figure 5A, ML385 at concentrations of 1, 2, 5, 8, and 10 μM had no significant impact on BEEC viability. When compared to the LPS group, the cells in the LPS + ML385 group exhibited higher levels of ROS and MDA (*p* < 0.05). Similarly, the cells in the LPS + MEL + ML385 group had higher ROS and MDA levels relative to the LPS + MEL group (*p* < 0.05). However, the inclusion of meloxicam decreased the ROS and MDA levels in comparison to the LPS + ML385 group (*p* < 0.05). Analogous to the previously mentioned findings, meloxicam supplementation upregulated the levels of antioxidant markers relative to the LPS group (*p* < 0.05, Figure 5E–G). This antioxidant effect of meloxicam was reversed (*p* < 0.01) by ML385 in LPS-stimulated BEECs. In comparison to the LPS group, supplementation with ML385 led to elevated concentrations of CAT and SOD, yet a decreased GSH concentration (all *p* < 0.05). There was no significant difference in the concentrations of CAT, SOD, and GSH between the LPS + ML385 and the LPS + MEL + ML385 groups.

### 3.6. Nrf2 Inhibitor Reversed Meloxicam-Induced Nrf2 Activation

**Figure 6:**
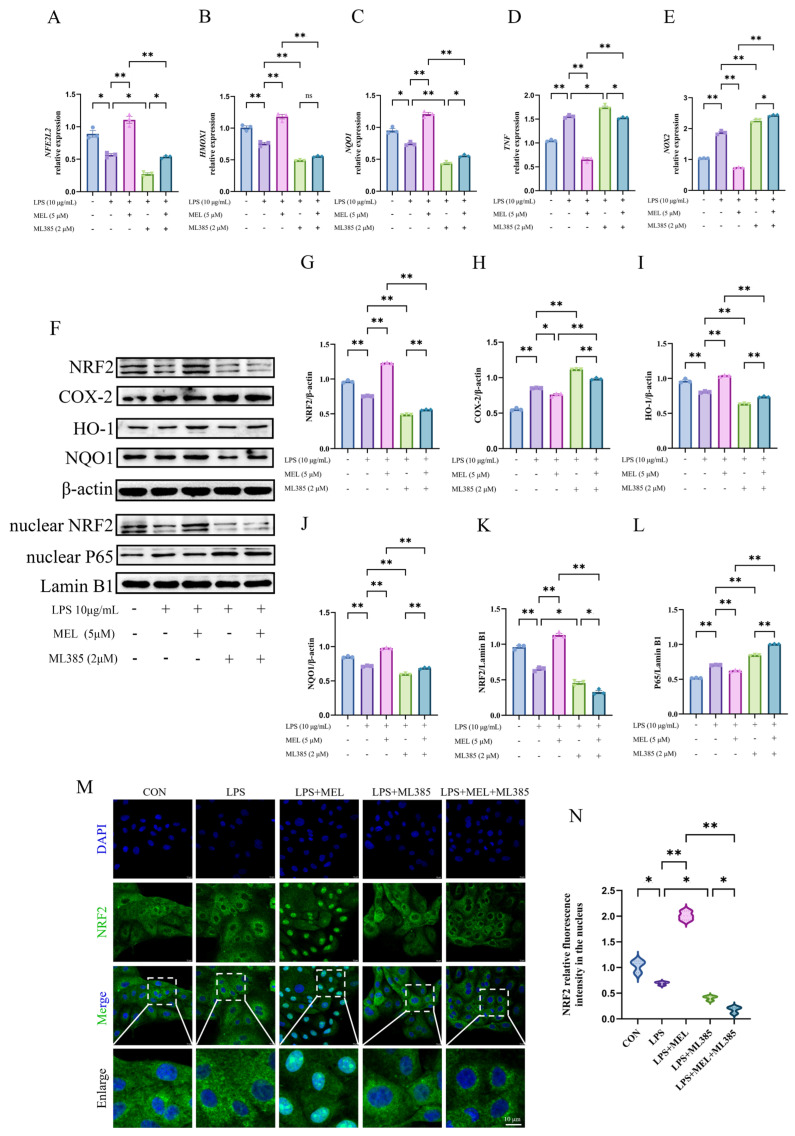
ML385 reversed the meloxicam-induced Nrf2 activation in bovine endometrial epithelial cells. The cells were first subjected to a 12 h pretreatment with the Nrf2-specific inhibitor ML385. Subsequently, the cells underwent a combined treatment with LPS and MEL for another 12 h. Quantitative PCR was then employed to detect alterations in the relative mRNA levels of *NFE2L2* (**A**), *HMOX1* (**B**), *NQO1* (**C**), *TNF* (**D**), and *NOX2* (**E**). Western blot (**F**) analysis, using β-actin as the internal control, was carried out to determine the relative protein levels of total NRF2 (**G**), COX-2 (**H**), HO-1 (**I**), NQO1 (**J**), nuclear NRF2 (**K**), and nuclear P65 (**L**). Immunofluorescence, with a scale bar of 10 μM, was utilized to visualize (**M**) and quantify (**N**) the amount of nuclear NRF2. LPS, lipopolysaccharide. MEL, meloxicam. NRF2/*NFE2L2*, nuclear factor-erythroid 2 related factor 2. HO-1/*HMOX1*, heme oxygenase 1. NQO1, NAD(P)H quinone oxidoreductase 1. NOX2, nitric oxide synthase 2. *TNF*, tumor necrosis factor. COX-2, cyclooxygenase-2. The data are expressed as means ± SEM (*n* = 3), with * indicating *p* < 0.05, ** indicating *p* < 0.01, and ns indicating *p* > 0.05. Samples originated from the same experiment and blots were conducted in parallel. The original images of Western Blot and immunofluorescence can be found in File S1.

As shown in Figure 6A–L, pretreatment with ML385 reversed (*p* < 0.01) the meloxicam-induced expression (*p* < 0.01) of genes (*NFE2L2*, *HMOX1*, and *NQO1*) and proteins (NRF2, HO-1, and NQO1) in Nrf2 pathway, as well as the meloxicam-induced suppression (*p* < 0.01) of proinflammatory genes (*TNF* and *NOX2*) and proteins (COX-2 and nuclear P65) in BEECs with oxidative stress. In addition, ML385 further reduced (*p* < 0.05) the already inhibited expression of these antioxidant genes and proteins under LPS stimulation, while concurrently exacerbating (*p* < 0.05) the expression of the proinflammatory genes and proteins. In comparison to the co-treatment with LPS and ML385, there was generally a mild rise in the antioxidant gene and protein expression (except nuclear NRF2) and a decrease in proinflammatory gene and protein expression (except *NOX2* and nuclear P65) in the cells of the LPS + MEL + ML385 group. According to the immunofluorescent result in Figure 6M,N, ML385 prevented (*p* < 0.05) the meloxicam-induced NRF2 translocation to the nucleus in LPS-stimulated BEECs. A further reduction (*p* < 0.05) in nuclear NRF2 protein was observed in response to meloxicam treatment in BEECs cotreated with LPS and ML385. The original images can be found in File S1.

## 4. Discussion

Previously, we demonstrated that meloxicam possesses anti-inflammatory and antioxidant properties and is capable of activating the Nrf2 pathway [13]. Building upon these earlier findings, we incorporated a positive control (NAC) and specific pathway inhibitors (ZnPP and ML385) to delve deeper into the role of the Nrf2/HO-1 pathway in the antioxidant effects exerted by meloxicam.

LPS is able to induce oxidative damage in bovine endometrial epithelial cells, causing a marked increase in ROS content and a decrease in antioxidant enzyme activities [26]. ROS targets the unsaturated fatty acids within the cell membrane, triggering a chain reaction that leads to MDA generation. This process reflects the extent of cellular damage induced by oxidative stress. SOD serves as the first line of defense in the antioxidant system, catalyzing the conversion of superoxide anions into hydrogen peroxide. CAT is responsible for decomposing hydrogen peroxide into water and oxygen. Meanwhile, GSH acts as a crucial intracellular antioxidant. It not only directly eliminates ROS and MDA but also contributes to preserving the activity of other antioxidant enzymes. In line with this, our findings showed a rise in ROS content alongside a reduction in the levels of CAT, SOD, and GSH. Nrf2 signaling serves as the primary defense mechanism against oxidative stress, during which the Nrf2 protein undergoes nuclear translocation and binds to the antioxidant response elements to initiate antioxidant gene transcription, including glutathione S-transferases, SOD, and glutathione peroxidases. In vitro studies have shown that different concentrations of LPS elicited varying effects on the Nrf2 pathway. For instance, when a BEEC was exposed to 1 μg/mL LPS for 90 min, the expression of NRF2, HO-1, and NQO1 decreased [13]. Likewise, treating a bovine endometrial epithelial cell with 1 μg/mL LPS for 3 h led to a decline in the mRNA expression of *HMOX1* and *NQO1*; however, nuclear NRF2 protein was not assayed [27]. After treating bovine endometrial epithelial cells with 1 μg/mL LPS for 12 h, the protein expressions of NRF2, HO-1, and NQO1 in the cells decreased [28]. Conversely, a 24 h treatment with 10 μg/mL LPS elevated the mRNA levels of *NFE2L2*, *HMOX1*, and *NQO1*, along with the nuclear NRF2 protein level, in bovine endometrial epithelial cells [29]. Our findings revealed that a 12 h exposure to 10 μg/mL LPS repressed the expression of Nrf2 pathway-associated proteins and genes, which is different from previous reports. These studies suggested that differences in LPS concentration and duration of action have distinct effects on the Nrf2 signaling pathway. The underlying reason for this may be that under short-term LPS treatment (90 min to 12 h), the Nrf2/KEAP1 pathway is suppressed, thereby inhibiting Nrf2 expression [13]. However, during prolonged LPS exposure (such as 24 h), LPS may continually activate Nrf2 expression through mechanisms related to endoplasmic reticulum stress [30].

The thiol group of NAC can directly neutralize ROS [31]. As a precursor of glutathione (GSH), NAC promotes GSH synthesis and restores cellular antioxidant capacity [32,33]. Similarly, according to the current findings, NAC reduced ROS and MDA accumulation and enhanced the levels of CAT, SOD, and GSH.

Consistent with our previous report [13], the current results indicated that meloxicam alleviated LPS-induced oxidative stress in BEECs. Interestingly, while the addition of meloxicam to LPS and NAC co-treatment did not further alter ROS and MDA levels, supplementing the LPS and meloxicam combination with NAC led to a further reduction in cellular ROS and MDA. It seems that the antioxidant effect of meloxicam is weaker than that of NAC and only partially scavenges ROS, with the specific mechanism yet to be elucidated.

The selective inhibition of the prostaglandin-endoperoxide synthase 2 gene, which encodes COX-2, by meloxicam has been observed in BEECs [13,34]. The current result further verified the suppressive effect of meloxicam on COX-2 at the protein level. When used in combination, meloxicam and NAC produced a more pronounced anti-inflammatory effect than either agent alone, a phenomenon consistent with the synergistic mechanisms of ROS and inflammatory factors/mediators in the inflammatory cascade. Notably, the COX-2 protein level was reduced in the LPS + MEL + NAC group compared to LPS + NAC but remained unchanged relative to the LPS + MEL group. Based on the observed modulation of proinflammatory gene expression and oxidative stress markers, we propose that meloxicam’s addition to LPS + NAC co-treatment primarily exerts its anti-inflammatory effects through COX-2 inhibition, hence resulting in no significant changes in oxidative stress markers.

Nrf2 plays a crucial role in oxidative stress and inflammatory diseases. Meloxicam has been reported to relieve oxidative stress by inhibiting NADPH oxidase (NOX1/NOX4) and simultaneously activating the Nrf2 pathway and upregulating the expression of downstream antioxidant enzymes, thereby exerting neuroprotective effects in experimental models of central nervous system diseases [19]. In a chronic aluminum overload model in rats, meloxicam alleviated hepatic oxidative damage by upregulating Nrf2 expression [35]. The current results demonstrated that meloxicam treatment upregulated both the gene and protein expression of key components in the Nrf2 pathway and downregulated the nuclear P65 level in BEECs, indicating Nrf2 activation and NF-κB inhibition. Notably, the total NRF2 and nuclear NRF2 protein levels in the LPS + MEL + NAC group were higher compared to those in the LPS + NAC and LPS + MEL groups, whereas the protein and gene expressions of HO-1 and NQO1 either decreased or remained unchanged and were accompanied by a reduction in the relative expression of *NFE2L2* mRNA. The inconsistency between the expression of the Nrf2 gene and its protein, as well as the discrepancies observed between Nrf2 and its downstream targets, may be attributed to the asynchronous temporal changes in expression and the regulatory effects of NAC on the Nrf2 pathway. NAC can inhibit the activation of extracellular regulated protein kinases (ERK) by scavenging ROS. The ERK pathway, in turn, promotes the separation of Nrf2 from its inhibitor through phosphorylation, thereby enhancing Nrf2′s transcriptional activity. Consequently, with the suppression of ERK, even after Nrf2 moves into the nucleus, the transcription of downstream Nrf2 pathway genes and proteins may still be constrained [36].

Subsequently, we employed ZnPP, a HO-1-specific inhibitor, to verify the role of HO-1 in meloxicam’s antioxidation. ZnPP is a compound formed by the chelation of protoporphyrin IX with zinc ions that primarily affects HO-1’s antioxidant function by competitively inhibiting HO-1’s activity [37]. Based on the literature and the observed result that cell viability was unaffected by ZnPP concentrations ranging from 1 to 10 μM, one μM was selected for this experiment [38]. Our pre-experiments confirmed that ZnPP monotreatment inhibits HO-1 protein expression (Supplementary Materials Figure S1). Notably, this inhibition persisted in the presence of LPS, as ZnPP treatment downregulated HO-1 expression at both the transcriptional and translational levels, which is consistent with the established mechanism of ZnPP action.

HO-1 catalyzes heme degradation, yielding iron ions, CO, and bilirubin. The iron ions facilitate the synthesis of ferritin. CO exhibits anti-apoptotic, anti-proliferative, and anti-inflammatory effects, while bilirubin acts as both an antioxidant and an anti-inflammatory factor [39]. In a melatonin-treated murine model of sepsis-induced acute kidney injury, ZnPP administration elevated MDA levels while reducing GSH content [40]. This finding is analogous to our results, which revealed that ZnPP pretreatment exacerbated LPS-induced inflammatory responses and oxidative stress. During LPS-induced oxidative stress in BEECs, the depletion of HO-1-associated antioxidative components led to a marked increase in oxidative stress biomarkers, thereby impairing the antioxidant activity of Nrf2. Notably, pretreatment with ZnPP significantly attenuated meloxicam’s antioxidant and anti-inflammatory effects in LPS-stimulated conditions, suggesting that HO-1 is involved in mediating meloxicam’s antioxidant actions. In the presence of ZnPP, we found that meloxicam only showed a mild antioxidant effect on BEECs (LPS-ZnPP vs. LPS-MEL-ZnPP comparison). The above results indicated that under ZnPP pretreatment, meloxicam was unable to fully activate the Nrf2 pathway, which is probably related to HO-1 inhibition. Interestingly, ZnPP has been found to inhibit the downregulation of p65 and c-Jun expression and the activation of NQO1 induced by p-hydroxybenzaldehyde in RAW264.7 cells without affecting the level of Nrf2 [41]. However, our findings demonstrated a reduction in both the mRNA and protein levels of Nrf2 subsequent to the inhibition of HO-1. Molecular biology studies have confirmed that HO-1 is downstream of Nrf2 [42]. HO-1 itself possesses antioxidant properties, and its inhibition exacerbates cellular oxidative stress, subsequently triggering the activation of pro-inflammatory pathways such as the NF-κB pathway [43]. Pharmacological and genetic investigations have demonstrated functional crosstalk between the Nrf2 and NF-κB signaling, where NF-κB activation downregulates Nrf2 activity and expression [44].

ML385 is a specific Nrf2 inhibitor that functions by binding to the Neh1 domain (bZIP region) of Nrf2, thereby blocking its heterodimerization with the MAFG protein and suppressing the expression of downstream antioxidant genes [45]. In this study, cells were treated with various concentrations of ML385 (1 to 10 μM), none of which affected cell viability; 2 μM was selected for experimental use based on the literature [46]. Our preliminary experiments validated that treatment with ML385 alone suppresses NRF2 expression (Supplementary Materials Figure S2). Under inflammatory conditions, ML385 treatment significantly downregulated Nrf2 and its downstream target gene/protein expression, further confirming its suppressive effect on the Nrf2 pathway.

Here, we aimed to elucidate the role of Nrf2 in meloxicam-mediated antioxidant protection by blocking Nrf2 signaling. Our results demonstrated that ML385 reversed meloxicam-induced activation of the Nrf2 pathway and enhancement of antioxidant enzyme activities. Specifically, ML385 pretreatment abrogated meloxicam’s inhibitory effects on ROS/MDA accumulation and inflammatory markers (TNF, COX-2), as well as its upregulatory effects on antioxidant markers (SOD, GSH) and Nrf2 pathway activation, suggesting the pivotal role of Nrf2 in meloxicam’s cytoprotective effect.

Notably, while the LPS + ML385 group exhibited exacerbated oxidative stress and inflammation, meloxicam co-treatment partially mitigated these effects and restored Nrf2 downstream gene expression without increasing nuclear NRF2 translocation. This contrasts with ML385’s ability to block Songorine-induced NRF2 nuclear accumulation [47] and its suppression of lentinan-mediated nuclear NRF2 expression and downstream antioxidant gene activation [46]. Collectively, the current observations suggested that meloxicam partially rescues antioxidant defenses through Nrf2-independent mechanisms. It has been demonstrated that, in an LPS-induced endometritis model of cows, the activation of Nrf2 by the flavonoid fisetin promotes the expression of antioxidant enzymes such as HO-1 and inhibits the accumulation of ROS, thereby blocking the activation of NF-κB and indirectly suppressing COX-2 expression. This indicates that Nrf2 can negatively regulate COX-2 by antagonizing oxidative stress and inflammatory signaling [29]. Research has also indicated that the metabolic products of COX-2 may exert anti-inflammatory effects by activating Nrf2/antioxidant response element signaling. For instance, COX-2-dependent oxidative metabolites (such as prostaglandin derivatives) activate Nrf2, thereby enhancing cellular antioxidant capacity and forming a negative feedback regulatory loop [30]. The aforementioned interactions between Nrf2 and COX-2 imply that COX-2 also plays a role in the oxidative stress process, which was an aspect we had not explored in our current study. Finally, our results predominantly rely on in vitro cell experiments using pathway inhibitors and lack validation in live animal models (such as dairy cows). In future research, we will further address these limitations by employing gene-editing techniques and conducting animal trials.

## 5. Conclusions

In conclusion, meloxicam alleviates the inflammatory response and oxidative stress in lipopolysaccharide-stimulated bovine endometrial epithelial cells through the Nrf2/HO-1 pathway.

## Figures and Tables

**Table 1:** The target genes and primer sequences.

Gene Name	Primer Sequence (5′ → 3′)	Length (bp)	NCBI Number
*ACTB*	F: CATCACCATCGGCAATGAGC R: AGCACCGTGTTGGCGTAGAG	156	NM_173979.3
*NFE2L2*	F: CCCAGTCTTCACTGCTCCTC R: TCAGCCAGCTTGTCATTTTG	165	NM_001011678.2
*HMOX1*	F: GGCAGCAAGGTGCAAGA R: GAAGGAAGCCAGCCAAGAG	221	NM_001014912.1
*NQO1*	F: AACCAACAGACCAGCCAATC R: CACAGTGACCTCCCATCCTT	154	NM_001034535.1
*TNF*	F: GGACACCCAGAATGTGAGGG R: GGAGAGTTGAAGTCCACGCA	102	NM_173966.3
*NOX2*	F: CTCAGCTACAACATCTGCCTCACT R: CTGTGATTACATCTTTCTCCTCGTCAT	91	NM_174035.4

## Data Availability

The data presented in this study are available in the article.

## Supplementary Materials

The following supporting information can be downloaded at: https://www.mdpi.com/article/10.3390/vetsci12060579/s1, Figure S1: ZnPP monotreatment downregulated HO-1 expression in BEEC; Figure S2: ML385 monotreatment reduced NRF2 expression in BEEC; File S1: Original images for Figure 2, Figure 4 and Figure 6.

## Abbreviations

Here are the main abbreviations used in the current manuscript:

BEEC	Bovine endometrial epithelial cell
COX-2	Cyclooxygenase-2
CAT	Catalase
ERK	Extracellular regulated protein kinases
GSH	Glutathione
*HMOX1*/HO-1	Bos taurus heme oxygenase 1
LPS	Lipopolysaccharide
MDA	Malondialdehyde
NSAID	Non-steroidal anti-inflammatory drugs
NF-κB	Nuclear factor kappa-B
*NFE2L2*/Nrf2	Nuclear factor erythroid 2-related factor 2
NQO1	NAD(P)H quinone oxidoreductase 1
*NOX2*	NADPH oxidase 2
NAC	N-acetylcysteine
ROS	Reactive oxygen species
SOD	Superoxide dismutase
*TNF*	Tumor necrosis factor
ZnPP	Zinc protoporphyrin
