# Supplementary material for: Meloxicam Alleviates Oxidative Stress Through Nrf2/HO-1 Activation in Bovine Endometrial Epithelial Cells
Source: Vet Sci. 2025 Jun 12;12(6):579. doi: 10.3390/vetsci12060579 (PMC12197372; doi:10.3390/vetsci12060579)
Supplement: Supplementary file 1 [file vetsci-12-00579-s001.zip › vetsci-3652526-Figures S1 S2.pdf]

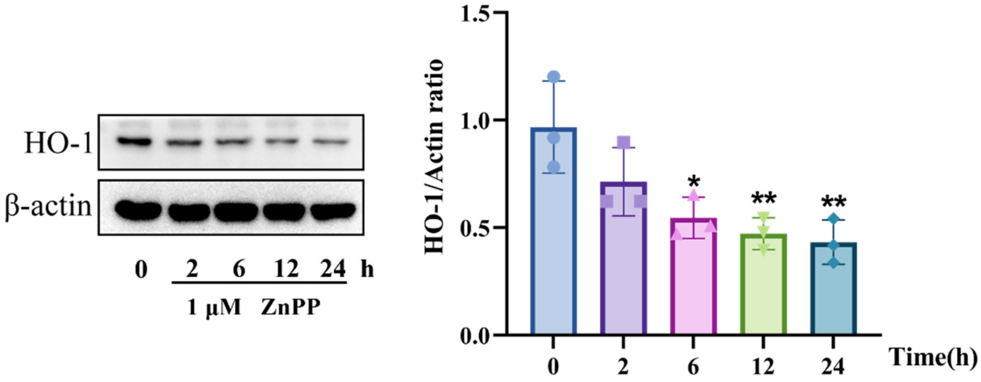

Figure S1. ZnPP monotreatment downregulated HO-1 expression in BEEC. ZnPP reduced the expression level of HO-1 protein in bovine endometrial epithelial cells. The cells were stimulated with 1 μM ZnPP for 0, 2, 6, 12, and 24 h to detect changes in HO-1 protein expression. HO-1, heme oxygenase 1. ZnPP, zinc protoporphyrin. Data were presented as means ± SEM (n = 3). *P < 0.05, **P < 0.01. Samples were derived from the same experiment and blots were processed in parallel.

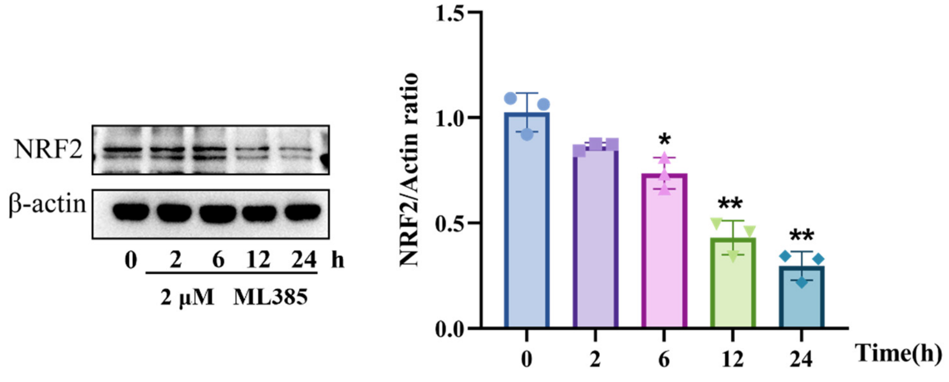

Figure S2. ML385 monotreatment reduced NRF2 expression in BEEC. ML385 decreased the expression level of NRF2 protein in bovine endometrial epithelial cells. The cells were treated with 2 μM ML385 for 0, 2, 6, 12, and 24 h to detect changes in NRF2 protein expression. NRF2, nuclear factor-erythroid 2 related factor 2. Data were presented as means ± SEM (n = 3), *P <

0.05, $^{**}P < 0.01$. Samples were derived from the same experiment and blots were processed in parallel.
