# Supplementary figures and images for: Meloxicam Alleviates Oxidative Stress Through Nrf2/HO-1 Activation in Bovine Endometrial Epithelial Cells
Source: Vet Sci. 2025 Jun 12;12(6):579. doi: 10.3390/vetsci12060579 (PMC12197372; doi:10.3390/vetsci12060579)

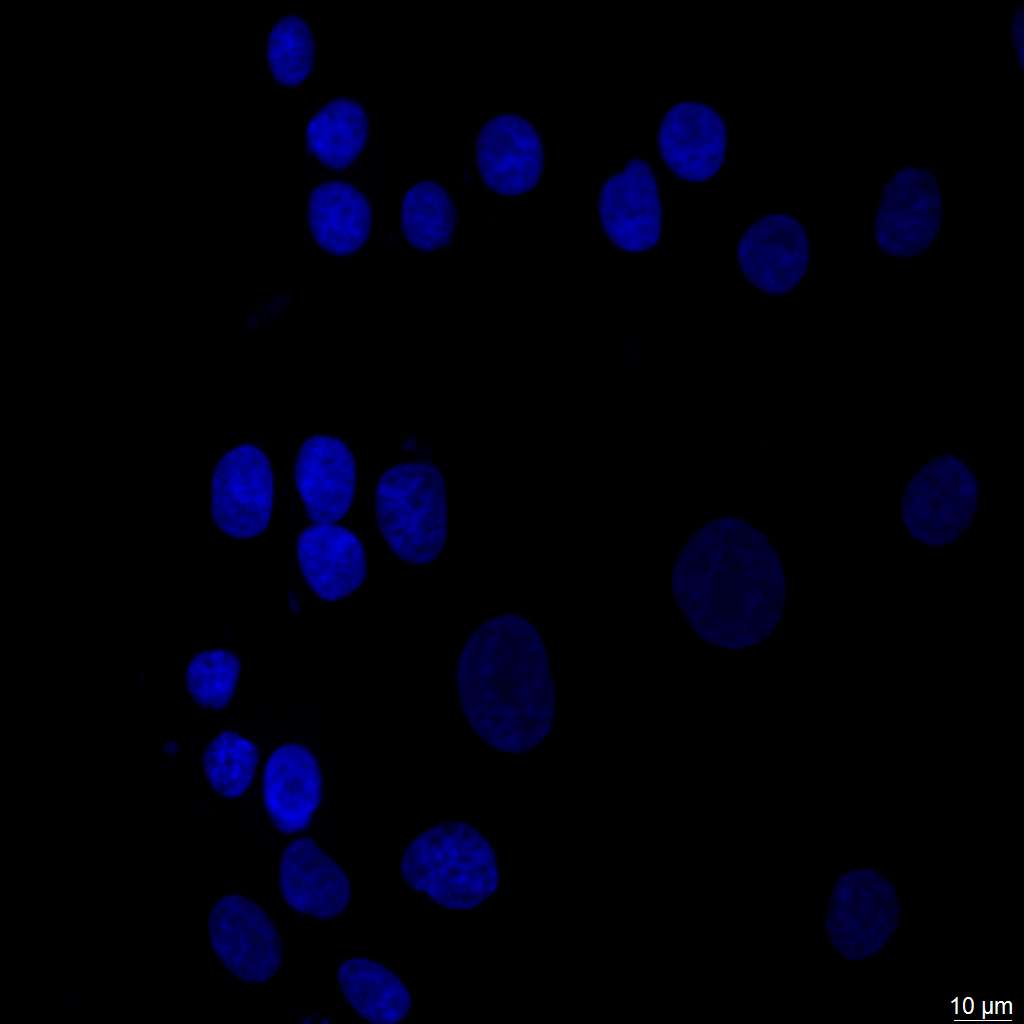

Supplement: Supplementary file 1 [file vetsci-12-00579-s001.zip › File S1/Fig2/IF/C/24.11.8Nrf2_24_ch00_SV.tif]

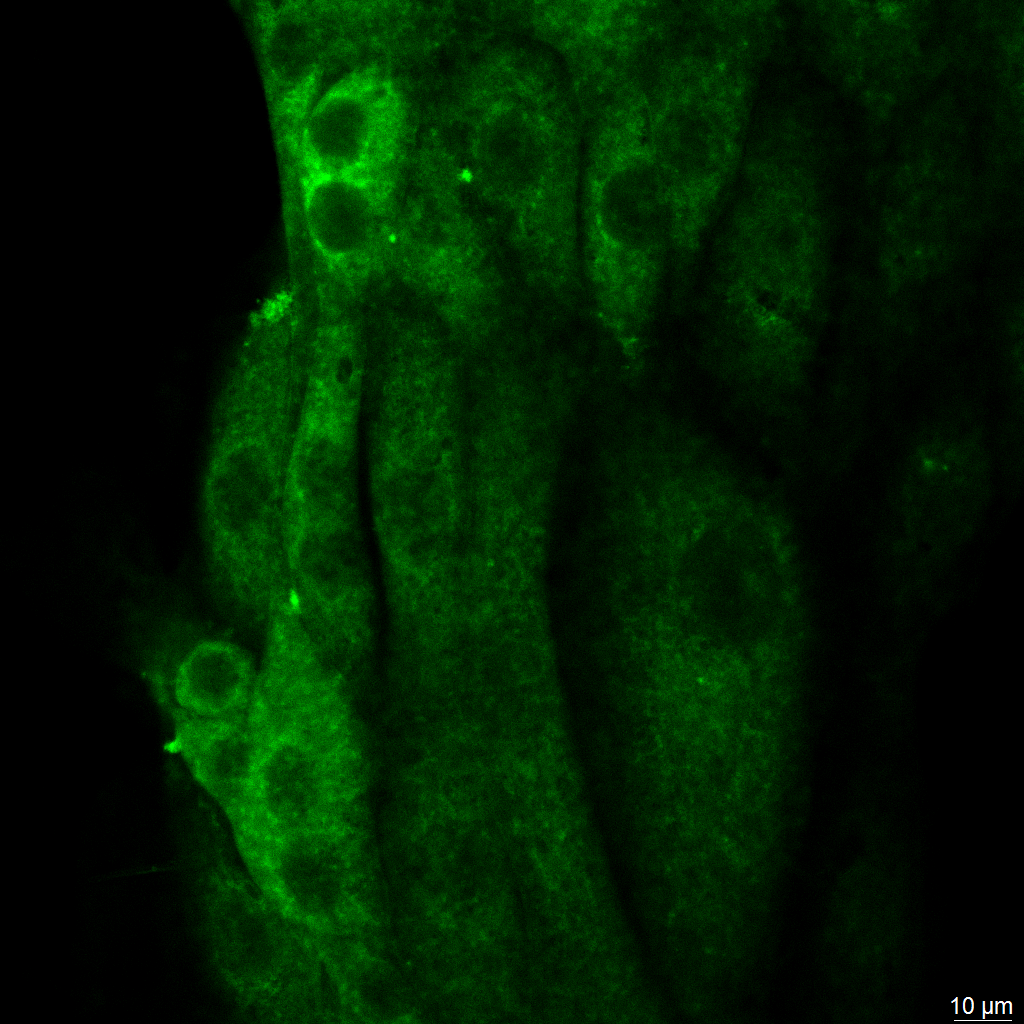

Supplement: Supplementary file 1 [file vetsci-12-00579-s001.zip › File S1/Fig2/IF/C/24.11.8Nrf2_24_ch01_SV.tif]

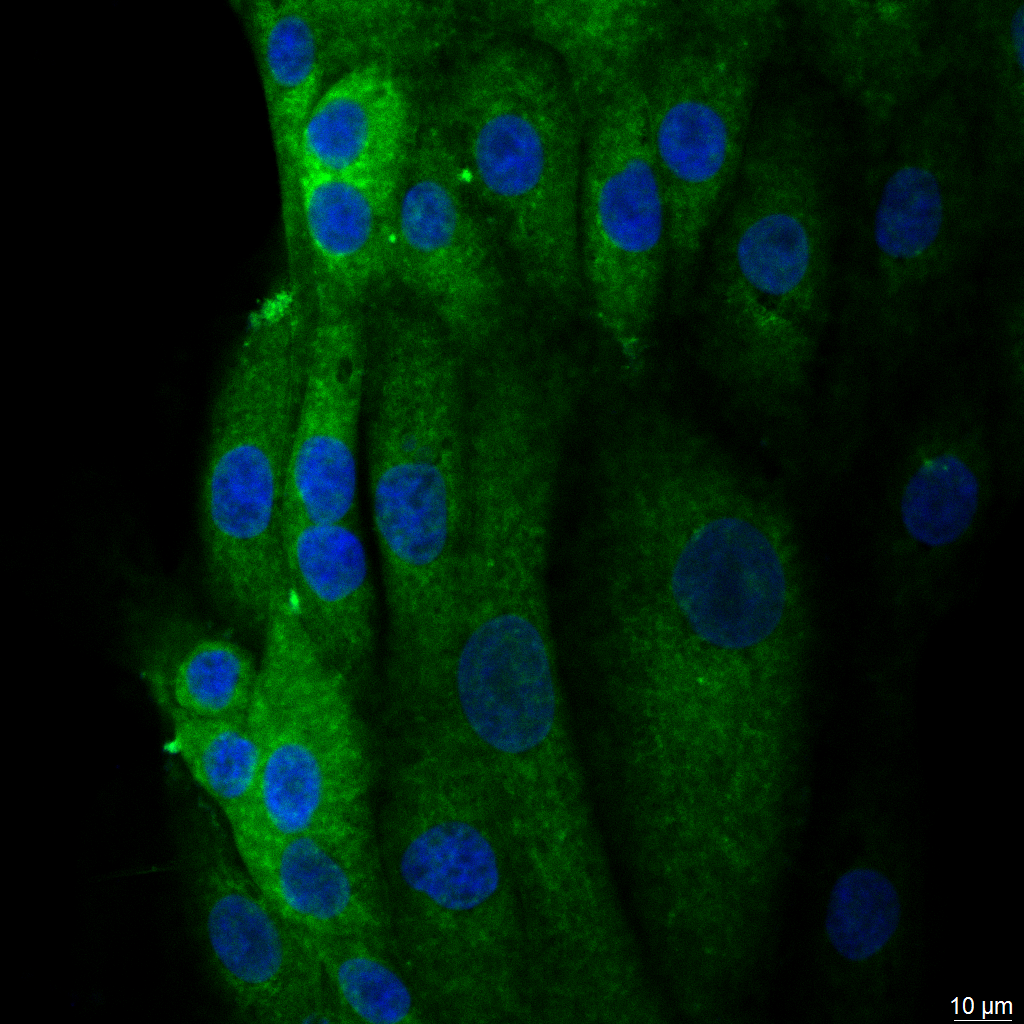

Supplement: Supplementary file 1 [file vetsci-12-00579-s001.zip › File S1/Fig2/IF/C/24.11.8Nrf2_24_overlay.tif]

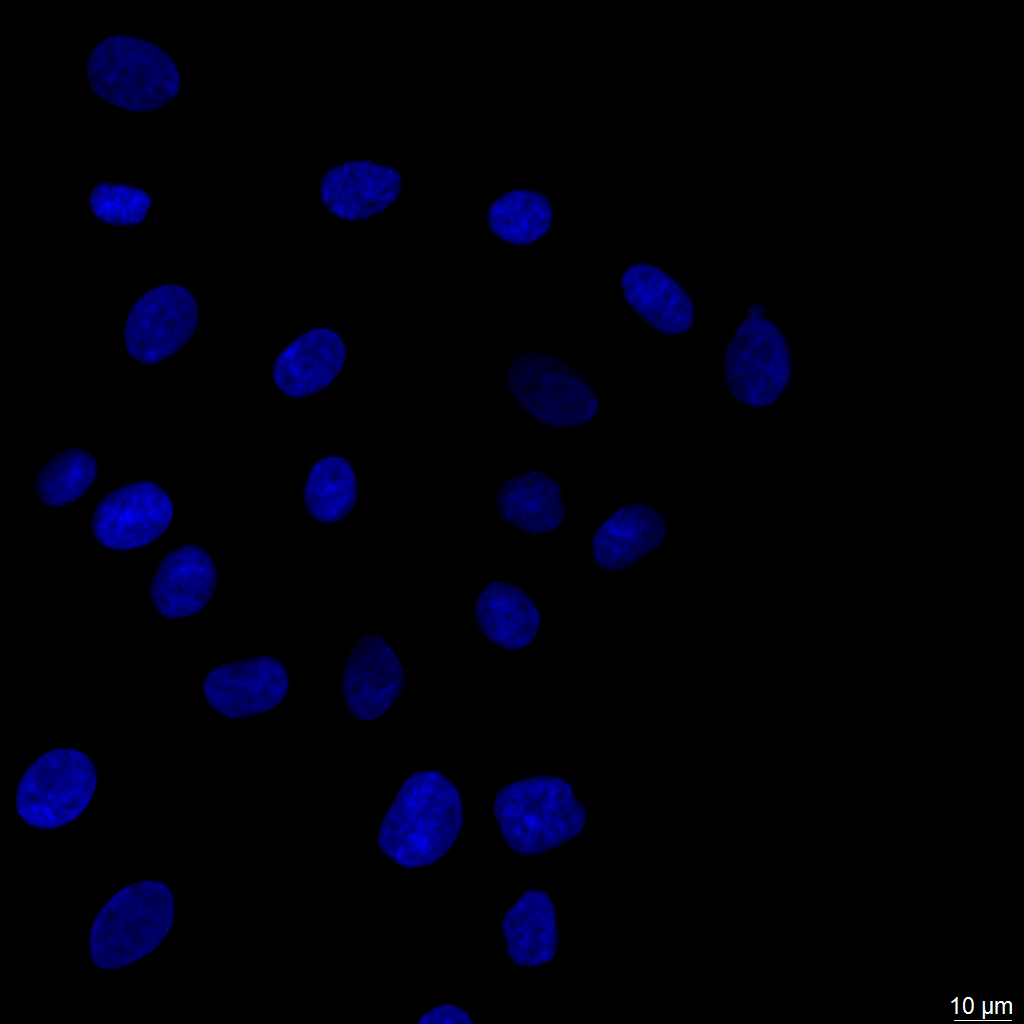

Supplement: Supplementary file 1 [file vetsci-12-00579-s001.zip › File S1/Fig2/IF/LPS+MEL+NAC/2024.11.8 Nrf2_10_ch00_SV.tif]

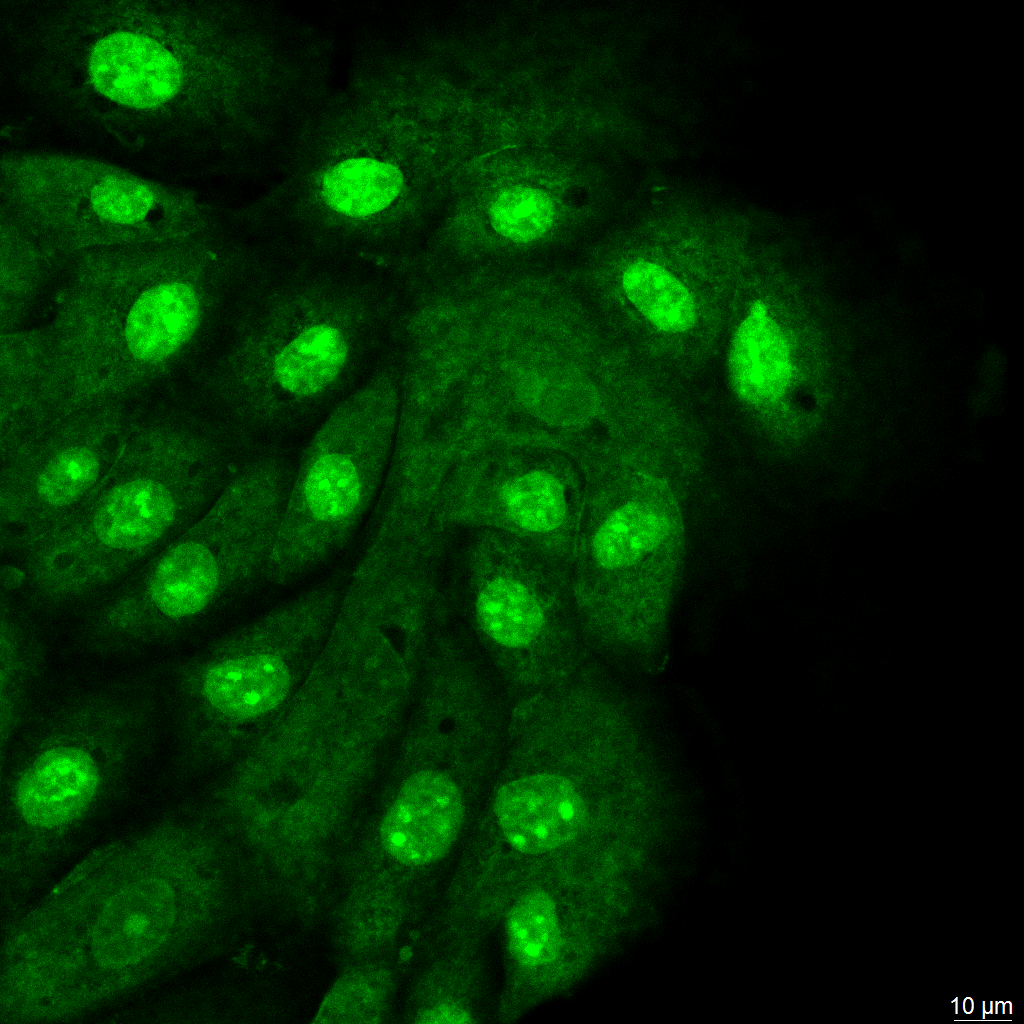

Supplement: Supplementary file 1 [file vetsci-12-00579-s001.zip › File S1/Fig2/IF/LPS+MEL+NAC/2024.11.8 Nrf2_10_ch01_SV.tif]

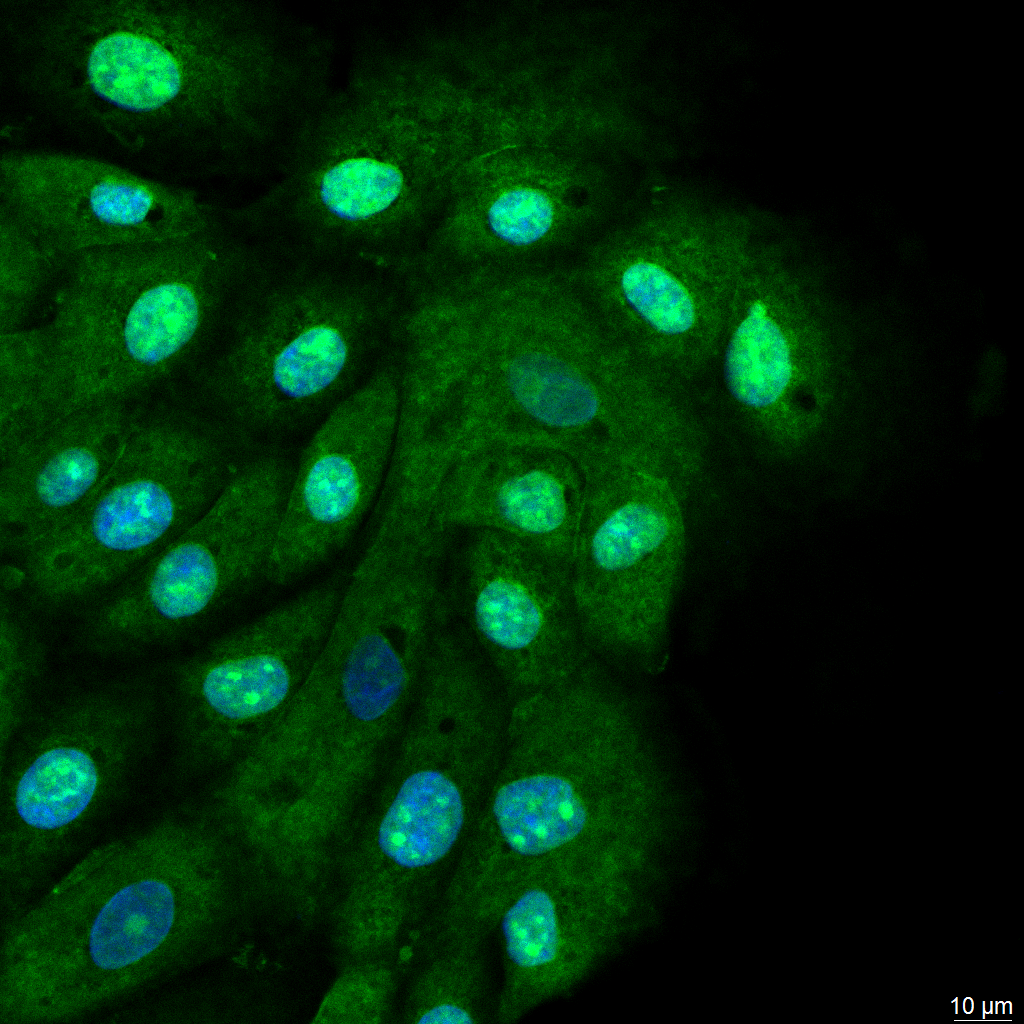

Supplement: Supplementary file 1 [file vetsci-12-00579-s001.zip › File S1/Fig2/IF/LPS+MEL+NAC/2024.11.8 Nrf2_10_overlay.tif]

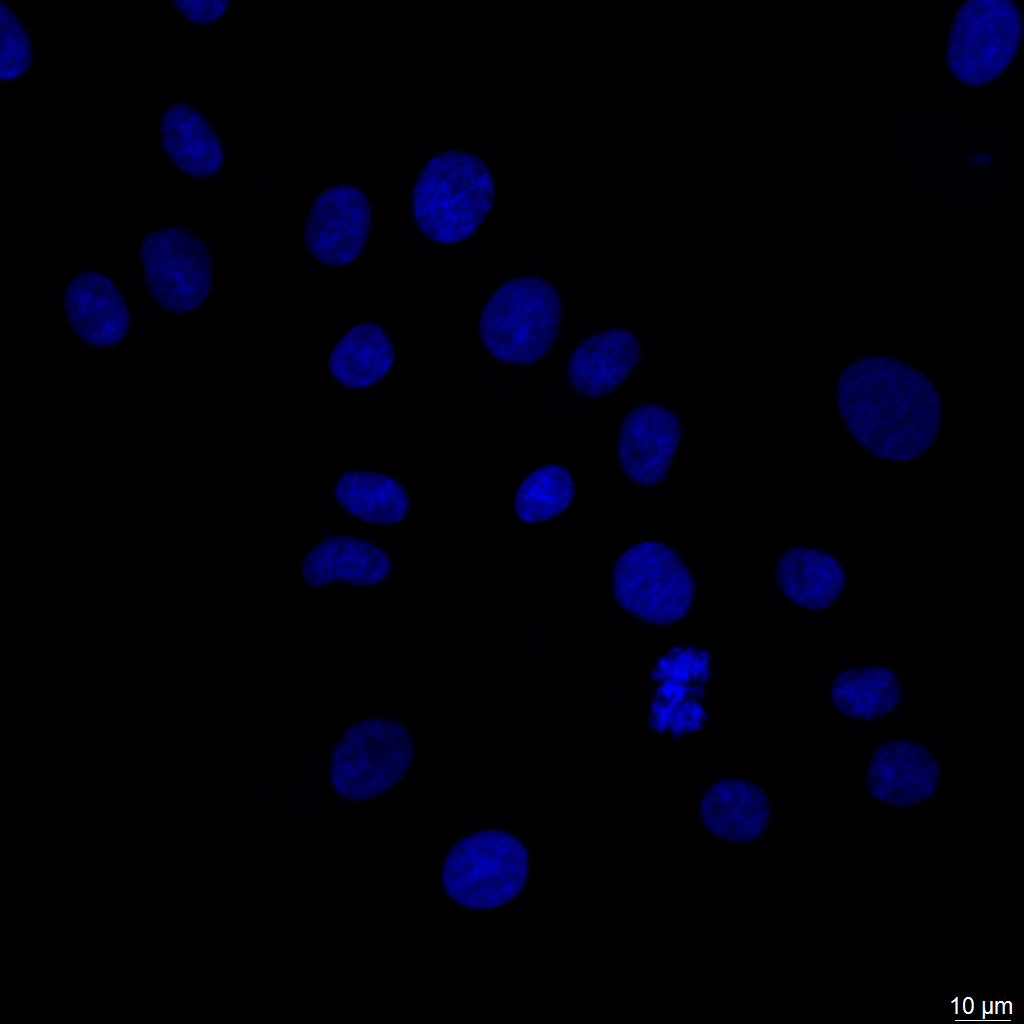

Supplement: Supplementary file 1 [file vetsci-12-00579-s001.zip › File S1/Fig2/IF/LPS+MEL/shaoruhe 20_53_ch00_SV.tif]

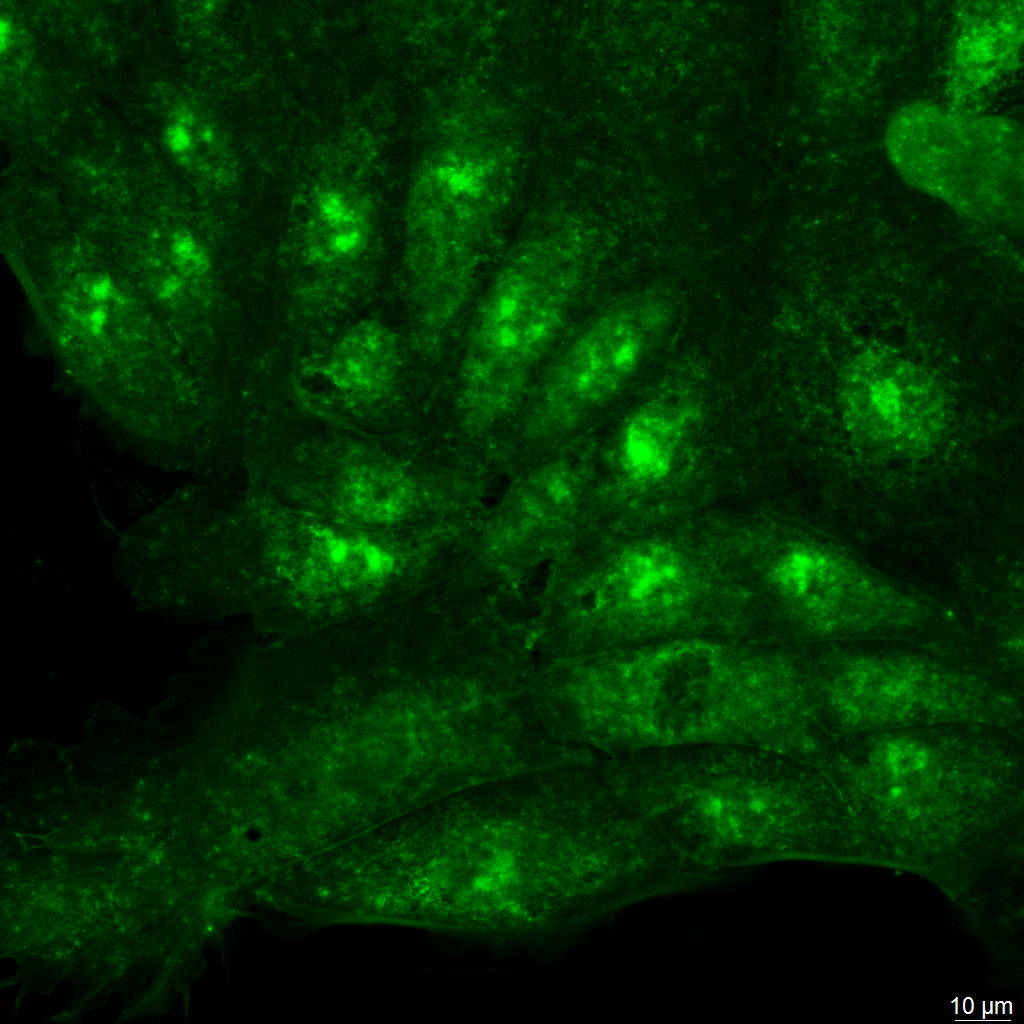

Supplement: Supplementary file 1 [file vetsci-12-00579-s001.zip › File S1/Fig2/IF/LPS+MEL/shaoruhe 20_53_ch01_SV.tif]

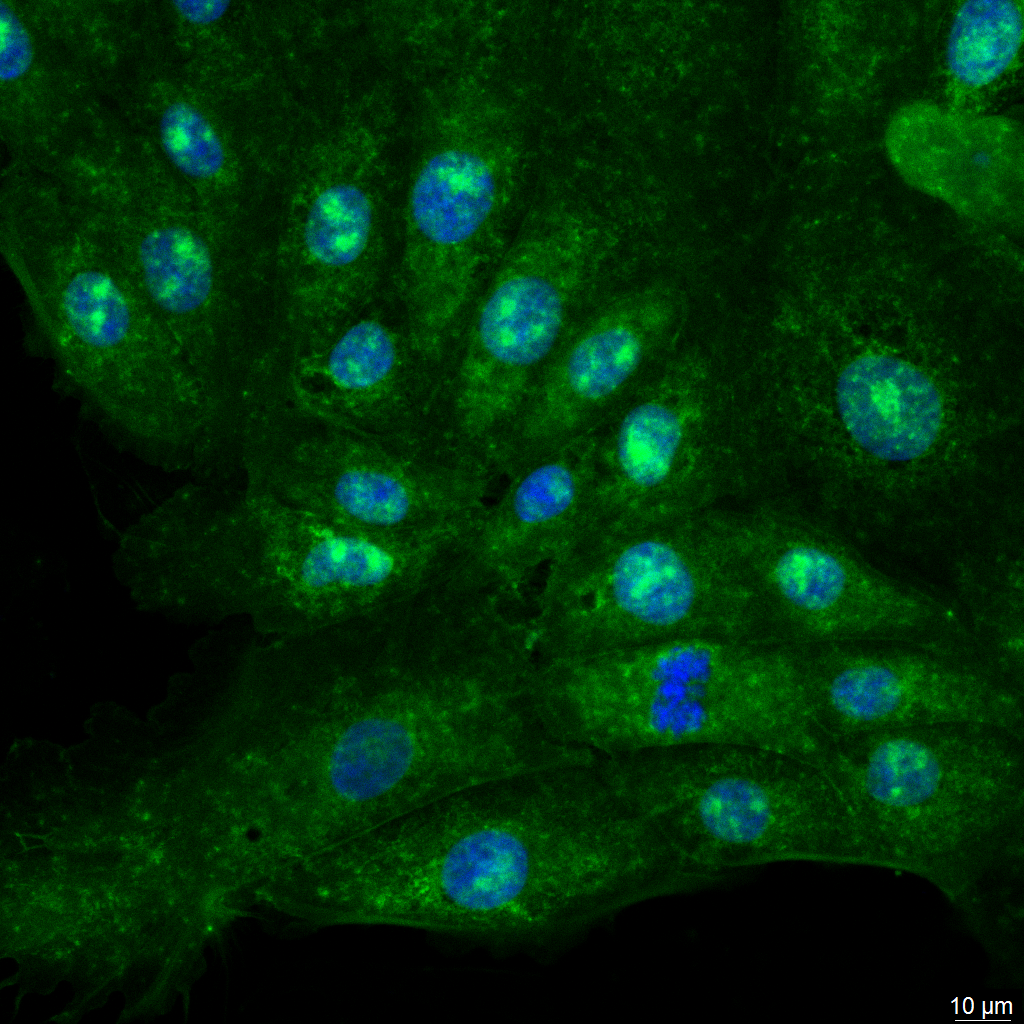

Supplement: Supplementary file 1 [file vetsci-12-00579-s001.zip › File S1/Fig2/IF/LPS+MEL/shaoruhe 20_53_overlay.tif]

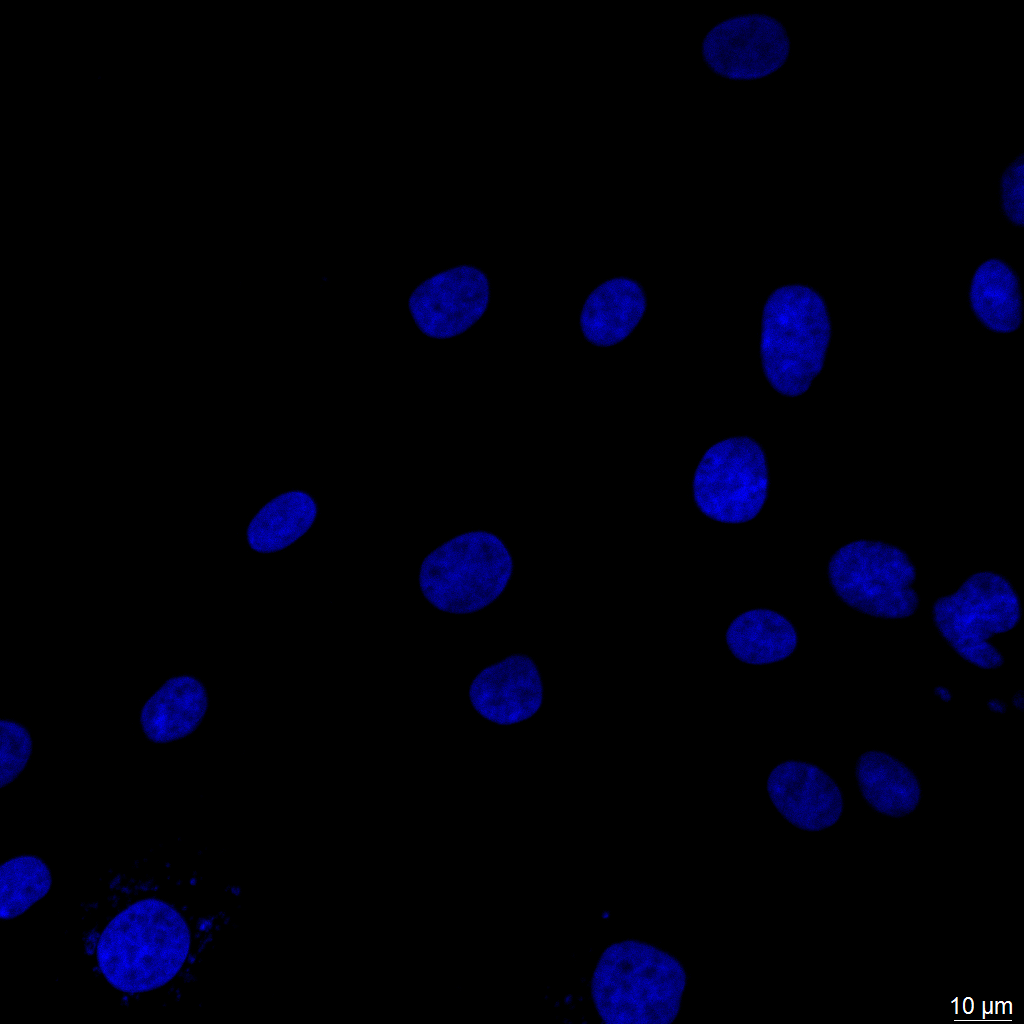

Supplement: Supplementary file 1 [file vetsci-12-00579-s001.zip › File S1/Fig2/IF/LPS+NAC/2024.11.8 Nrf2_11_ch00_SV.tif]

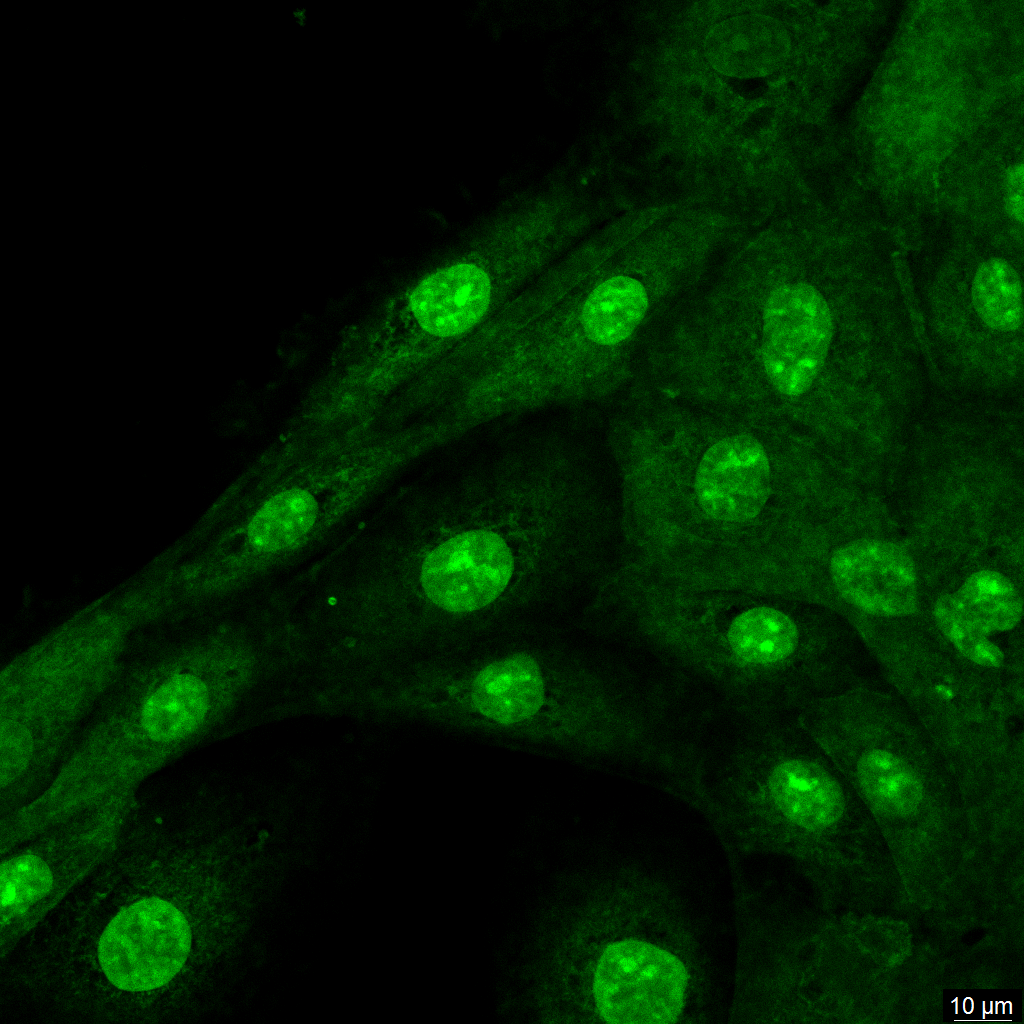

Supplement: Supplementary file 1 [file vetsci-12-00579-s001.zip › File S1/Fig2/IF/LPS+NAC/2024.11.8 Nrf2_11_ch01_SV.tif]

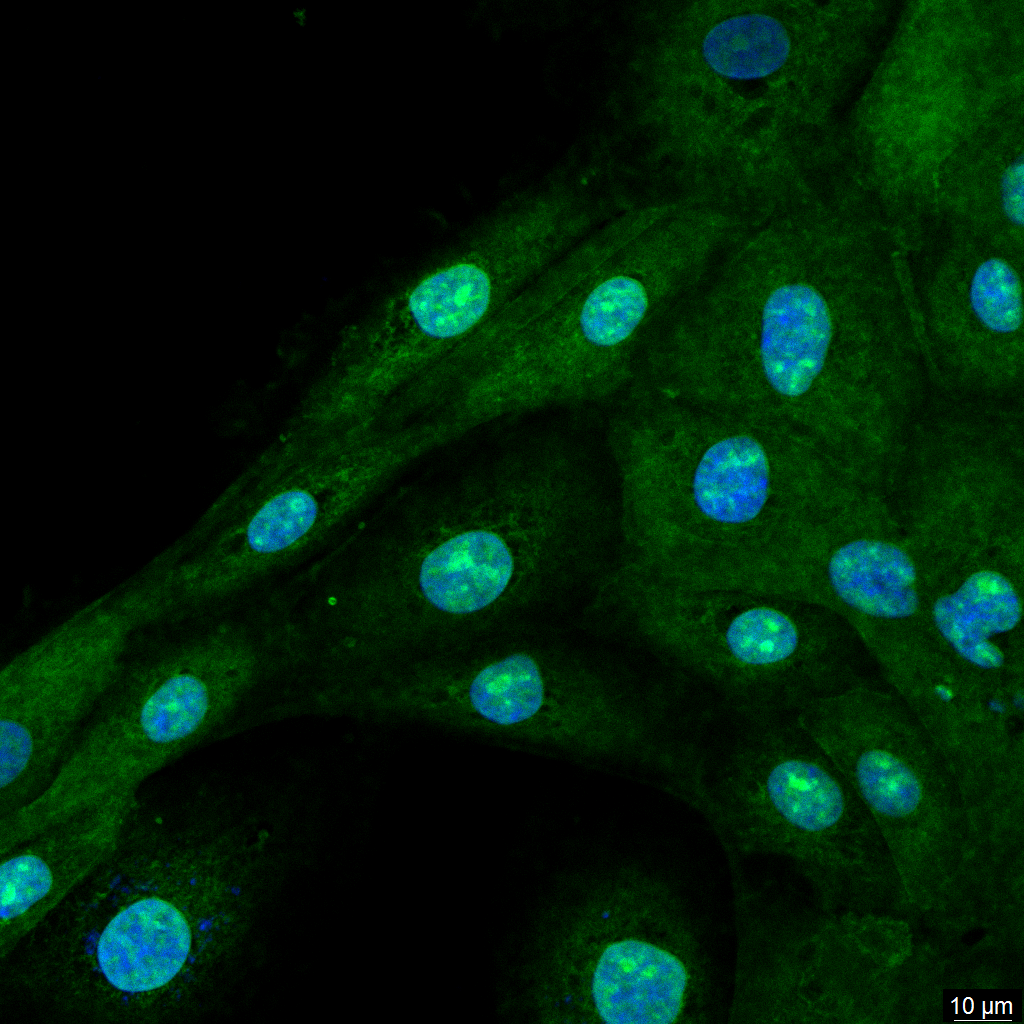

Supplement: Supplementary file 1 [file vetsci-12-00579-s001.zip › File S1/Fig2/IF/LPS+NAC/2024.11.8 Nrf2_11_overlay.tif]

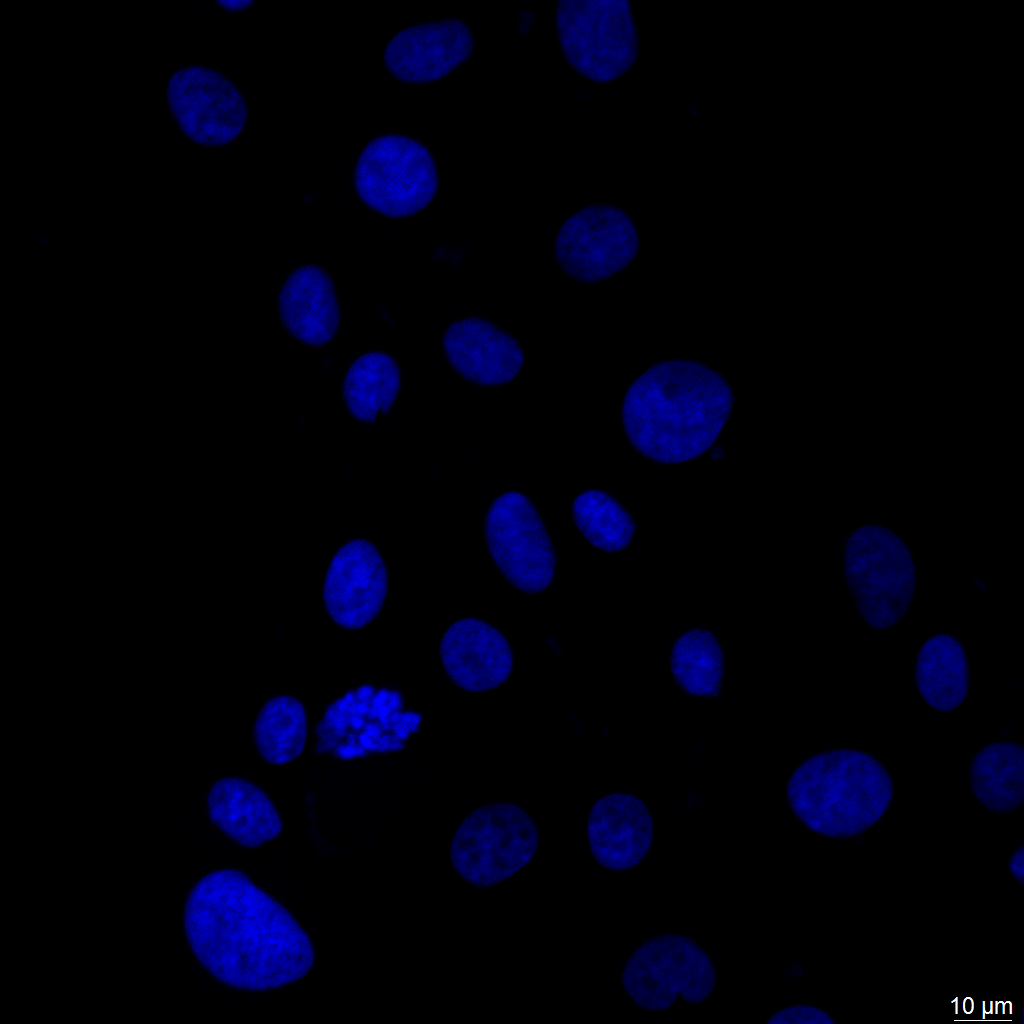

Supplement: Supplementary file 1 [file vetsci-12-00579-s001.zip › File S1/Fig2/IF/L/24.11.8Nrf2_25_ch00_SV.tif]

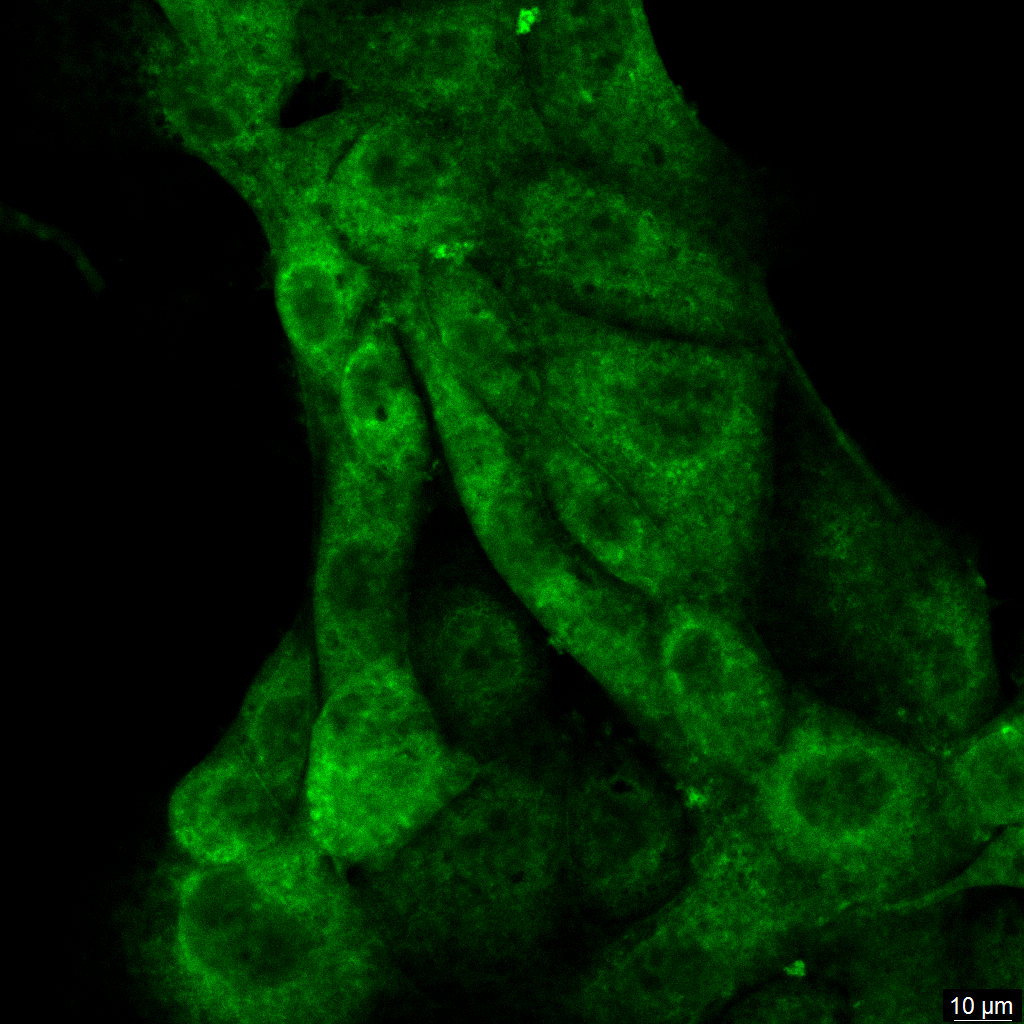

Supplement: Supplementary file 1 [file vetsci-12-00579-s001.zip › File S1/Fig2/IF/L/24.11.8Nrf2_25_ch01_SV.tif]

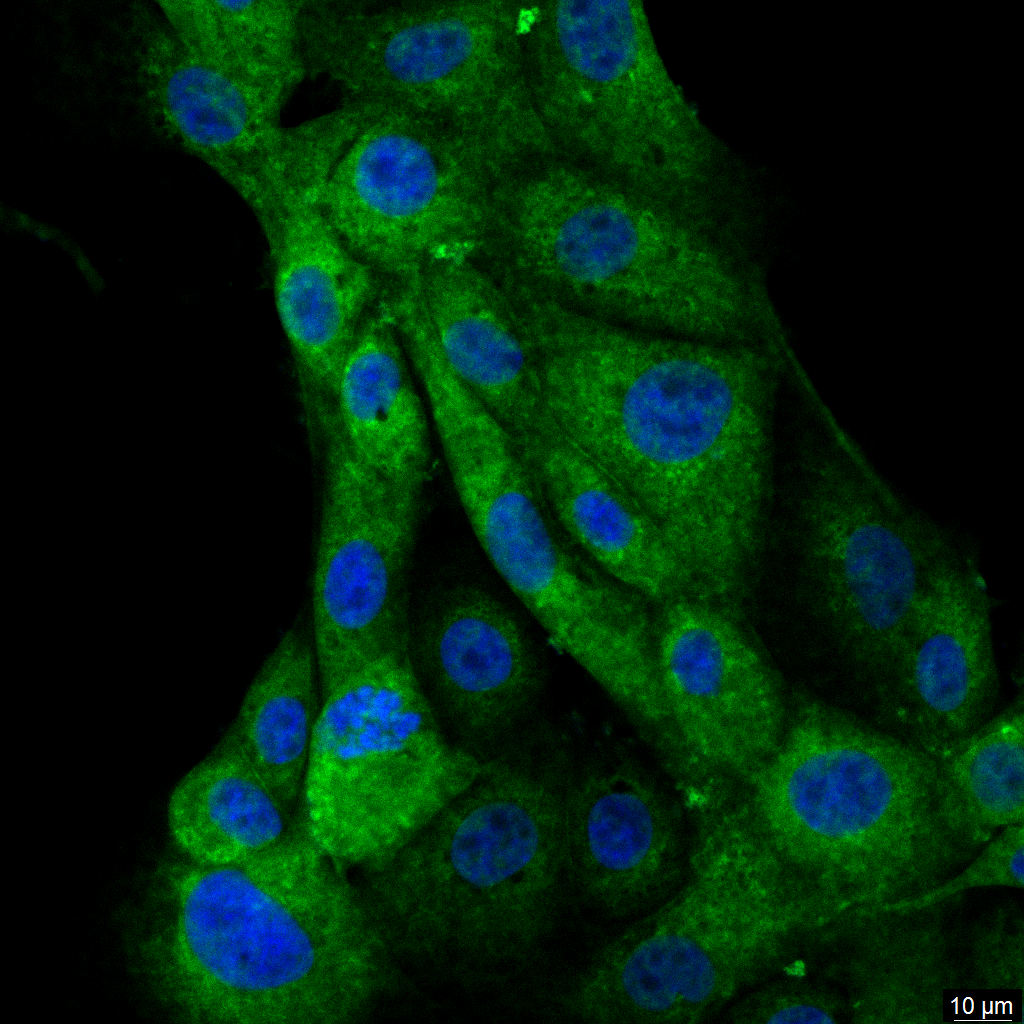

Supplement: Supplementary file 1 [file vetsci-12-00579-s001.zip › File S1/Fig2/IF/L/24.11.8Nrf2_25_overlay.tif]

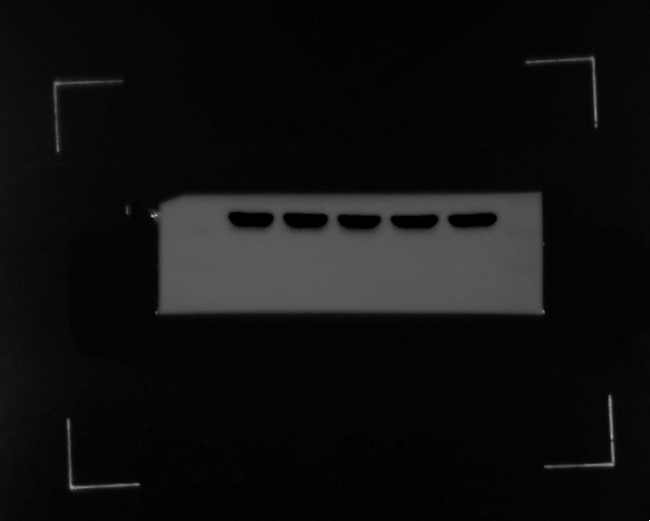

Supplement: Supplementary file 1 [file vetsci-12-00579-s001.zip › File S1/Fig2/WB/BA_1_240807_151530_00.10.000_0_5472.tif]

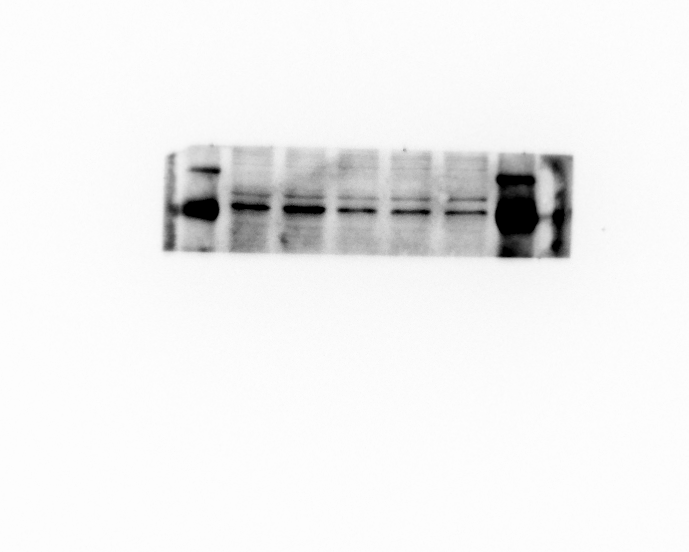

Supplement: Supplementary file 1 [file vetsci-12-00579-s001.zip › File S1/Fig2/WB/COX2_1_240809_155601_00.23.000_1_4312.tif]

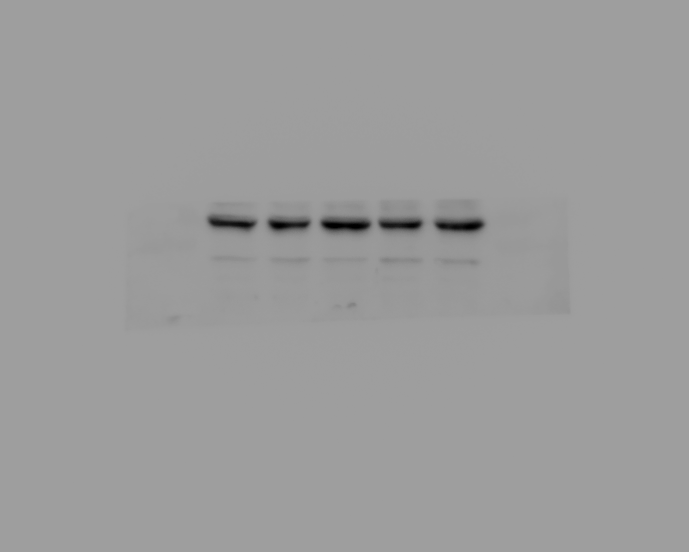

Supplement: Supplementary file 1 [file vetsci-12-00579-s001.zip › File S1/Fig2/WB/HO1_1_240506_174500_00.08.000_1_25261.tif]

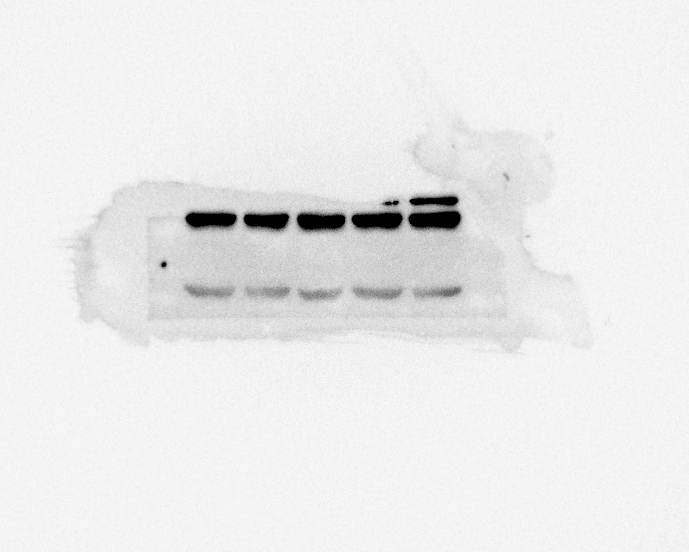

Supplement: Supplementary file 1 [file vetsci-12-00579-s001.zip › File S1/Fig2/WB/LB1_1_240509_094348_00.20.000_1_1000.tif]

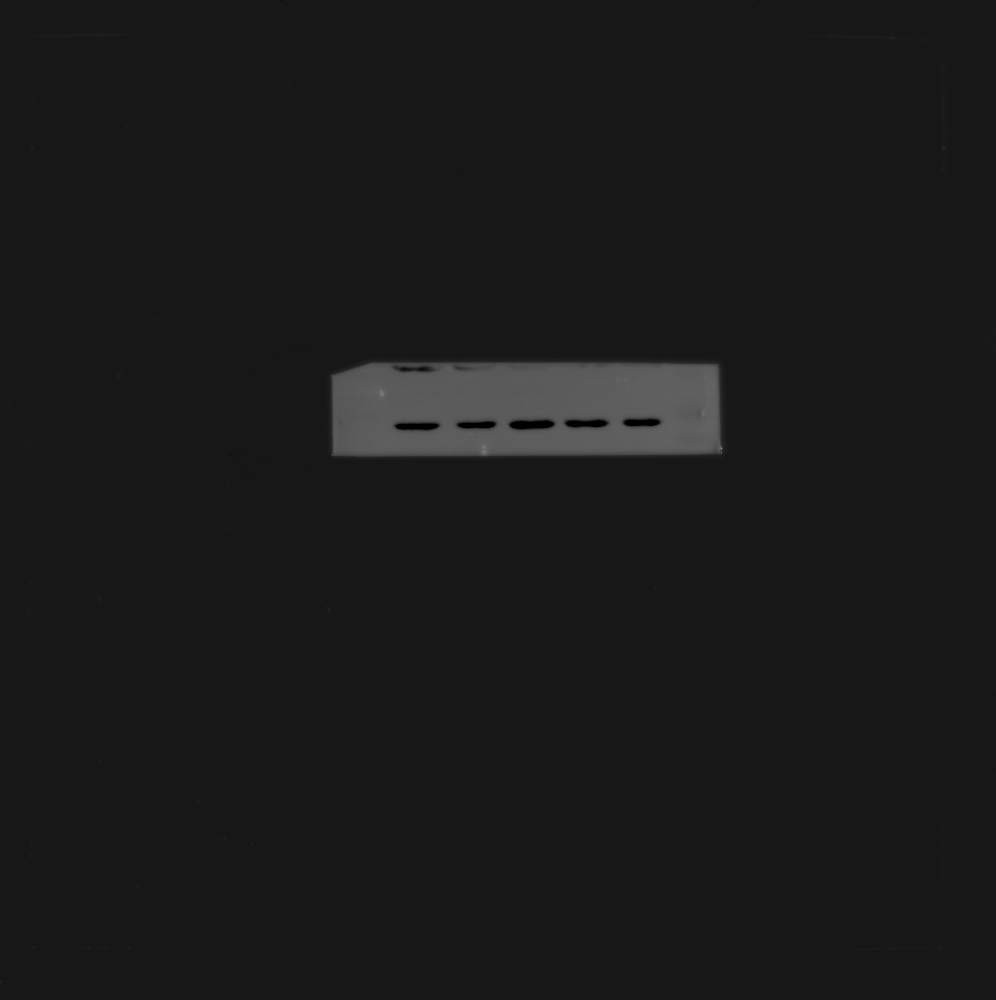

Supplement: Supplementary file 1 [file vetsci-12-00579-s001.zip › File S1/Fig2/WB/NQO1_1_20240528_094523_5_0_0_0_merge_(16bit).tiff]

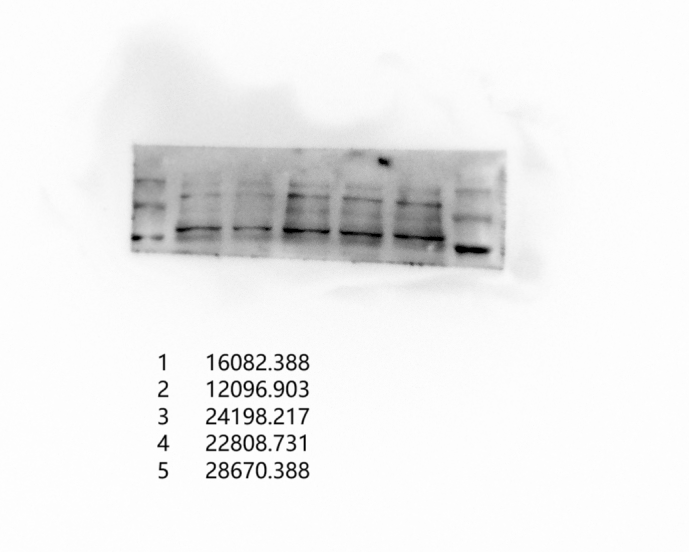

Supplement: Supplementary file 1 [file vetsci-12-00579-s001.zip › File S1/Fig2/WB/NRF2_1_240316_211333_02.00.000_1_4892.tif]

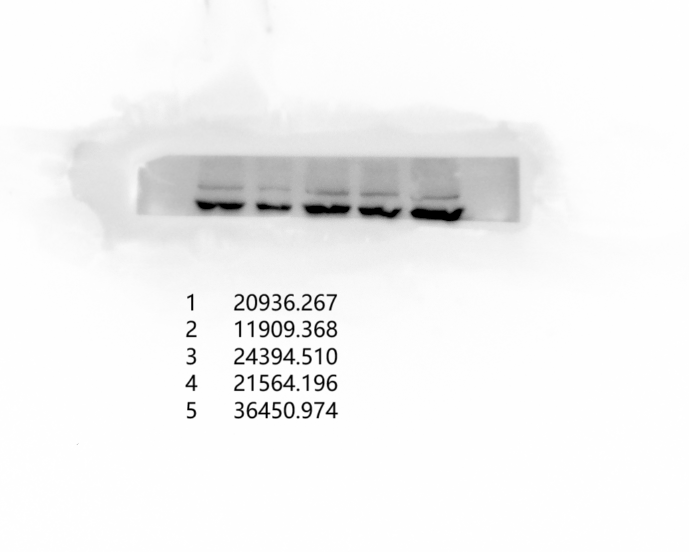

Supplement: Supplementary file 1 [file vetsci-12-00579-s001.zip › File S1/Fig2/WB/║╦NRF2_1_240509_114233_01.30.000_1_12668.tif]

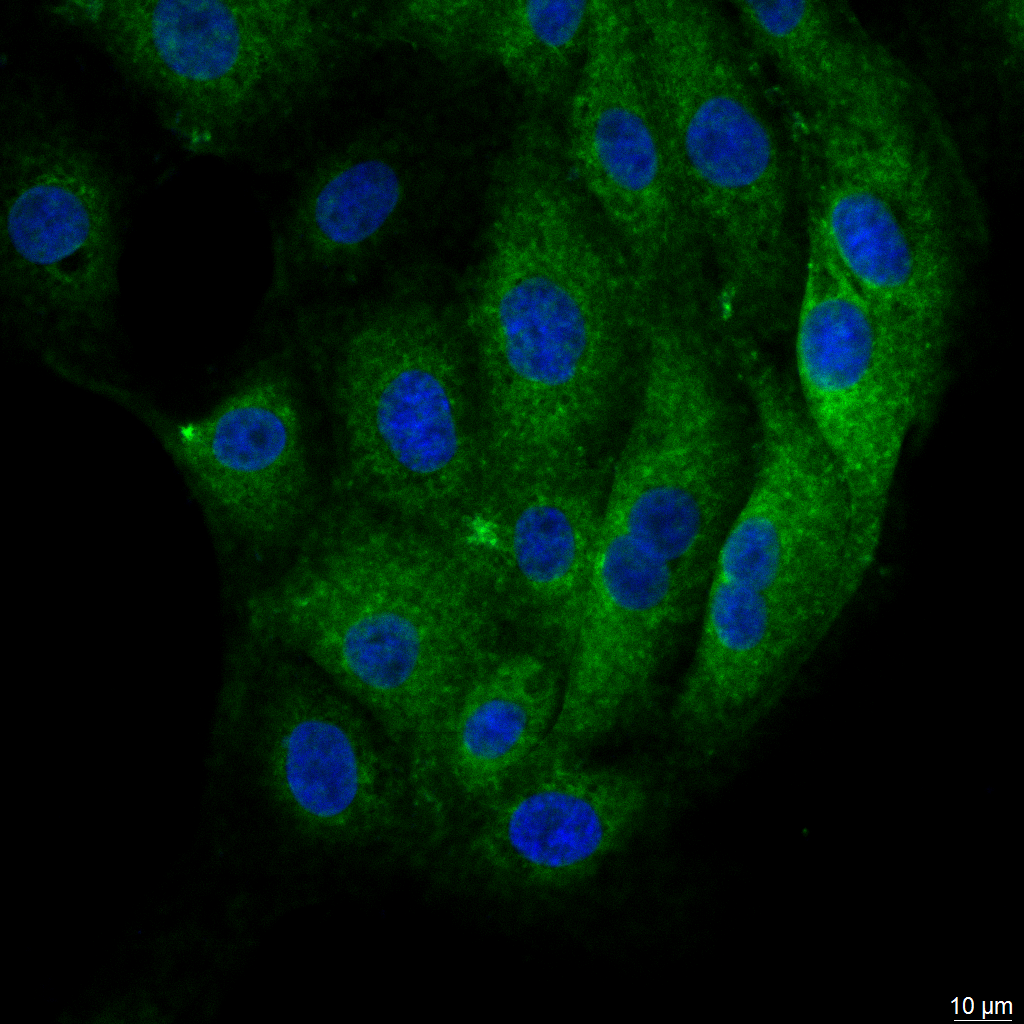

Supplement: Supplementary file 1 [file vetsci-12-00579-s001.zip › File S1/fig4/IF/C/24.11.8Nrf2_27overlay.tif]

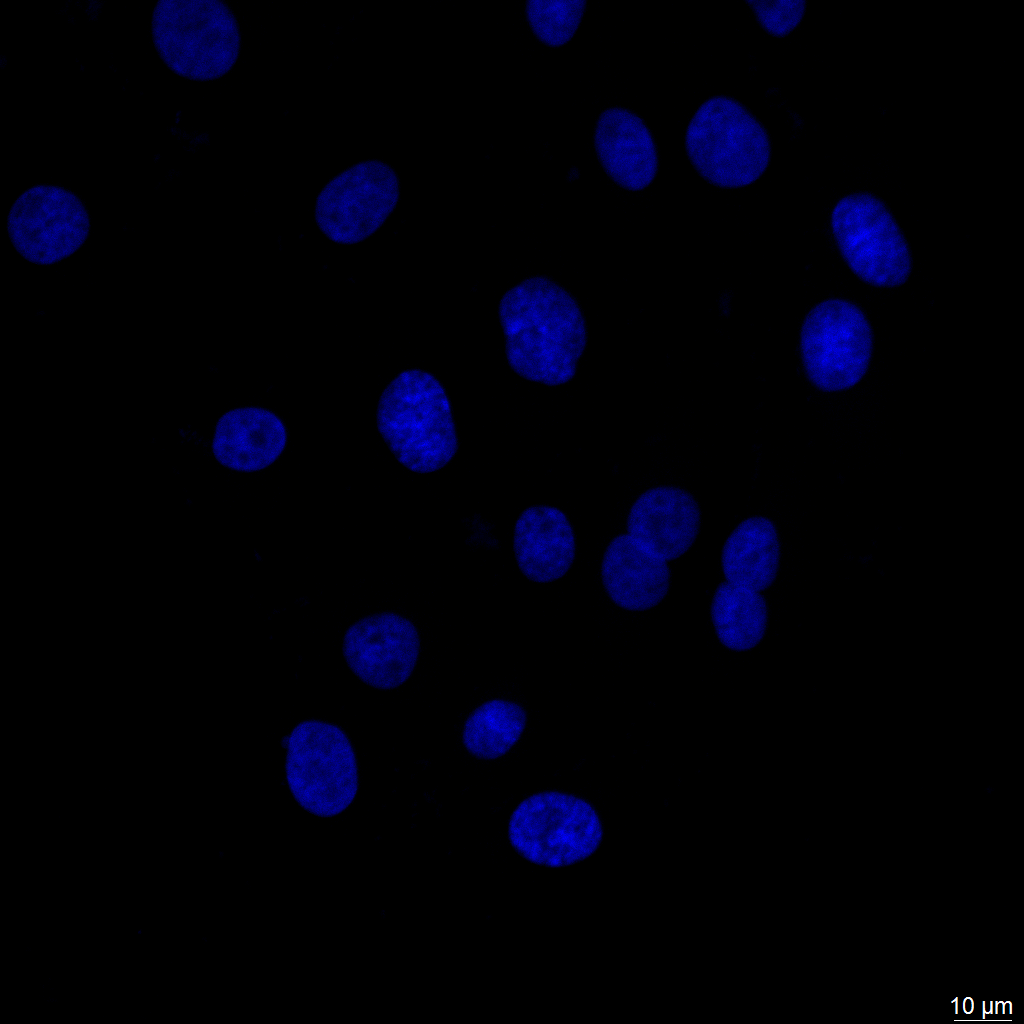

Supplement: Supplementary file 1 [file vetsci-12-00579-s001.zip › File S1/fig4/IF/C/24.11.8Nrf2_27_ch00_SV.tif]

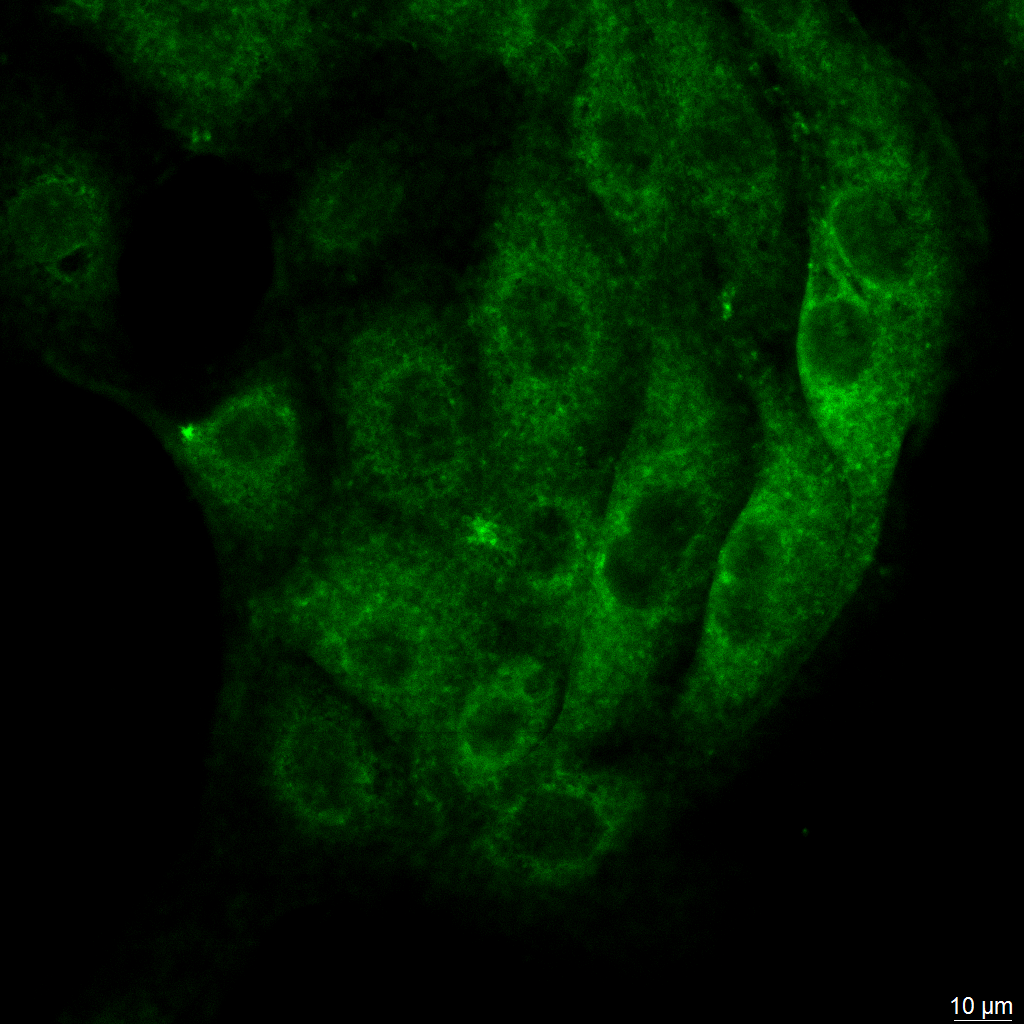

Supplement: Supplementary file 1 [file vetsci-12-00579-s001.zip › File S1/fig4/IF/C/24.11.8Nrf2_27_ch01_SV.tif]

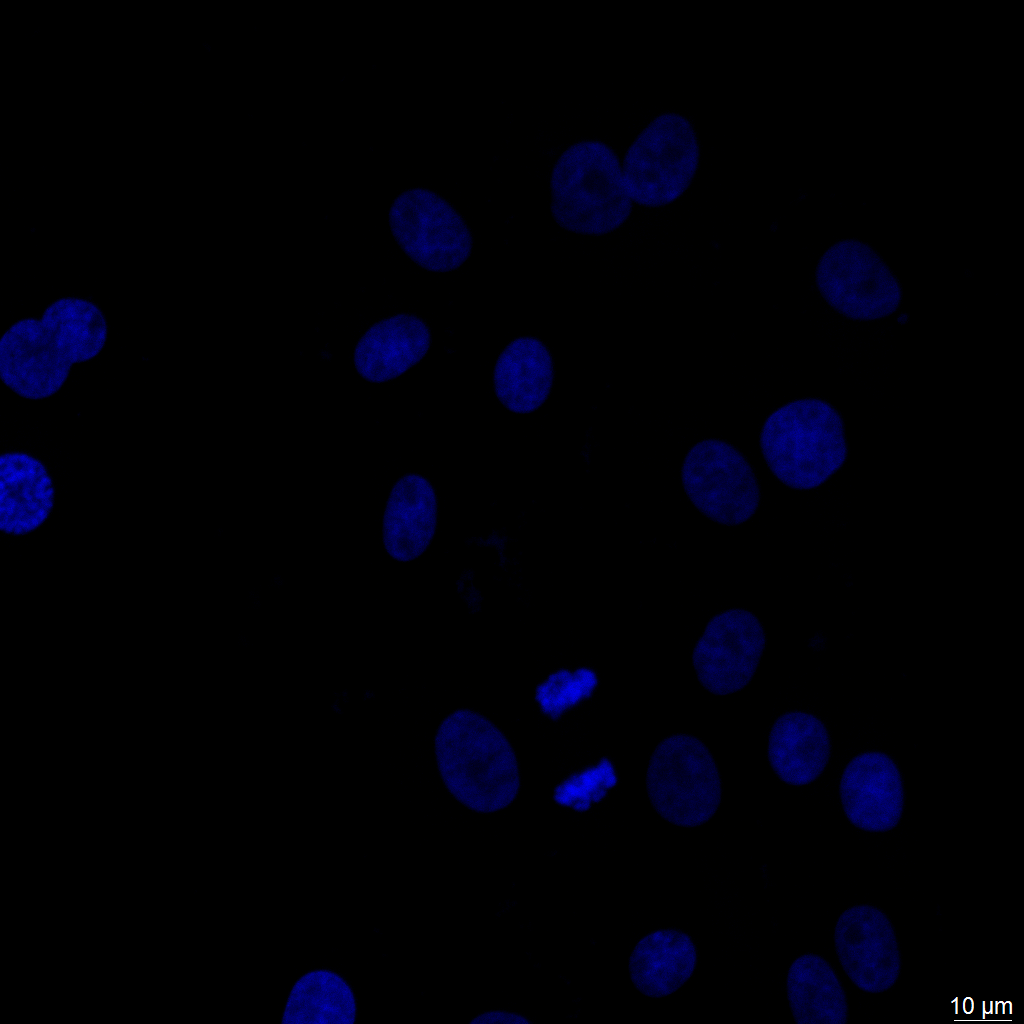

Supplement: Supplementary file 1 [file vetsci-12-00579-s001.zip › File S1/fig4/IF/LPS+MEL+ZnPP/24.11.8Nrf2_26_ch00_SV.tif]

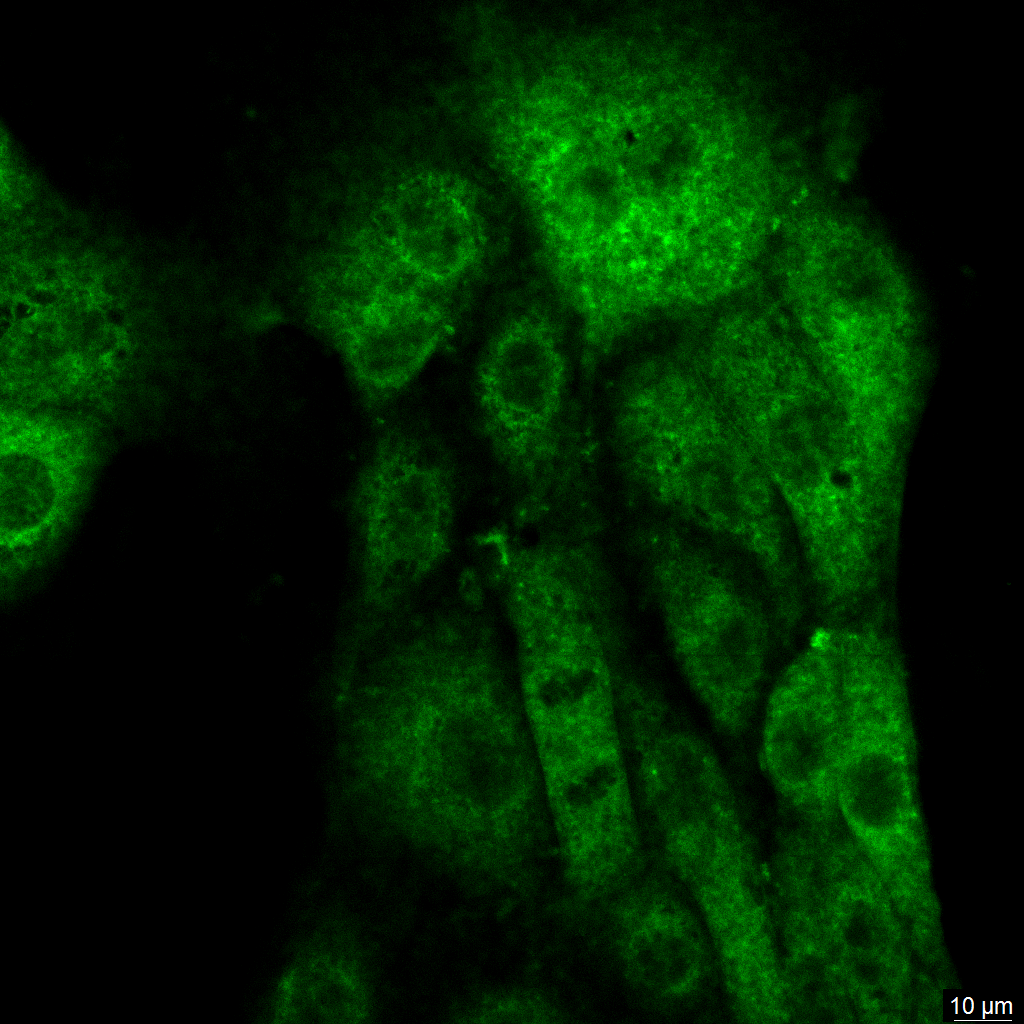

Supplement: Supplementary file 1 [file vetsci-12-00579-s001.zip › File S1/fig4/IF/LPS+MEL+ZnPP/24.11.8Nrf2_26_ch01_SV.tif]

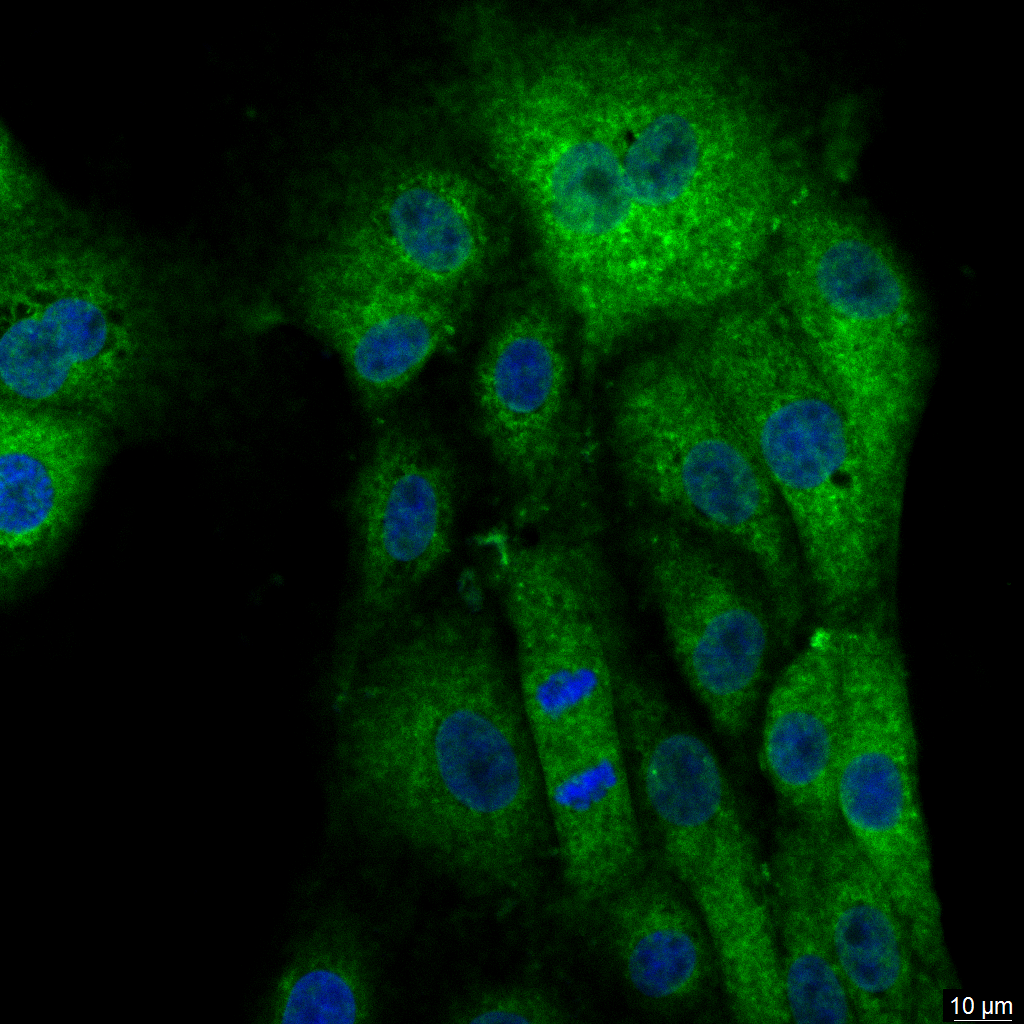

Supplement: Supplementary file 1 [file vetsci-12-00579-s001.zip › File S1/fig4/IF/LPS+MEL+ZnPP/24.11.8Nrf2_26_overlay.tif]

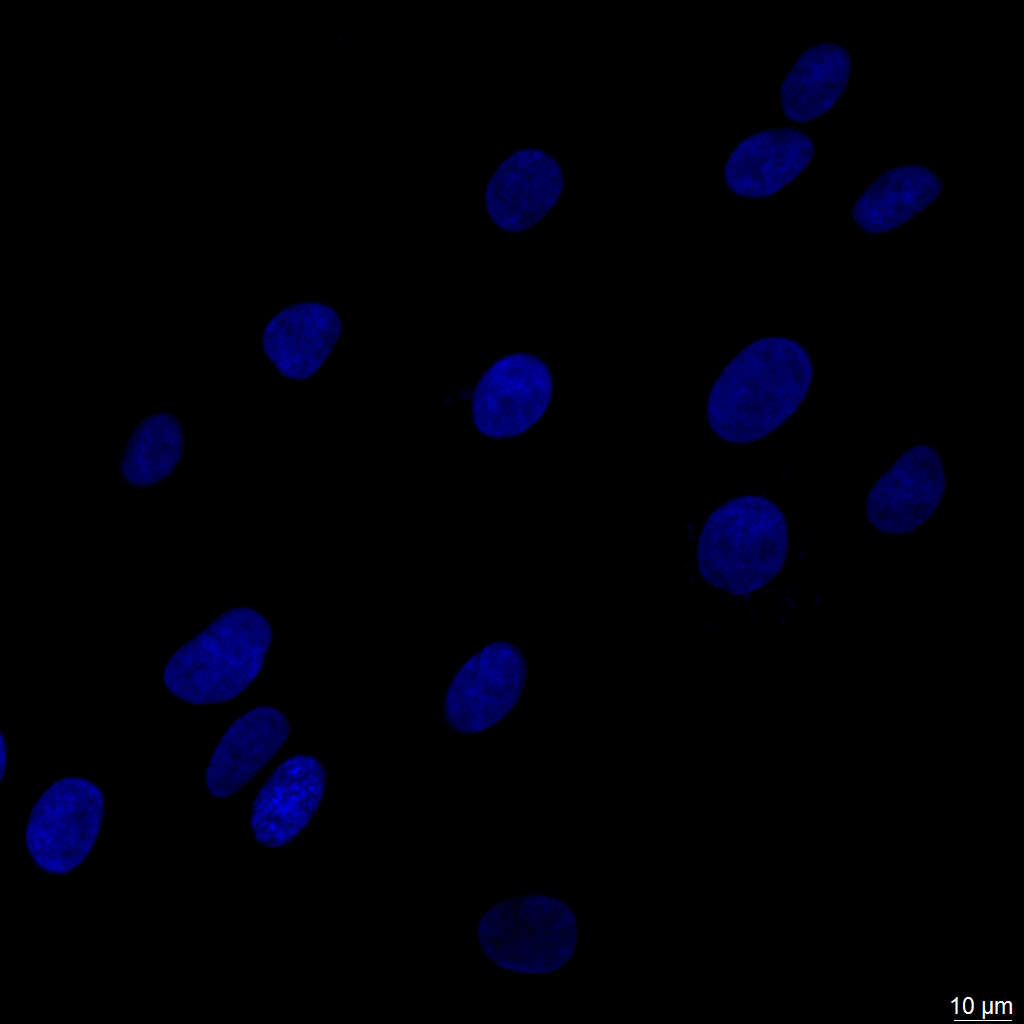

Supplement: Supplementary file 1 [file vetsci-12-00579-s001.zip › File S1/fig4/IF/LPS+MEL/2024.11.8 Nrf2_12_ch00_SV.tif]

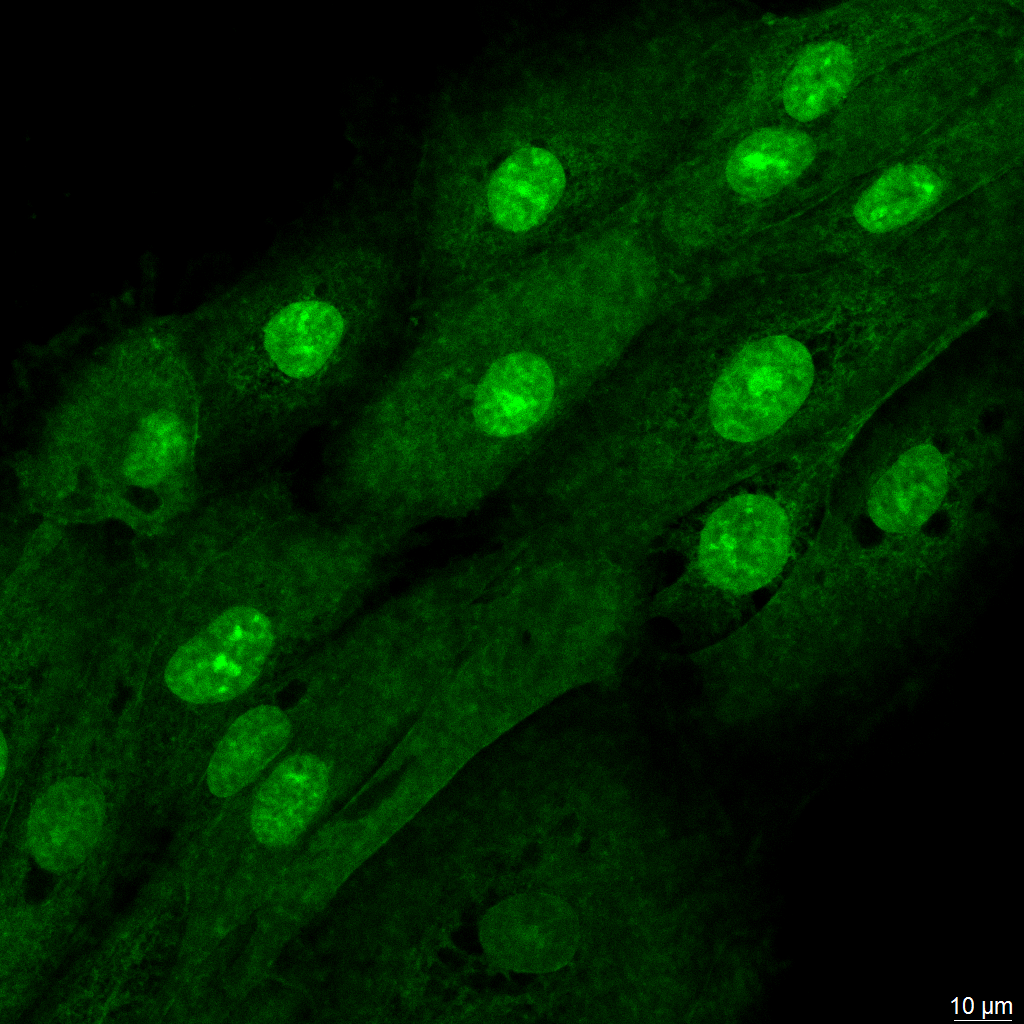

Supplement: Supplementary file 1 [file vetsci-12-00579-s001.zip › File S1/fig4/IF/LPS+MEL/2024.11.8 Nrf2_12_ch01_SV.tif]

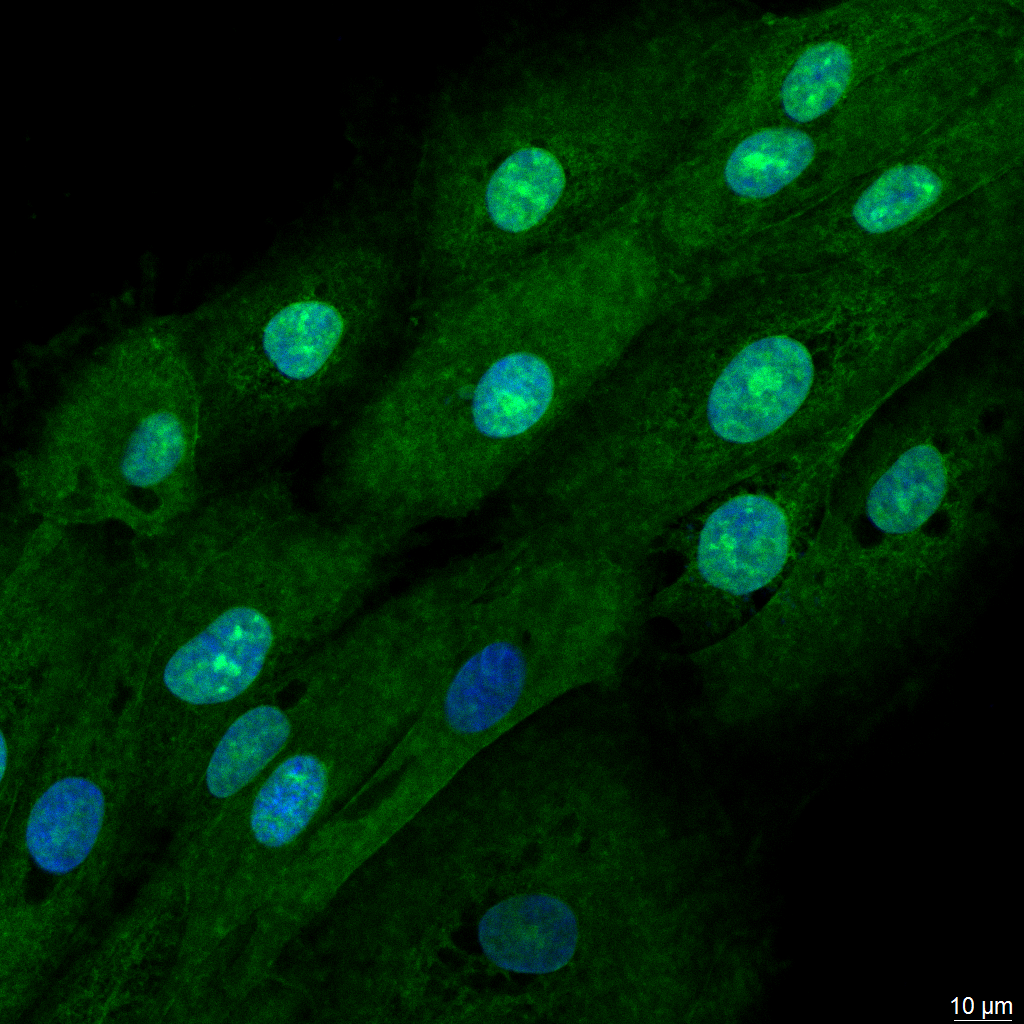

Supplement: Supplementary file 1 [file vetsci-12-00579-s001.zip › File S1/fig4/IF/LPS+MEL/2024.11.8 Nrf2_12_overlay.tif]

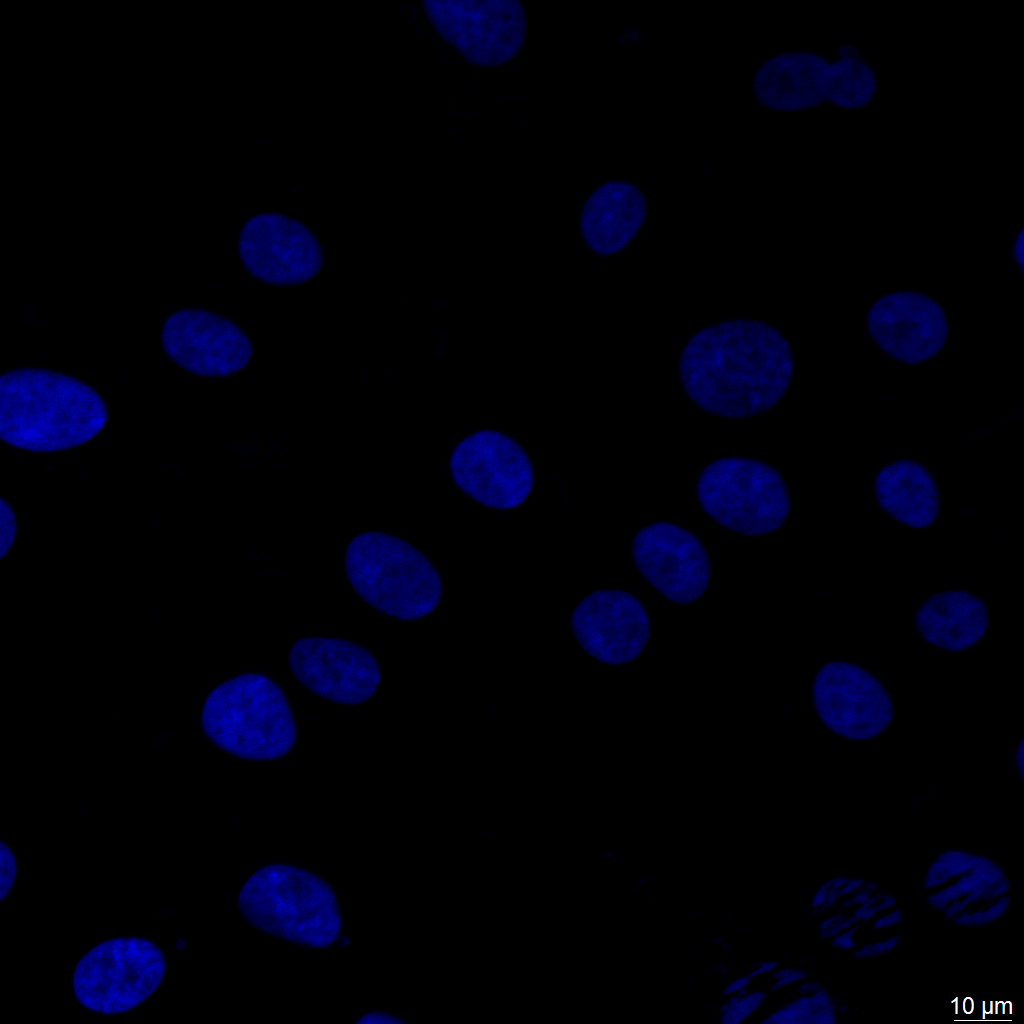

Supplement: Supplementary file 1 [file vetsci-12-00579-s001.zip › File S1/fig4/IF/LPS+ZnPP/24.11.8Nrf2_6_ch00_SV.tif]

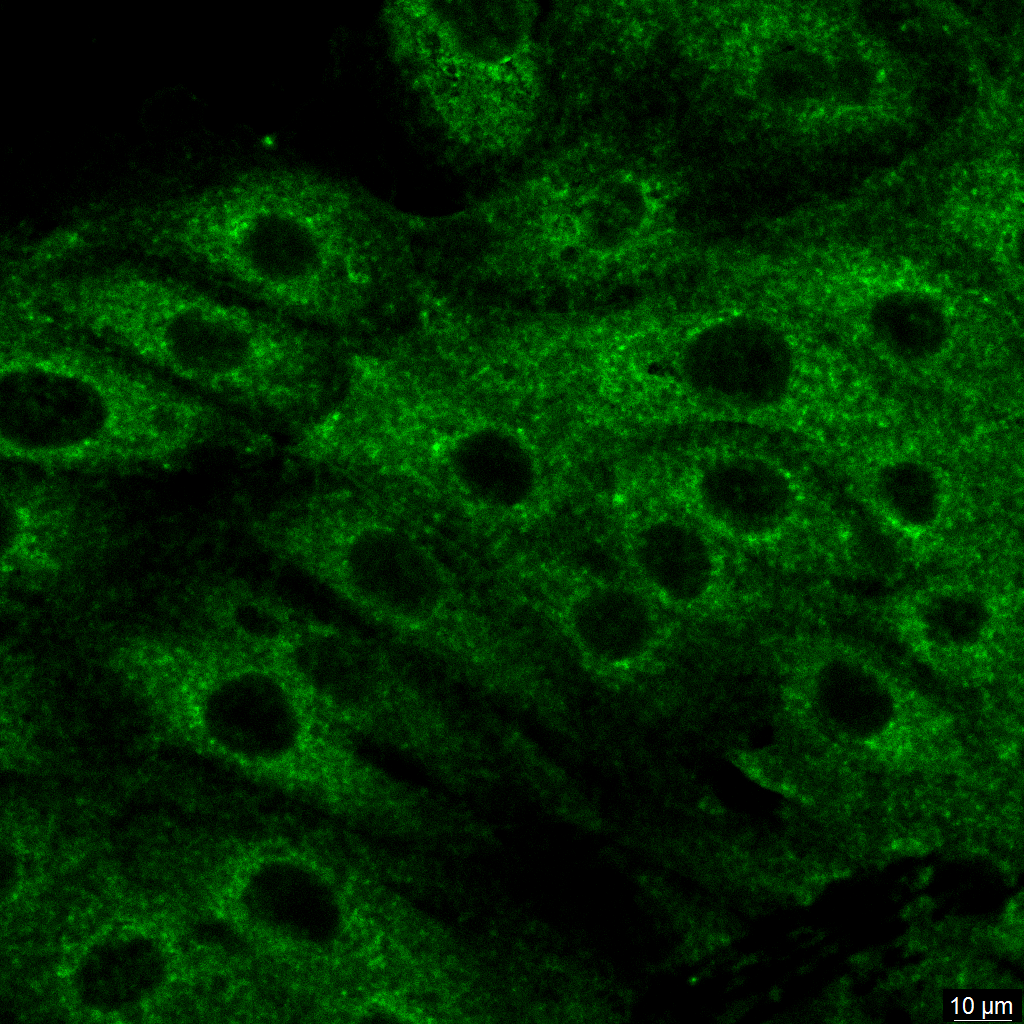

Supplement: Supplementary file 1 [file vetsci-12-00579-s001.zip › File S1/fig4/IF/LPS+ZnPP/24.11.8Nrf2_6_ch01_SV.tif]

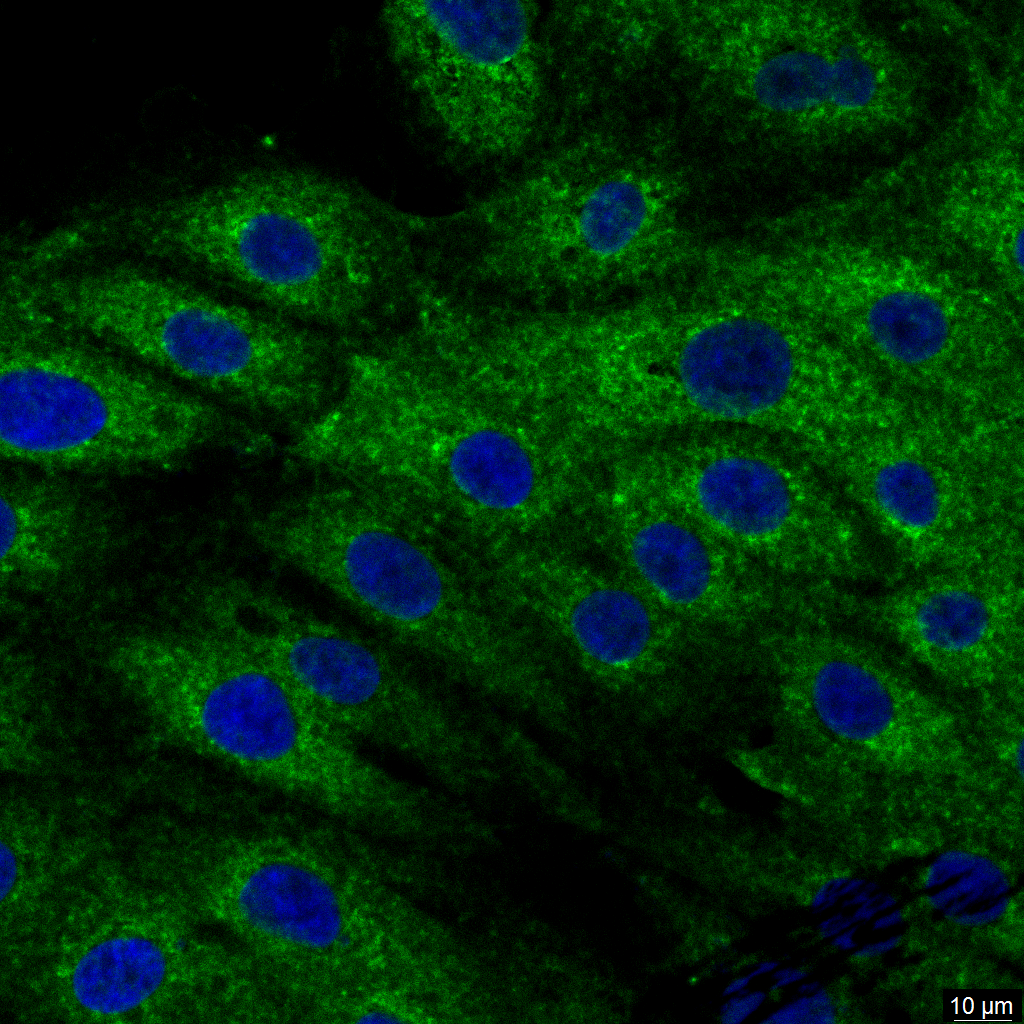

Supplement: Supplementary file 1 [file vetsci-12-00579-s001.zip › File S1/fig4/IF/LPS+ZnPP/24.11.8Nrf2_6_overlay.tif]

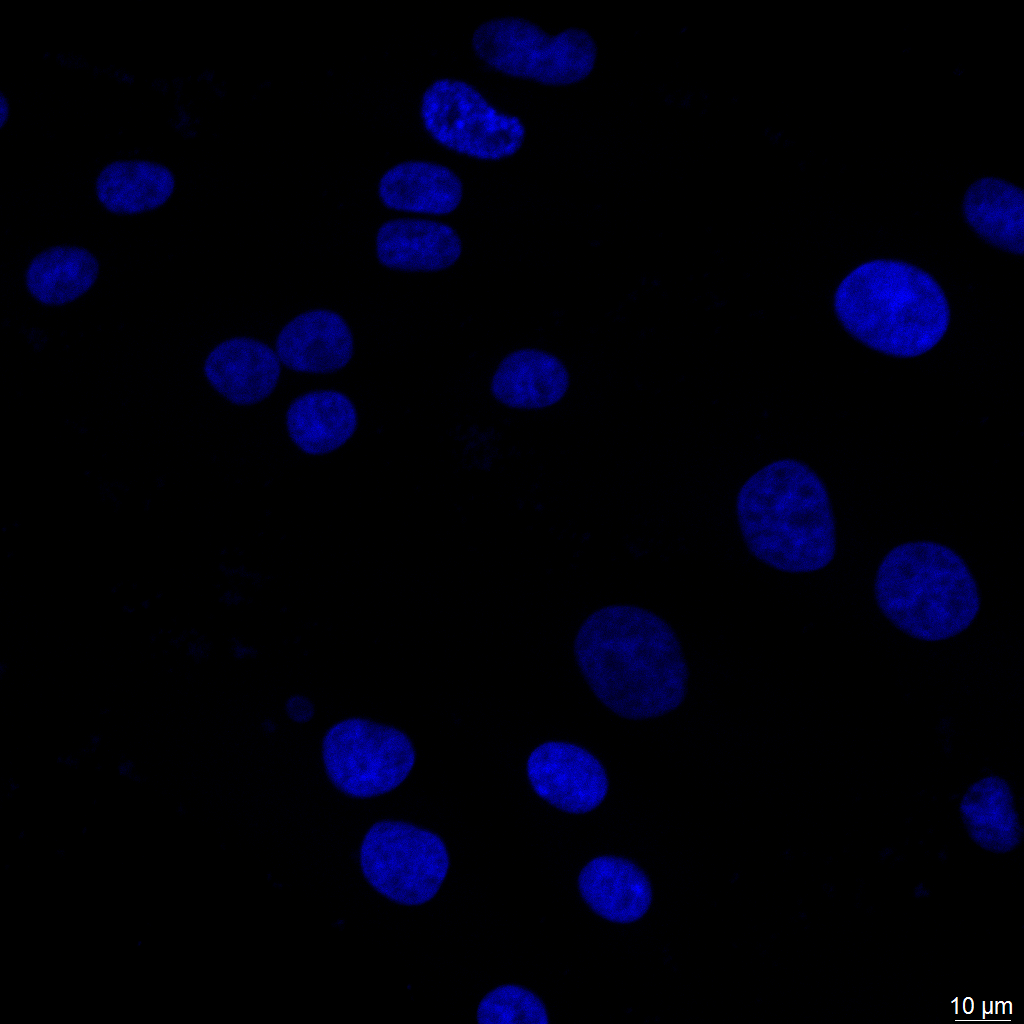

Supplement: Supplementary file 1 [file vetsci-12-00579-s001.zip › File S1/fig4/IF/L/Nrf2 11_19_ch00_SV.tif]

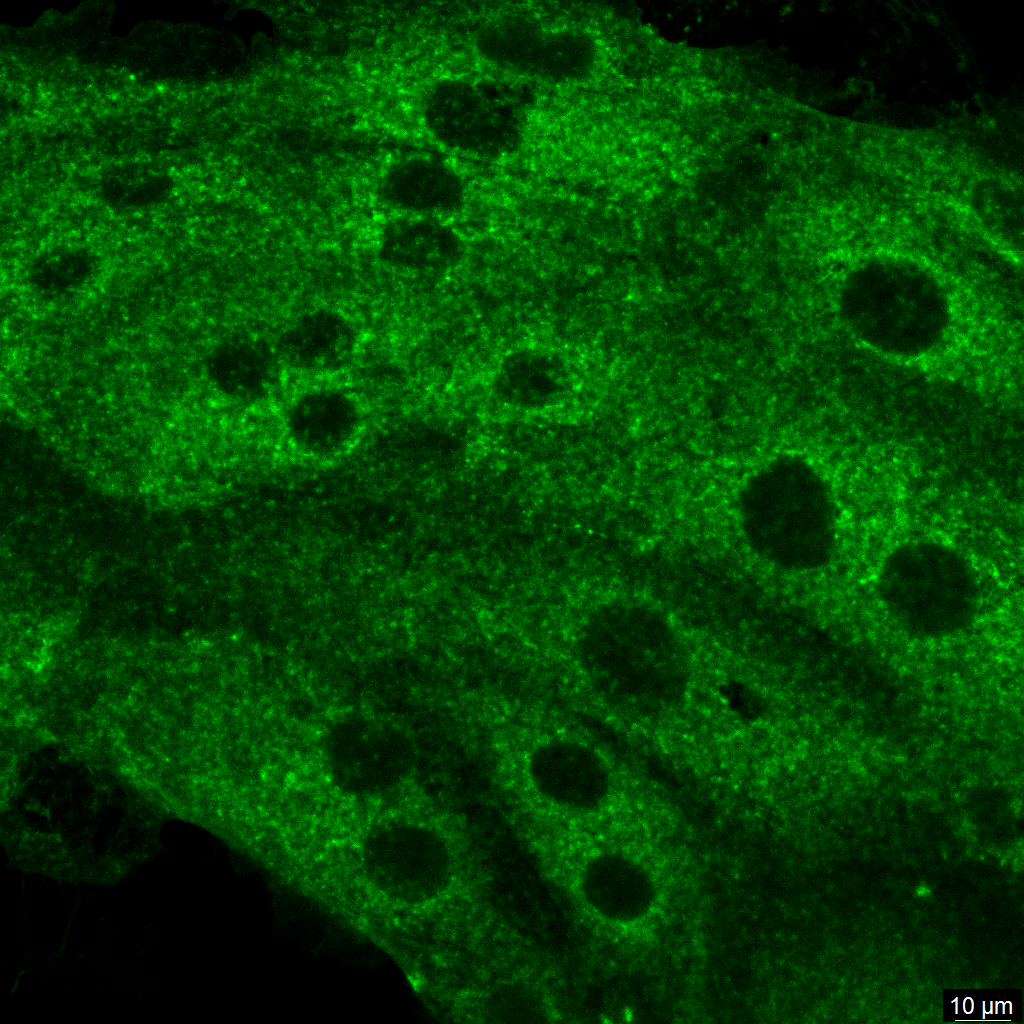

Supplement: Supplementary file 1 [file vetsci-12-00579-s001.zip › File S1/fig4/IF/L/Nrf2 11_19_ch01_SV.tif]

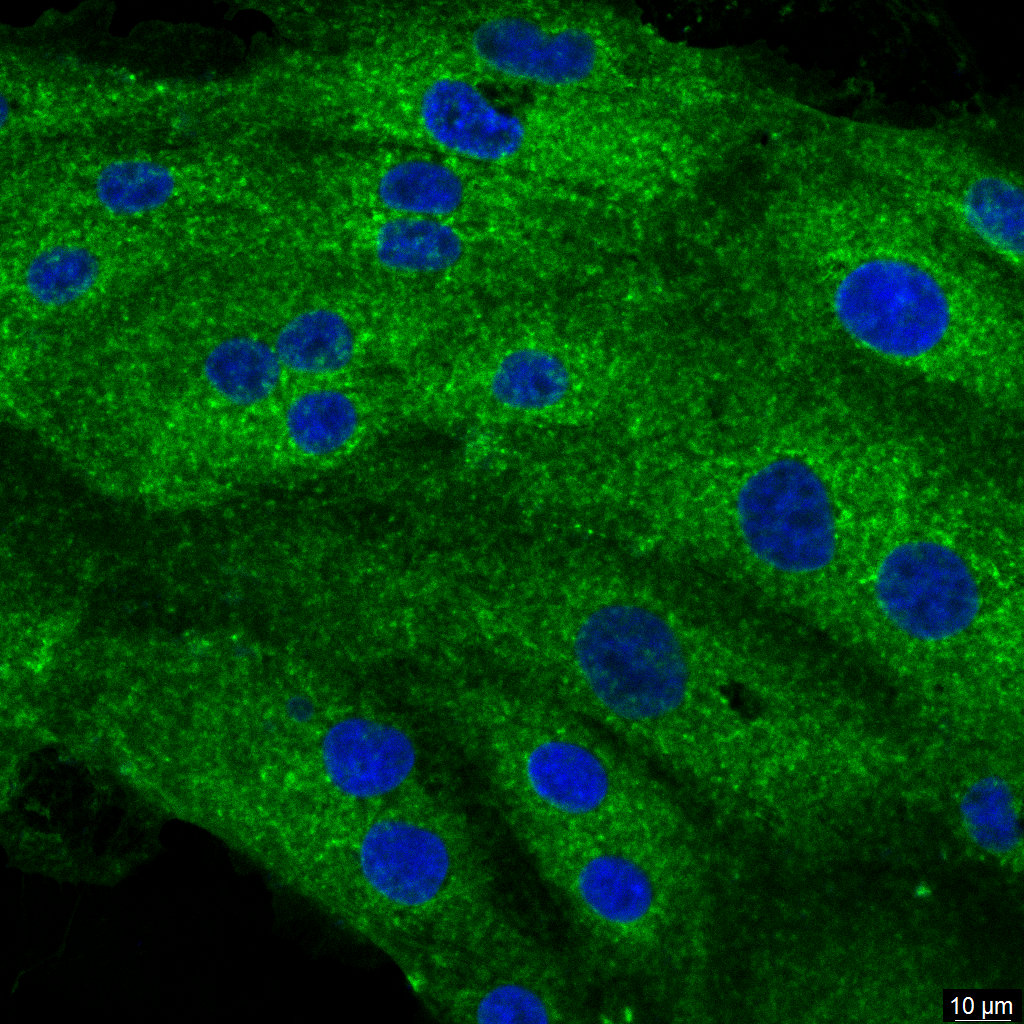

Supplement: Supplementary file 1 [file vetsci-12-00579-s001.zip › File S1/fig4/IF/L/Nrf211_19_overlay.tif]

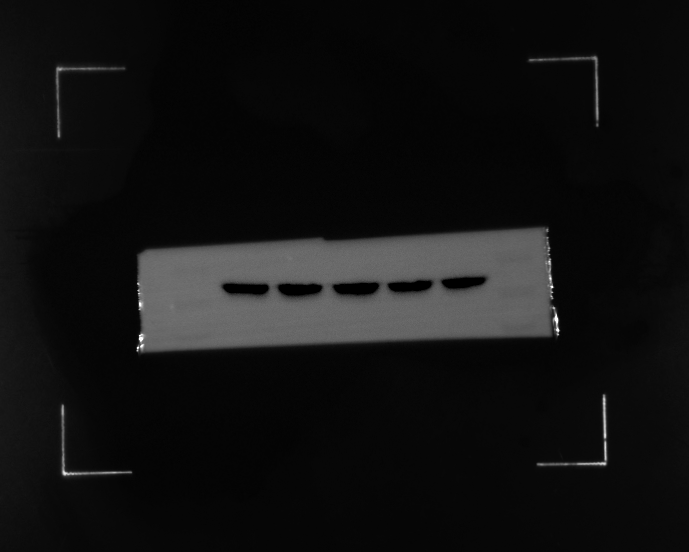

Supplement: Supplementary file 1 [file vetsci-12-00579-s001.zip › File S1/fig4/WB/BA_1_241006_211742_00.13.000_0_7909.tif]

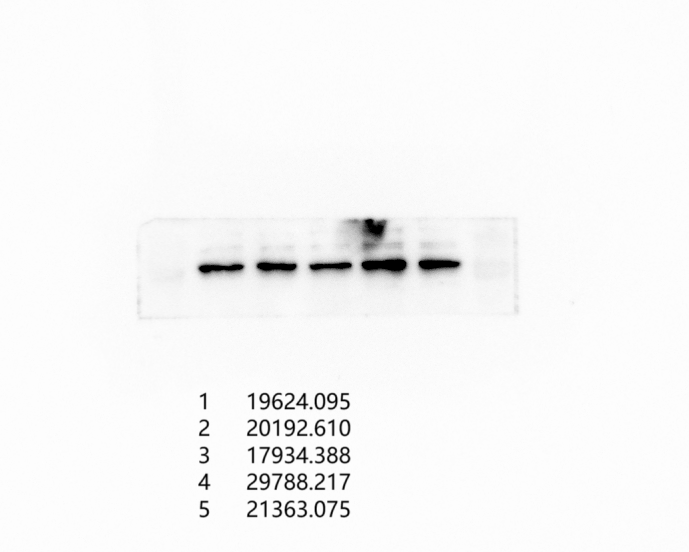

Supplement: Supplementary file 1 [file vetsci-12-00579-s001.zip › File S1/fig4/WB/COX2_1_241025_205213_00.07.000_1_4000.tif]

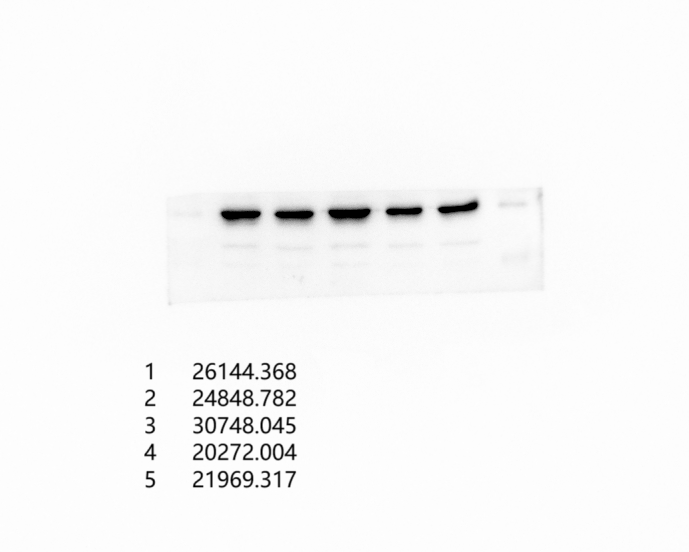

Supplement: Supplementary file 1 [file vetsci-12-00579-s001.zip › File S1/fig4/WB/ho1_1_241006_123745_00.25.000_1_5000.tif]

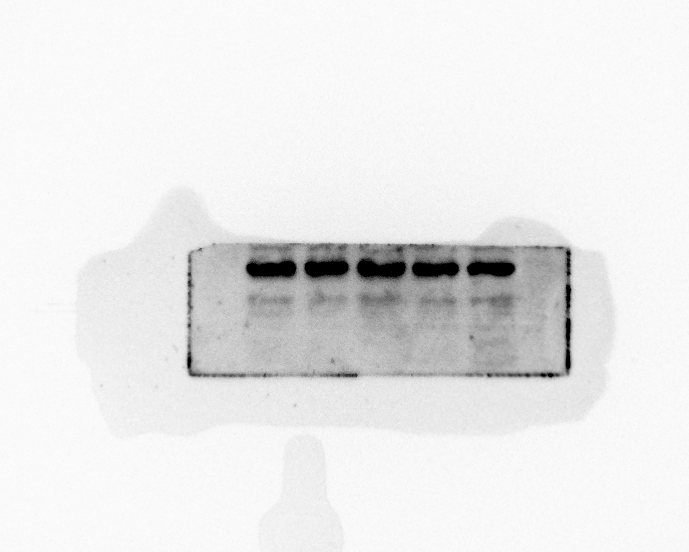

Supplement: Supplementary file 1 [file vetsci-12-00579-s001.zip › File S1/fig4/WB/LB1_1_241121_102221_00.22.000_1_1800.tif]

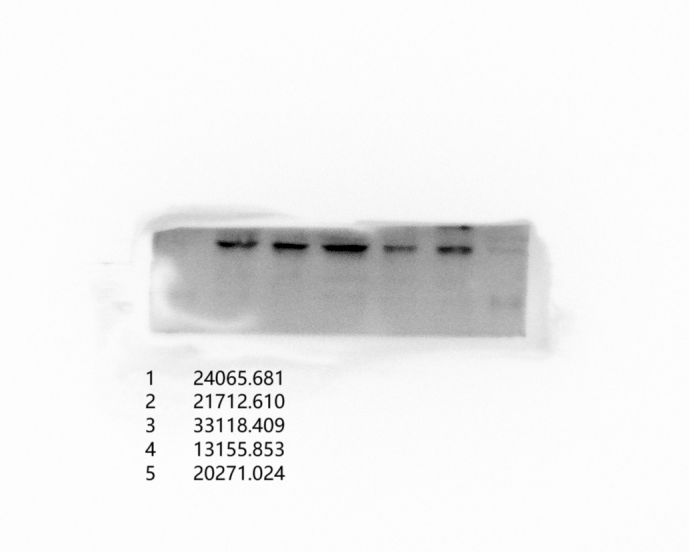

Supplement: Supplementary file 1 [file vetsci-12-00579-s001.zip › File S1/fig4/WB/nqo1_1_241009_195854_01.00.000_2_3982.tif]

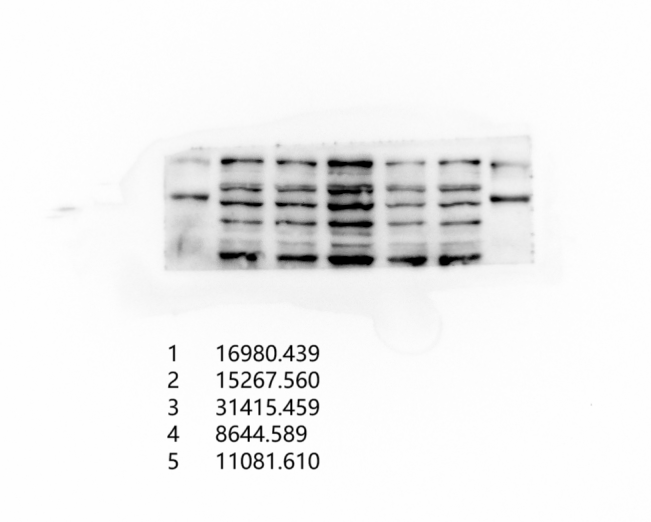

Supplement: Supplementary file 1 [file vetsci-12-00579-s001.zip › File S1/fig4/WB/NRF2_1_241006_120600_04.35.000_1_6760.tif]

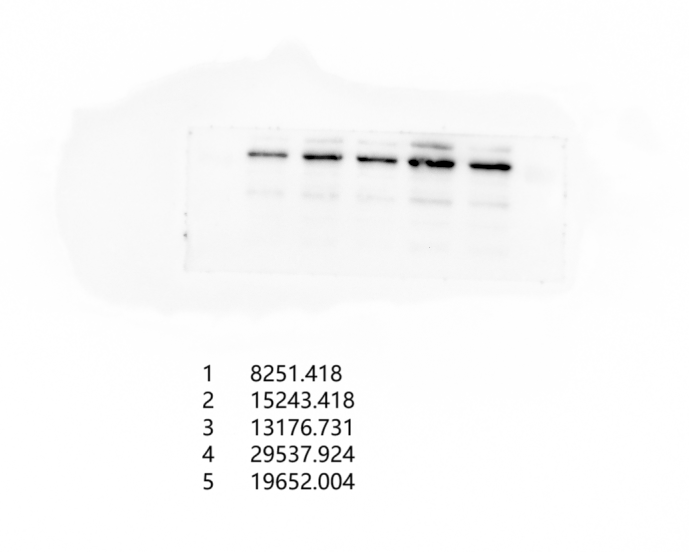

Supplement: Supplementary file 1 [file vetsci-12-00579-s001.zip › File S1/fig4/WB/P65_1_241123_105803_02.30.000_1_14868.tif]

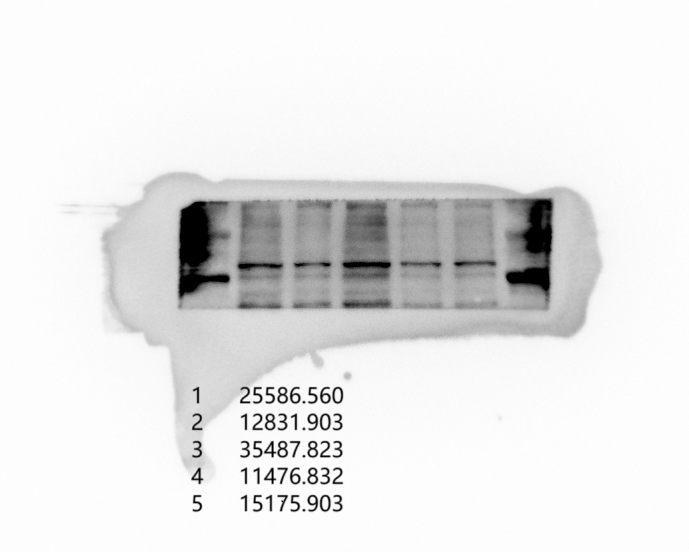

Supplement: Supplementary file 1 [file vetsci-12-00579-s001.zip › File S1/fig4/WB/║╦NRF2_1_241121_100609_02.42.000_1_5200.tif]

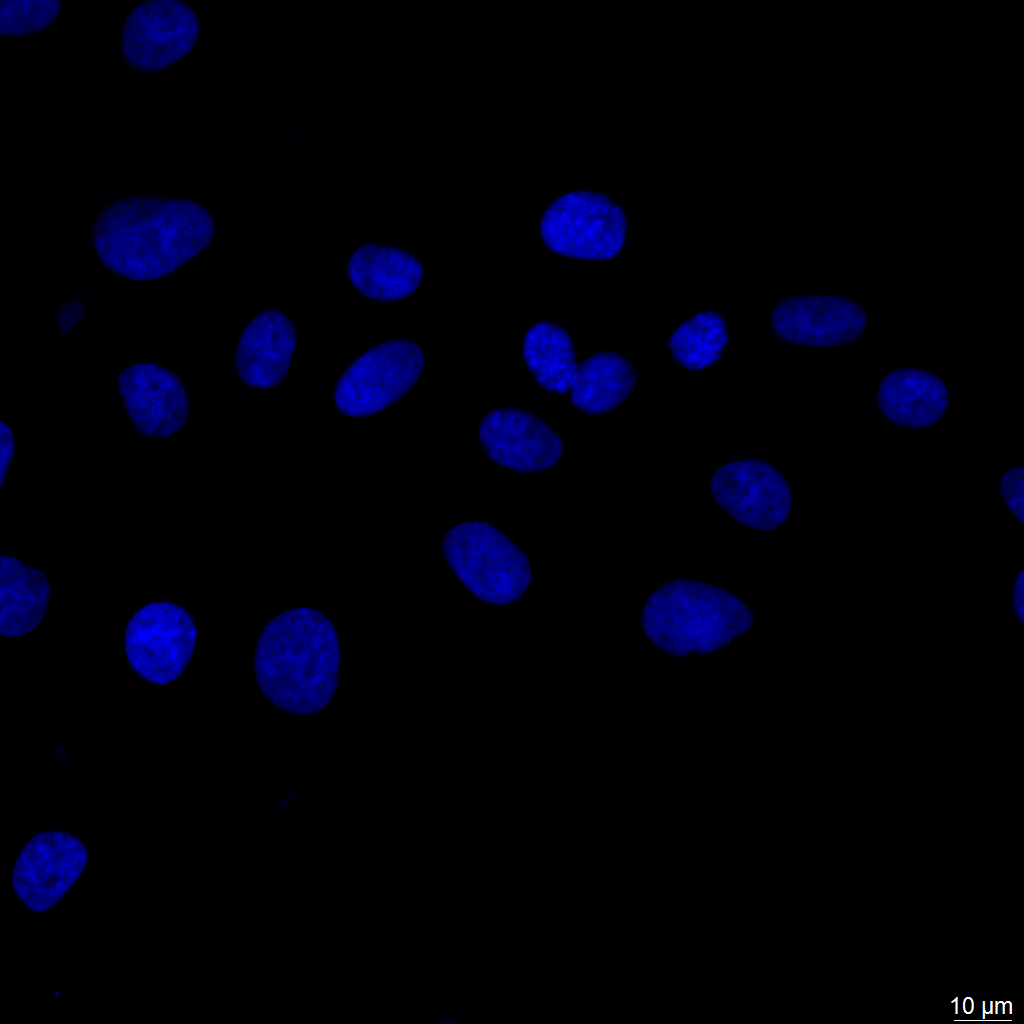

Supplement: Supplementary file 1 [file vetsci-12-00579-s001.zip › File S1/fig6/IF/C/24.11.8Nrf2_21_ch00_SV.tif]

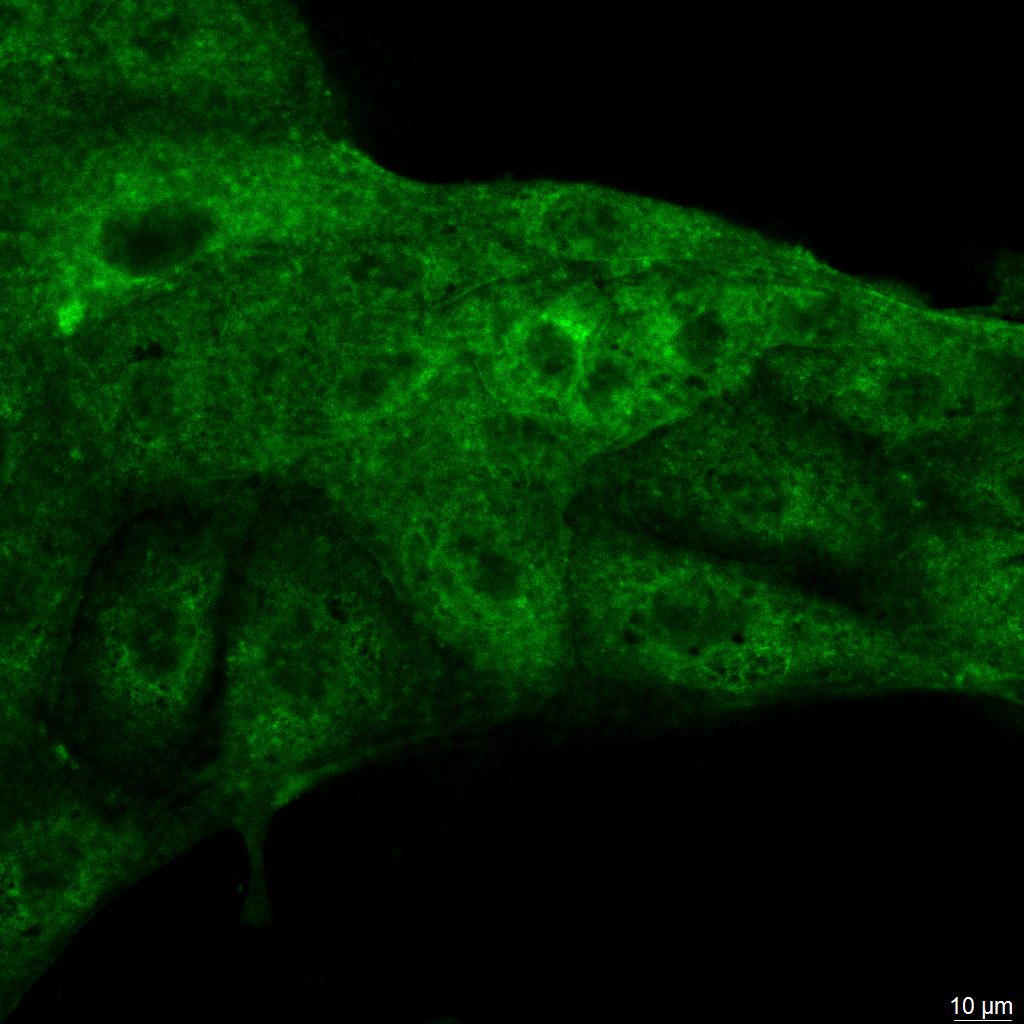

Supplement: Supplementary file 1 [file vetsci-12-00579-s001.zip › File S1/fig6/IF/C/24.11.8Nrf2_21_ch01_SV.tif]

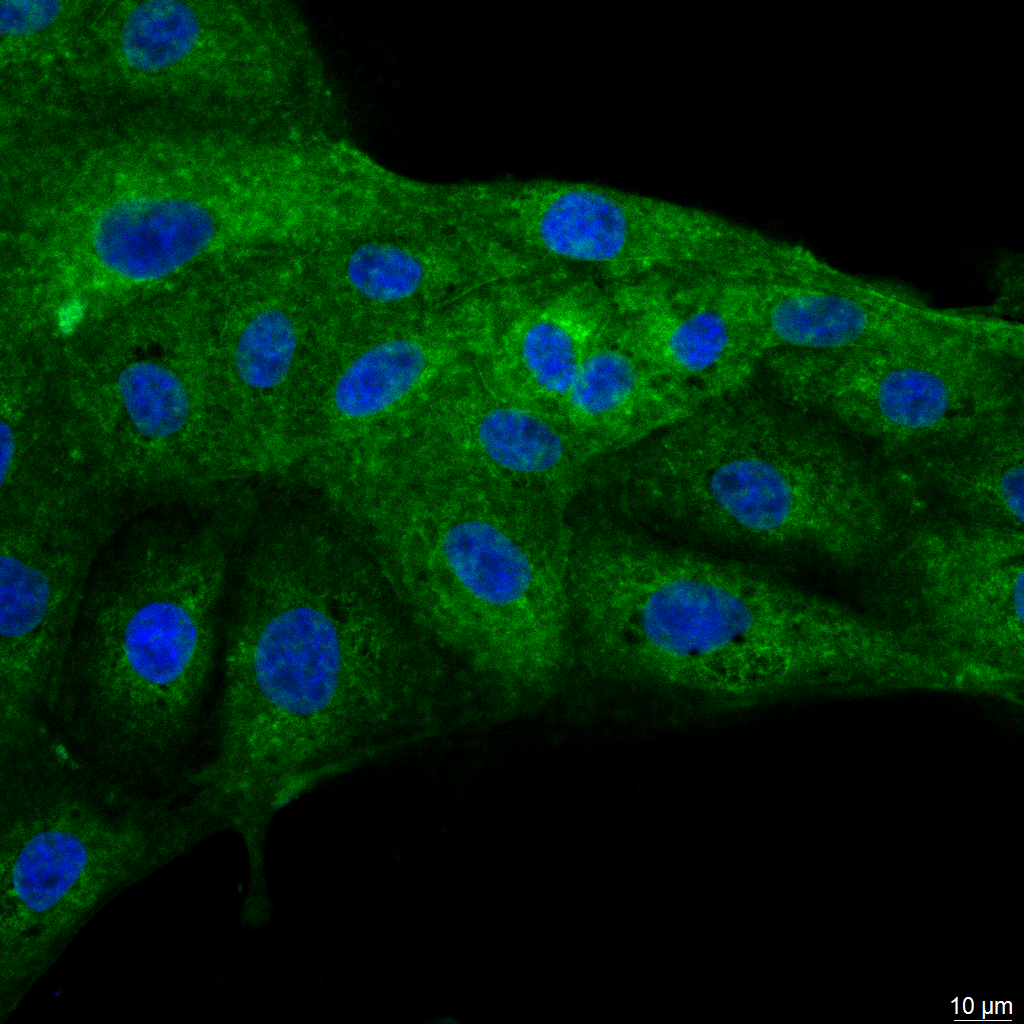

Supplement: Supplementary file 1 [file vetsci-12-00579-s001.zip › File S1/fig6/IF/C/24.11.8Nrf2_21_overlay.tif]

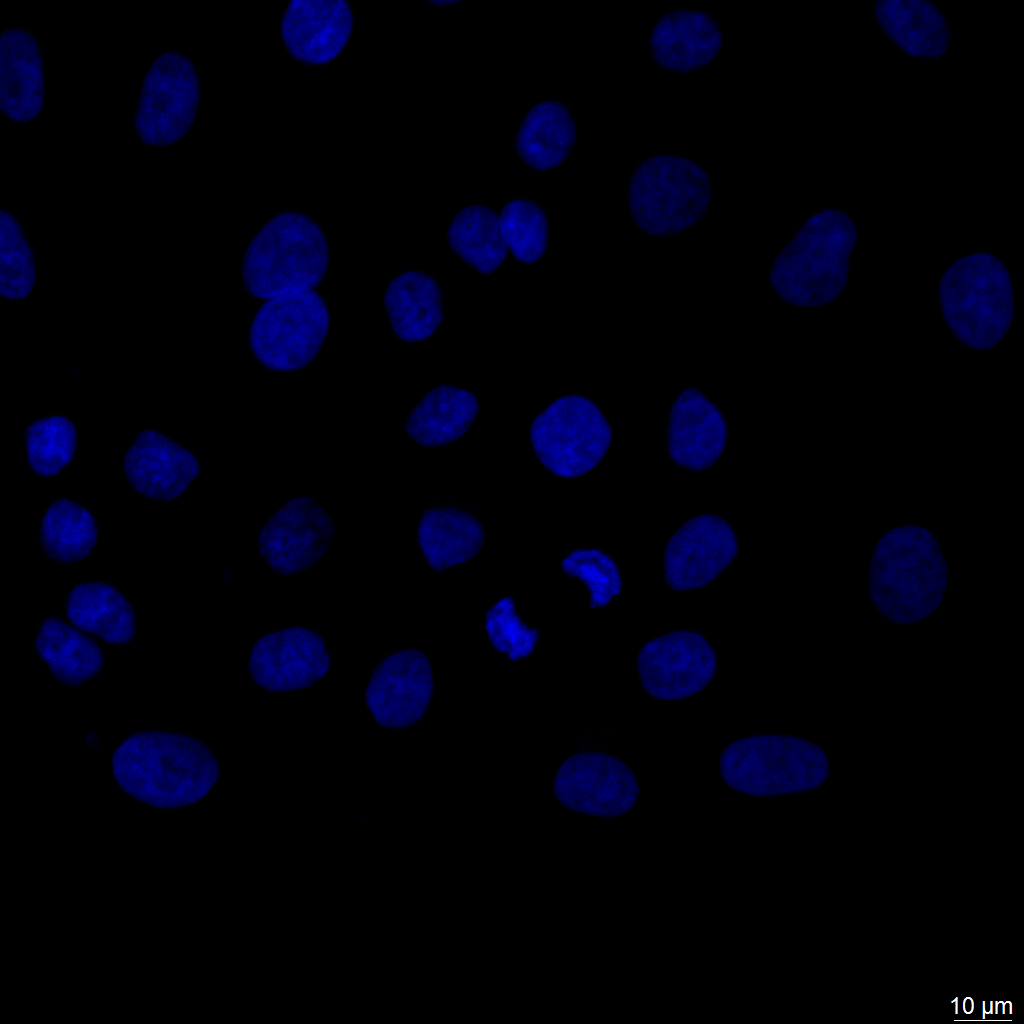

Supplement: Supplementary file 1 [file vetsci-12-00579-s001.zip › File S1/fig6/IF/LPS+MEL+ML385/24.11.8Nrf2_9_ch00_SV.tif]

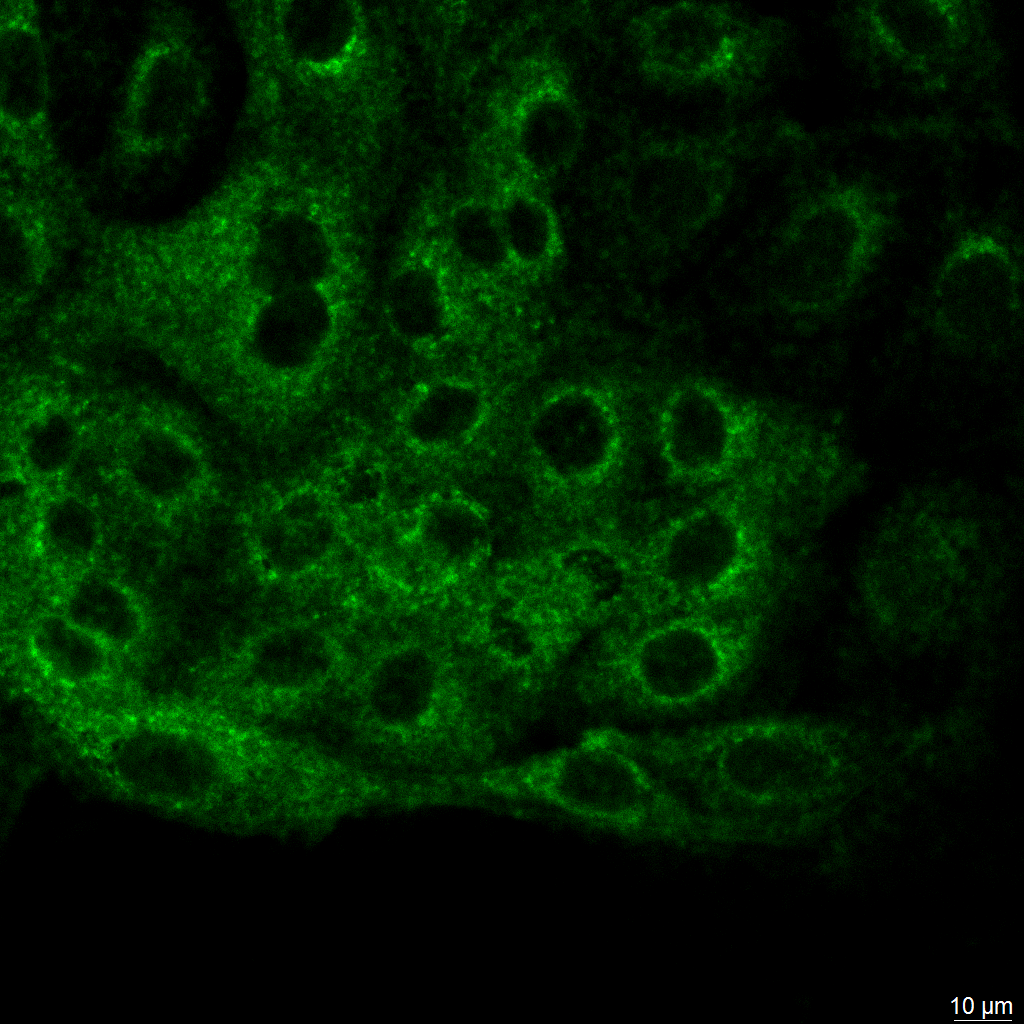

Supplement: Supplementary file 1 [file vetsci-12-00579-s001.zip › File S1/fig6/IF/LPS+MEL+ML385/24.11.8Nrf2_9_ch01_SV.tif]

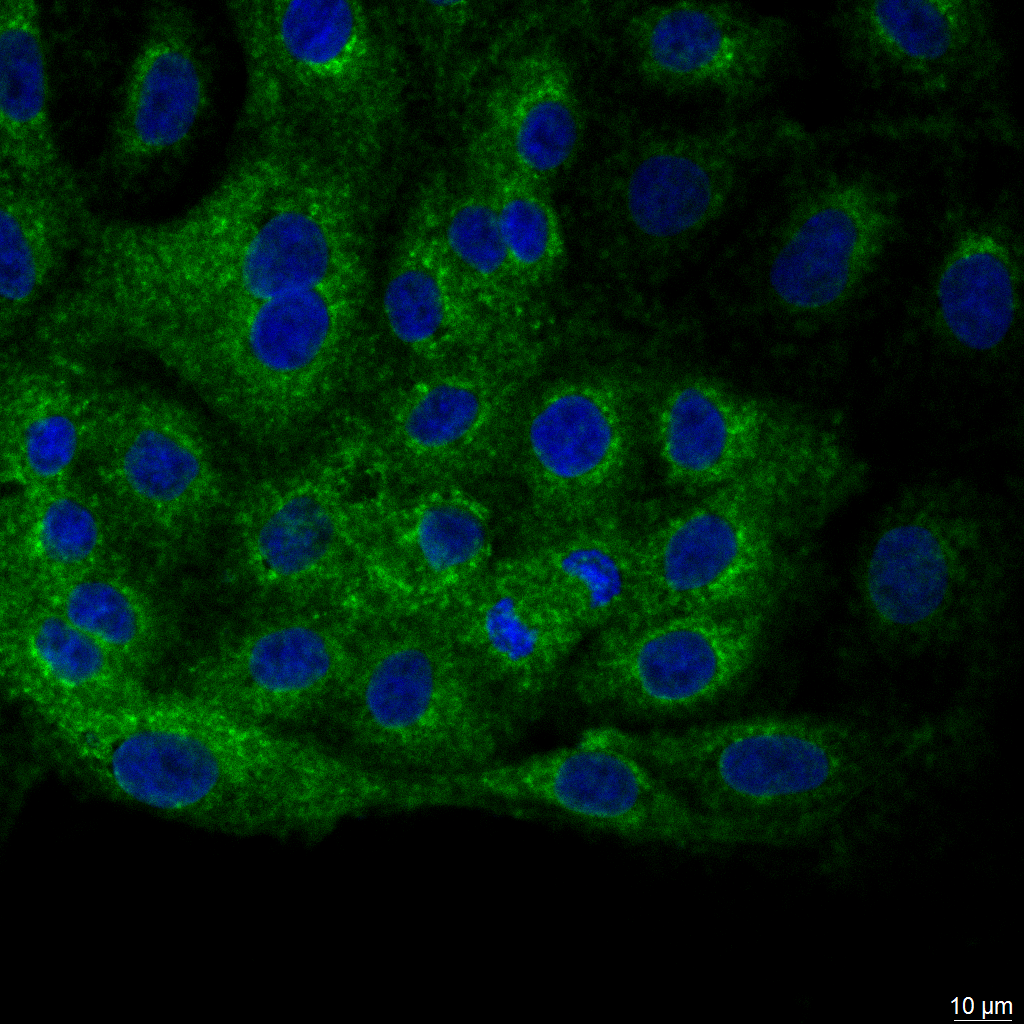

Supplement: Supplementary file 1 [file vetsci-12-00579-s001.zip › File S1/fig6/IF/LPS+MEL+ML385/24.11.8Nrf2_9_overlay.tif]

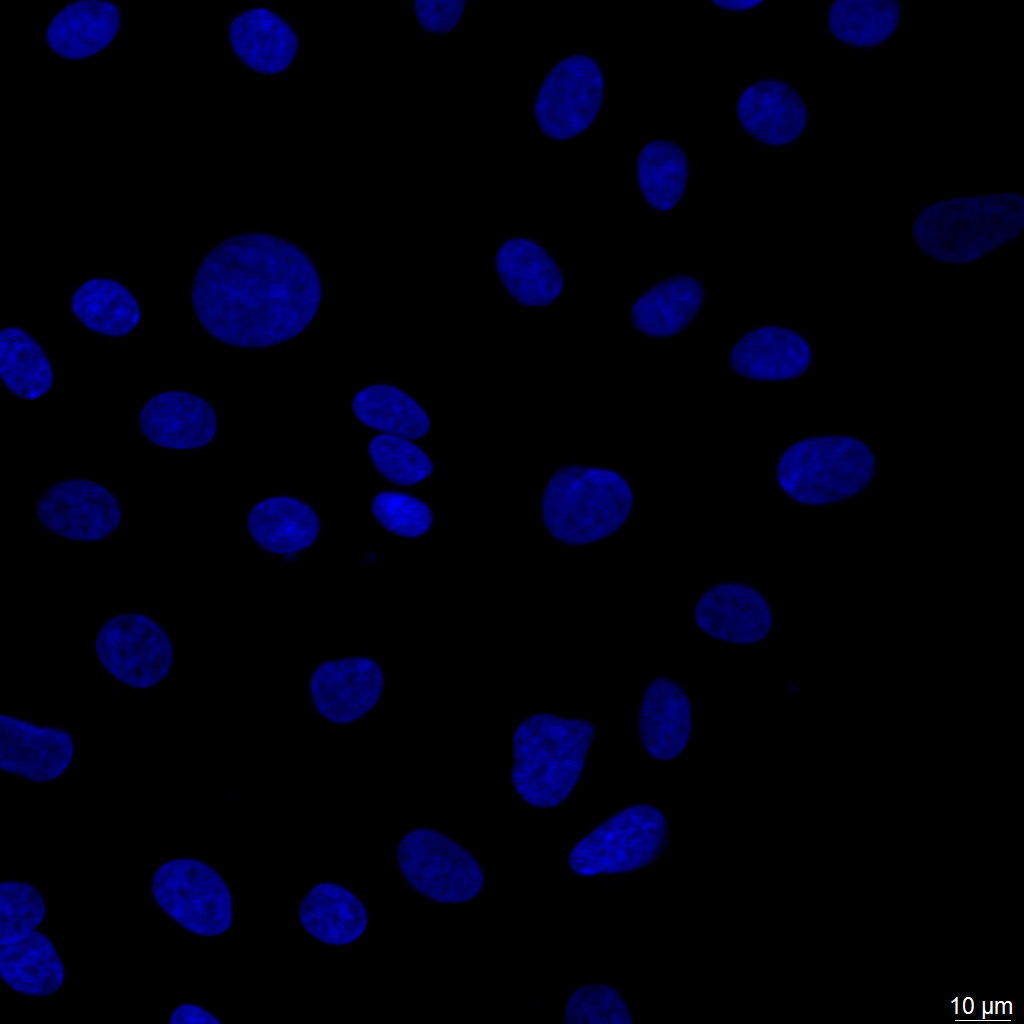

Supplement: Supplementary file 1 [file vetsci-12-00579-s001.zip › File S1/fig6/IF/LPS+MEL/2024.11.8 Nrf2_3_ch00_SV.tif]

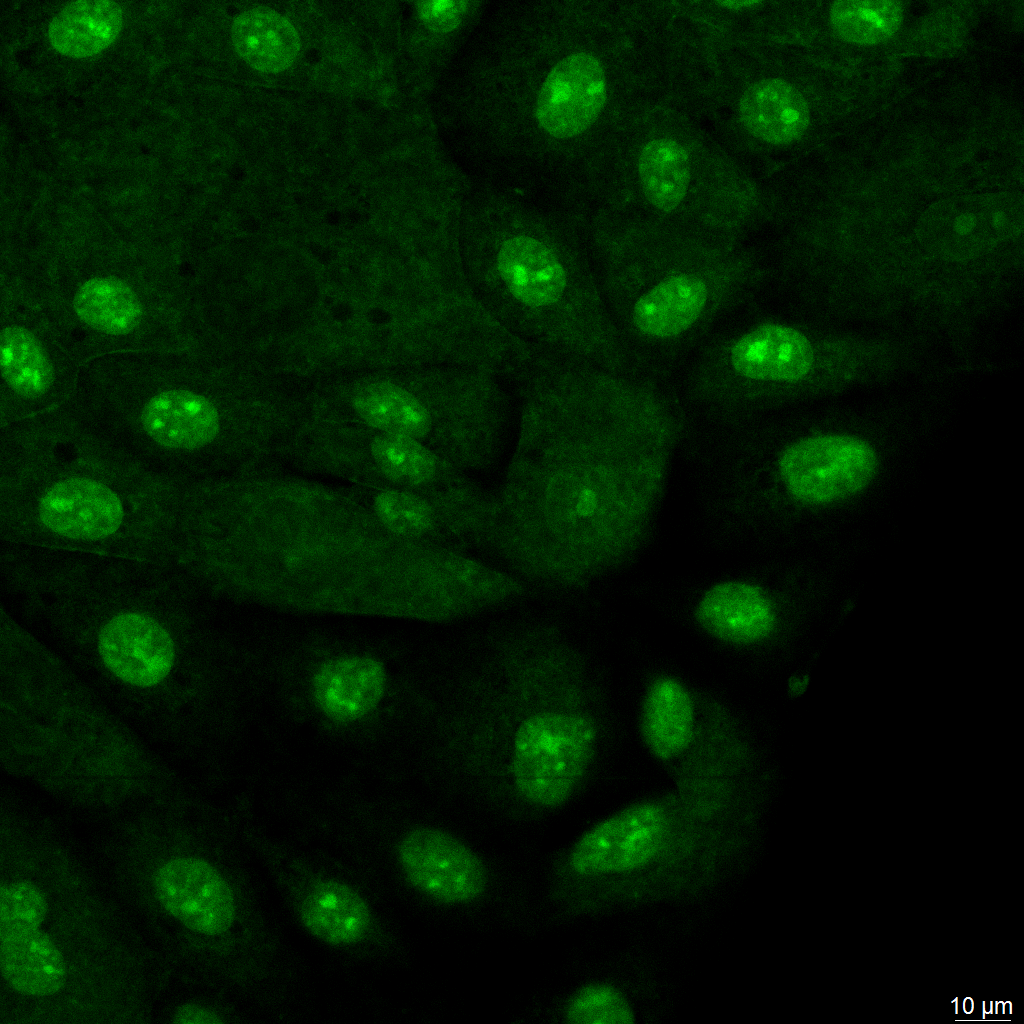

Supplement: Supplementary file 1 [file vetsci-12-00579-s001.zip › File S1/fig6/IF/LPS+MEL/2024.11.8 Nrf2_3_ch01_SV.tif]

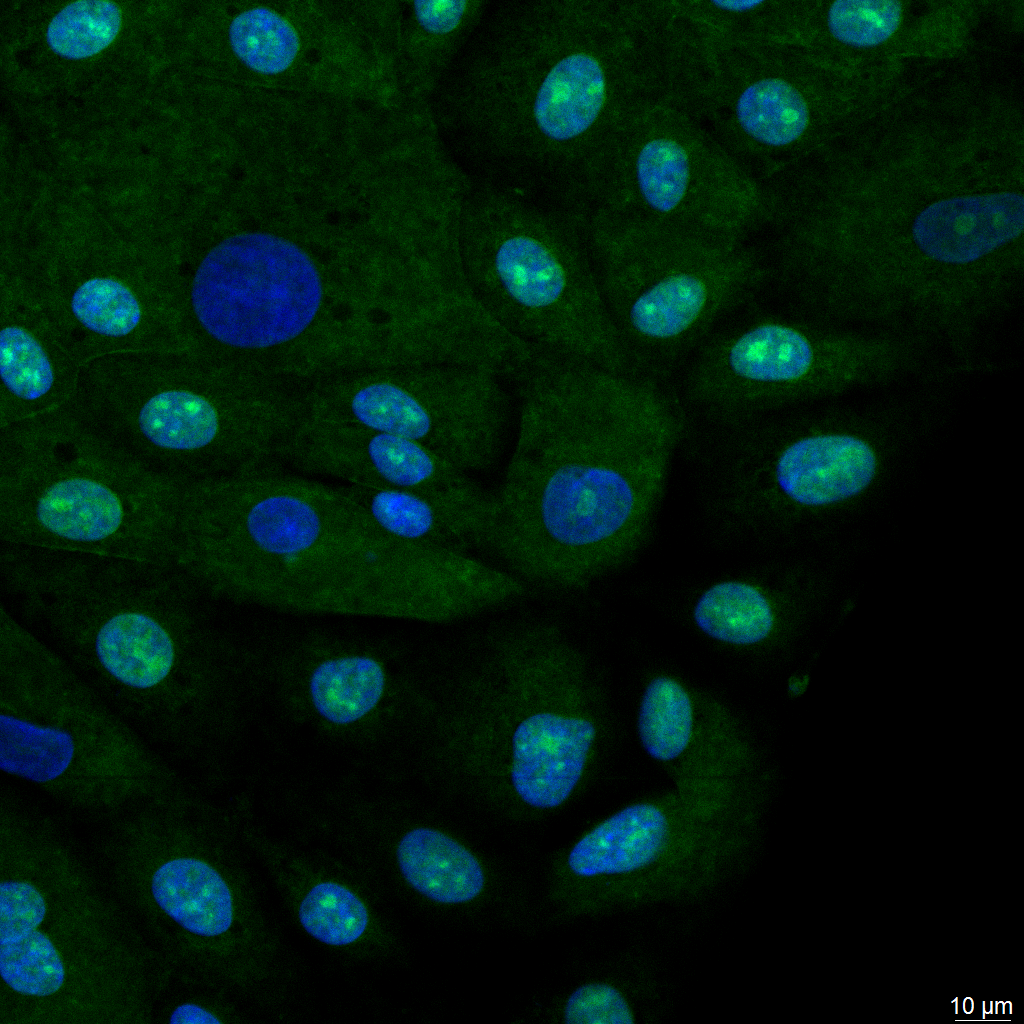

Supplement: Supplementary file 1 [file vetsci-12-00579-s001.zip › File S1/fig6/IF/LPS+MEL/2024.11.8 Nrf2_3_overlay.tif]

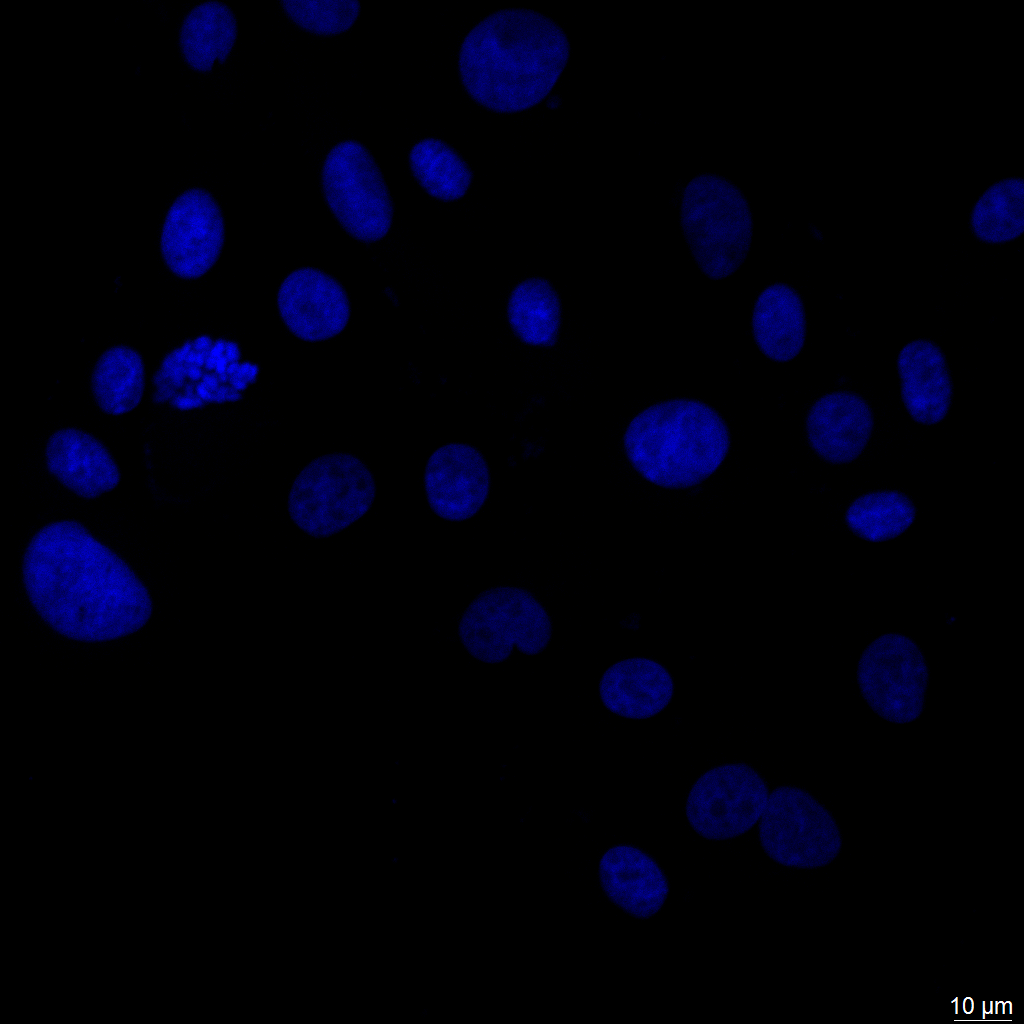

Supplement: Supplementary file 1 [file vetsci-12-00579-s001.zip › File S1/fig6/IF/LPS+ML385/24.11.8Nrf2_22_ch00_SV.tif]

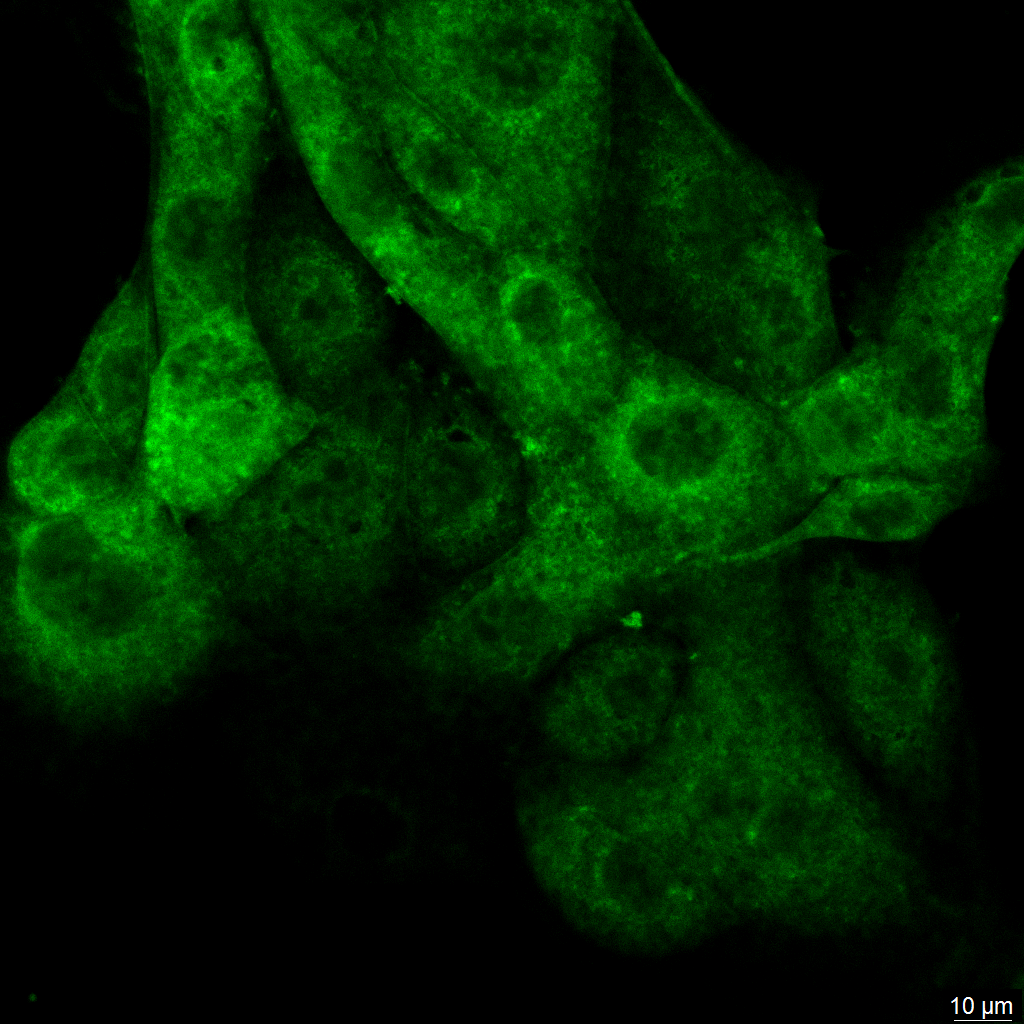

Supplement: Supplementary file 1 [file vetsci-12-00579-s001.zip › File S1/fig6/IF/LPS+ML385/24.11.8Nrf2_22_ch01_SV.tif]

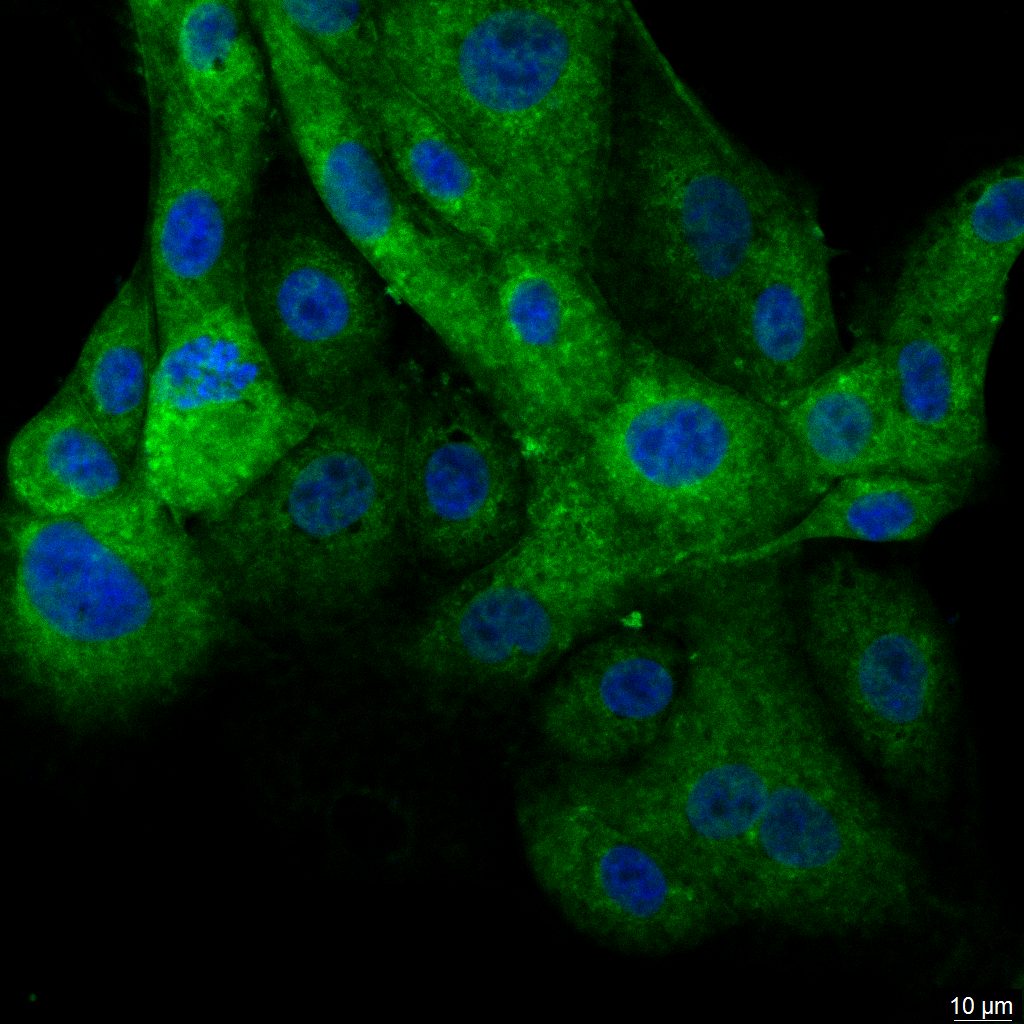

Supplement: Supplementary file 1 [file vetsci-12-00579-s001.zip › File S1/fig6/IF/LPS+ML385/24.11.8Nrf2_22_overlay.tif]

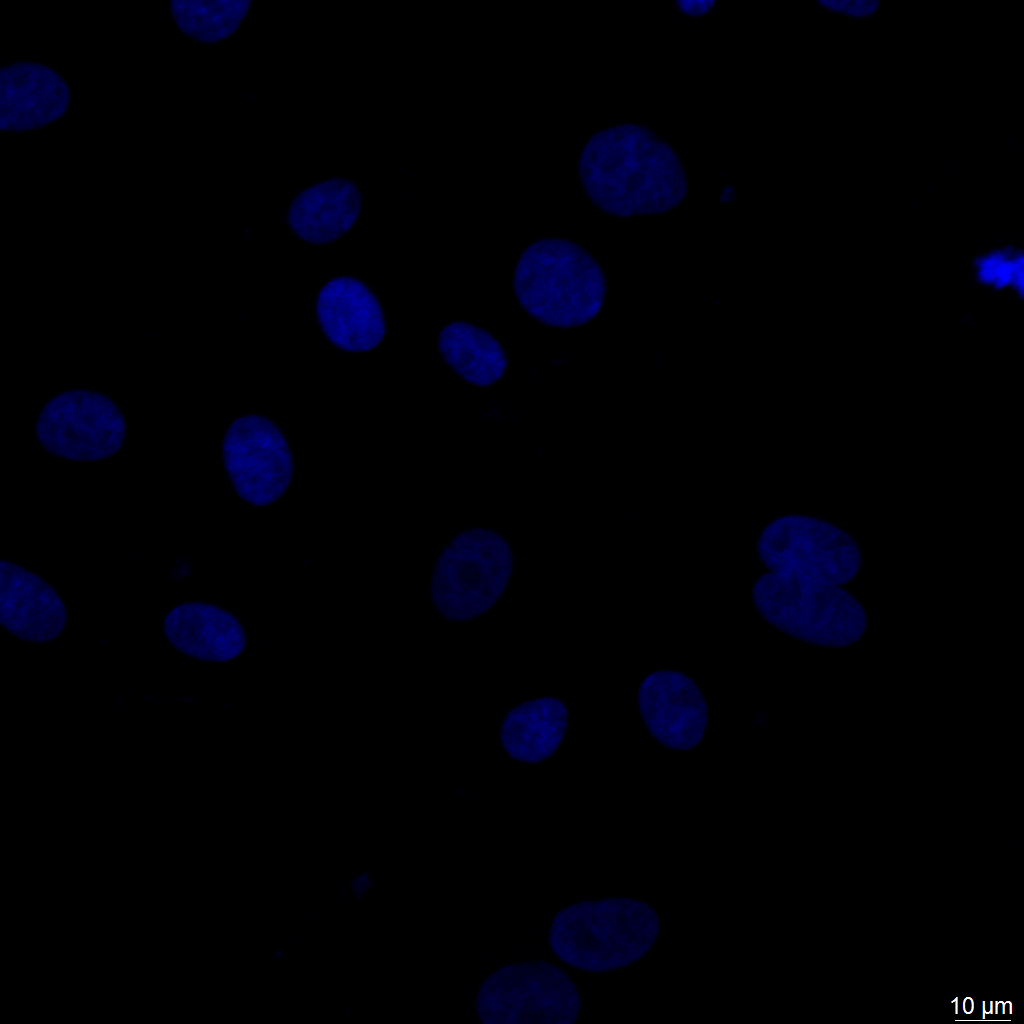

Supplement: Supplementary file 1 [file vetsci-12-00579-s001.zip › File S1/fig6/IF/L/Nrf211_30_ch00_SV.tif]

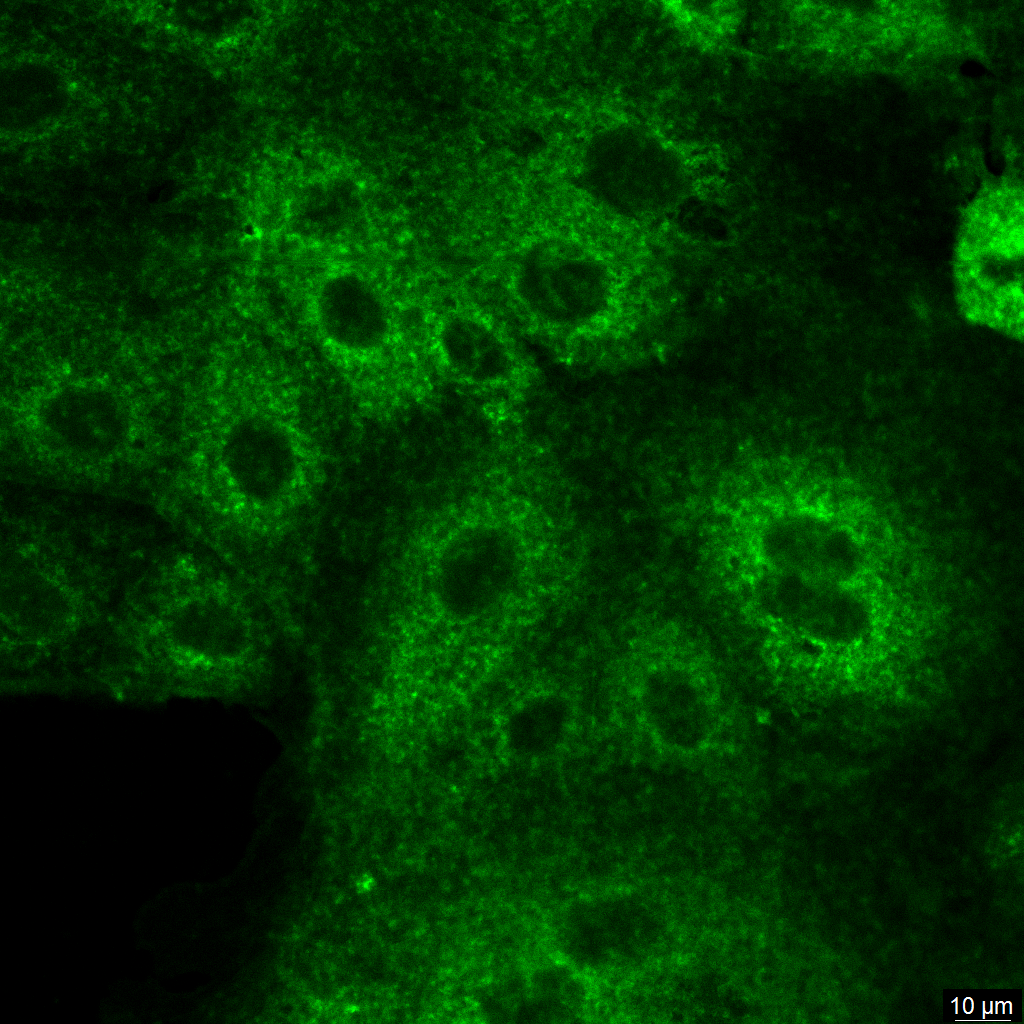

Supplement: Supplementary file 1 [file vetsci-12-00579-s001.zip › File S1/fig6/IF/L/Nrf211_30_ch01_SV.tif]

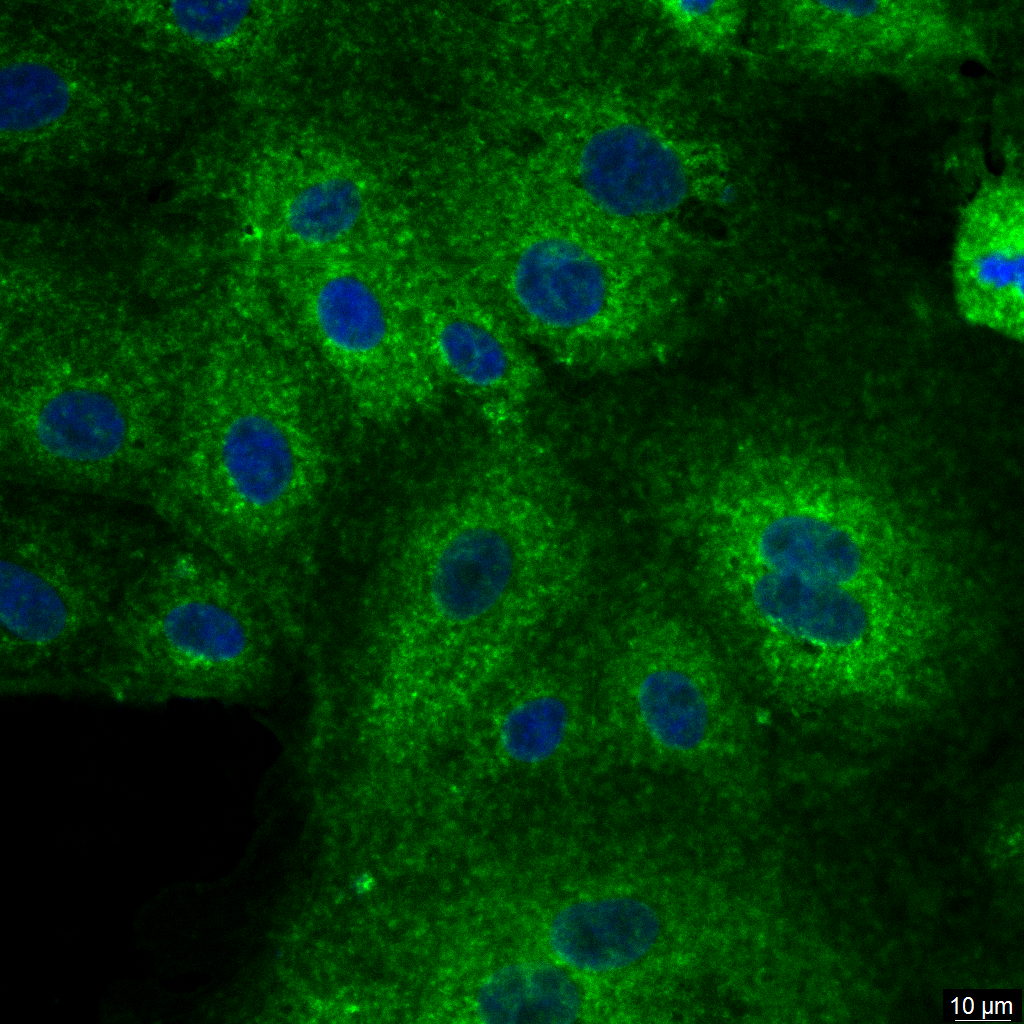

Supplement: Supplementary file 1 [file vetsci-12-00579-s001.zip › File S1/fig6/IF/L/Nrf211_30_overlay.tif]

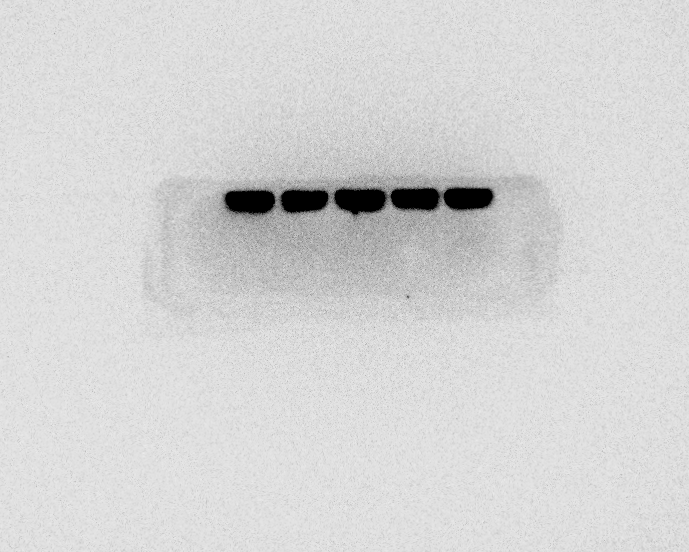

Supplement: Supplementary file 1 [file vetsci-12-00579-s001.zip › File S1/fig6/WB/BA_1_241025_174520_00.05.000_1_400.tif]

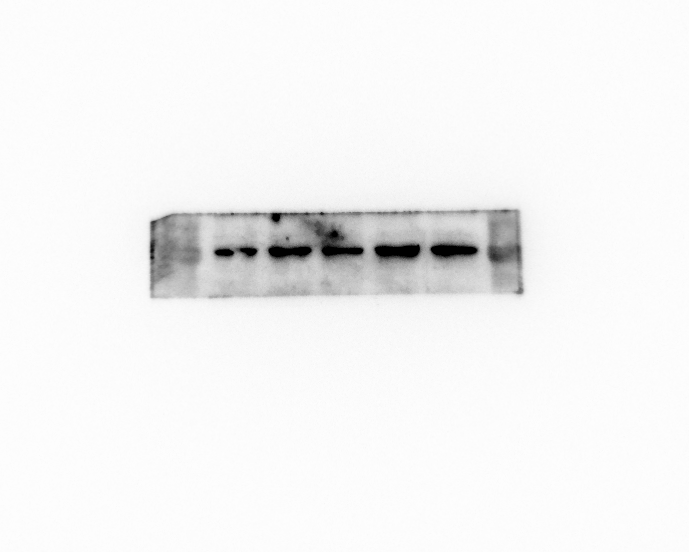

Supplement: Supplementary file 1 [file vetsci-12-00579-s001.zip › File S1/fig6/WB/COX2_1_241025_202013_00.22.000_1_3800.tif]

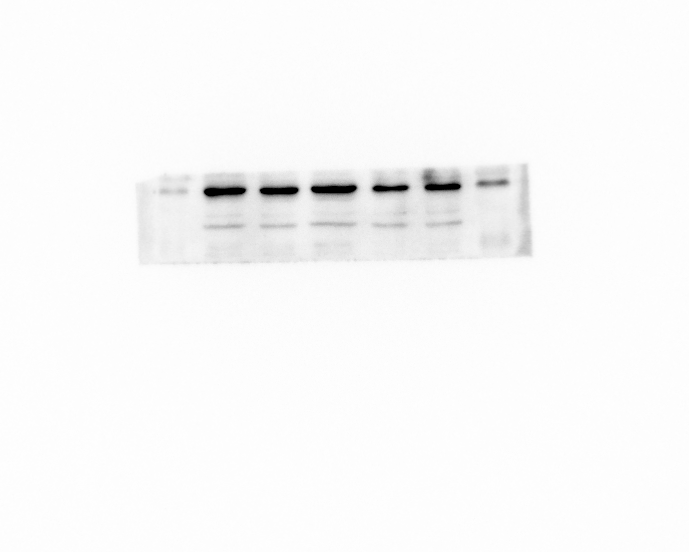

Supplement: Supplementary file 1 [file vetsci-12-00579-s001.zip › File S1/fig6/WB/ho1_1_240929_141948_00.05.000_1_5000.tif]

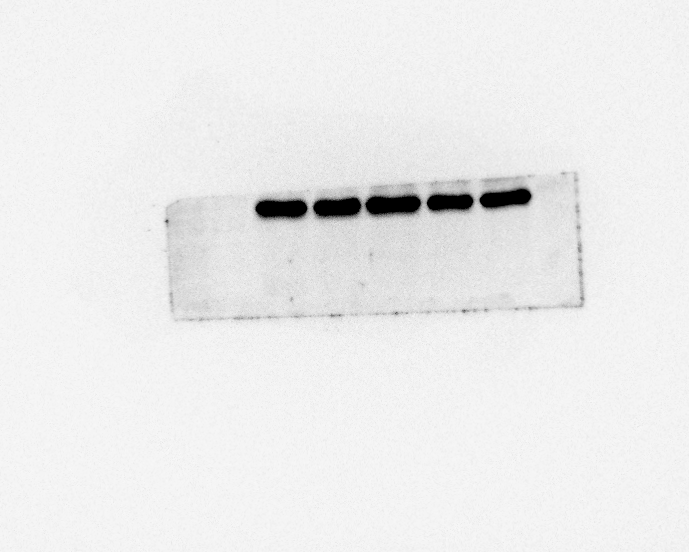

Supplement: Supplementary file 1 [file vetsci-12-00579-s001.zip › File S1/fig6/WB/LB1_1_241118_161627_00.06.000_1_1000.tif]

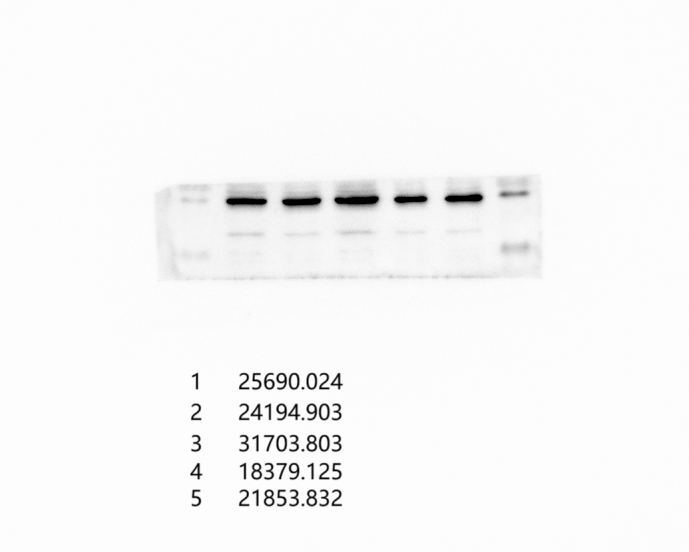

Supplement: Supplementary file 1 [file vetsci-12-00579-s001.zip › File S1/fig6/WB/nqo1_1_241001_192047_00.11.000_1_5000.tif]

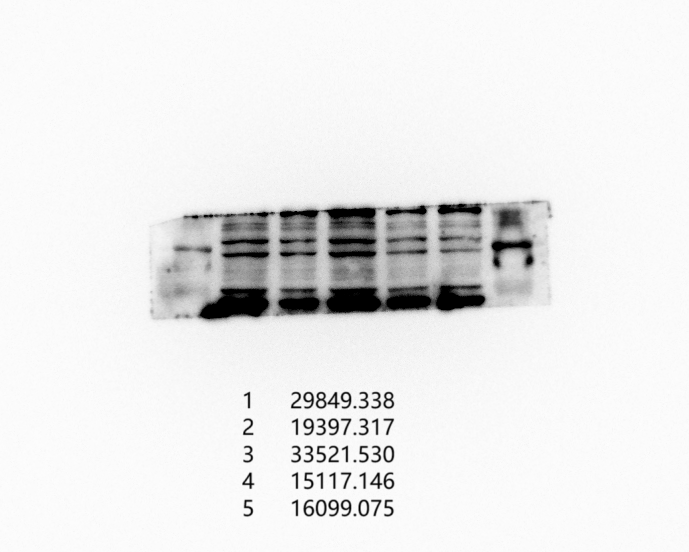

Supplement: Supplementary file 1 [file vetsci-12-00579-s001.zip › File S1/fig6/WB/NRF2_1_240929_135941_00.09.000_1_2400.tif]

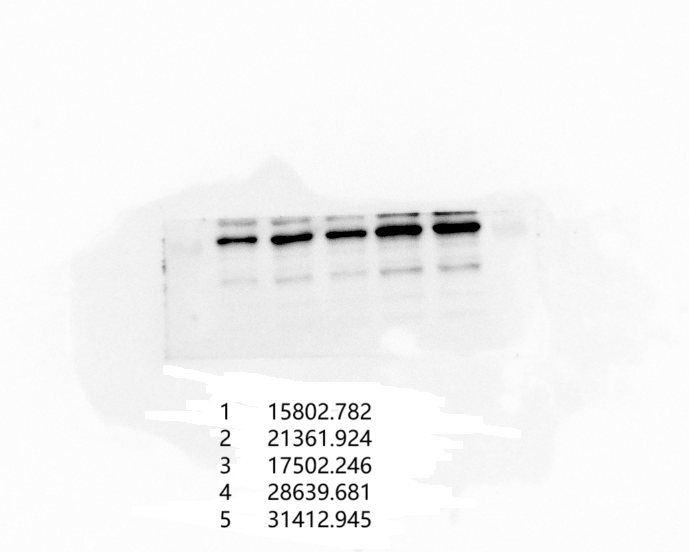

Supplement: Supplementary file 1 [file vetsci-12-00579-s001.zip › File S1/fig6/WB/P65_1_241120_104307_00.32.000_1_3400.tif]

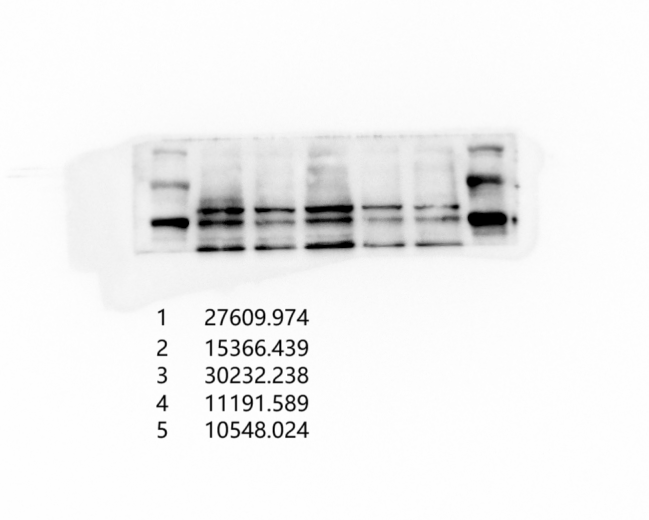

Supplement: Supplementary file 1 [file vetsci-12-00579-s001.zip › File S1/fig6/WB/║╦NRF2_1_241118_104639_01.40.000_1_6000.tif]
